# Supplementary material for: Probing altered receptor specificities of antigenically drifting human H3N2 viruses by chemoenzymatic synthesis, NMR, and modeling
Source: Nat Commun. 2024 Apr 6;15:2979. doi: 10.1038/s41467-024-47344-y (PMC10998905; doi:10.1038/s41467-024-47344-y)
Supplement: Supplementary file 1 — Supplementary Information [file 41467_2024_47344_MOESM1_ESM.pdf]

Supplementary Information

Probing Altered Receptor Specificities of Antigenically Drifting Human H3N2 Viruses by Chemoenzymatic Synthesis, NMR and Modeling

Luca Unione[1,2,3*], Augustinus N.A. Ammerlaan[1], Gerlof P Bosman[1], Elif Uslu[1], Ruonan Liang[1], Frederik Broszeit[1], Roosmarijn van der Woude[1], Yanyan Liu[1], Shengzhou Ma[4], Lin Liu[4], Marcos Gómez-Redondo[2], Iris A Bermejo[2], Pablo Valverde[2], Tammo Diercks[2], Ana Ardá[2,3], Robert P. de Vries[1*], Geert-Jan Boons[1,4,5,6*]

[1]Department of Chemical Biology & Drug Discovery, Utrecht Institute for Pharmaceutical Sciences, Utrecht University, 3584 CG Utrecht, The Netherlands; [2]CICbioGUNE, Basque Research & Technology Alliance (BRTA), Bizkaia Technology Park, Building 800, 48160 Derio, Bizkaia, Spain; [3]Ikerbasque, Basque Foundation for Science, Euskadi Plaza 5, 48009 Bilbao, Bizkaia, Spain; [4]Complex Carbohydrate Research Center, University of Georgia, 315 Riverbend Rd, Athens, GA 30602, USA; [5]Bijvoet Center for Biomolecular Research, Utrecht University, Utrecht, The Netherlands; [6]Department of Chemistry, University of Georgia, Athens, GA 30602, USA. *Correspondence to: lunione@cicbiogune.es, R.Vries@uu.nl, g.j.p.h.boons@uu.nl or gjboons@ccrc.uga.edu

Table of Contents Page

1.	Compounds preparation and characterization	S3
1.1.	NMR spectra (Supplementary Figs. 1-3)	S16
2.	Synthesis of compounds **6-11**	S25
3.	Glycan microarray (Supplementary Fig. 4)	S33
4.	NMR competition experiments (Supplementary Fig. 5 and 6)	S34
5.	2D STD-^{1}H, ^{13}C-HSQC NMR spectra	S39
5.1.	2D STD-^{1}H, ^{13}C-HSQC NMR spectra of compound **4** with HK68, NL91 and NL03 (Supplementary Fig. 7)	S39
5.2.	2D STD-^{1}H, ^{13}C-HSQC NMR spectra of compound **5** with HK68, NL91 and NL03 (Supplementary Fig. 8)	S40
6.	Details of 2D STD NMR results for the interaction of compound **5** with HA NL03 protein (Supplementary Fig. 9)	S41
7.	Details of the intermolecular interactions between **2** and the HAs proteins as derived by molecular modelling (Supplementary Fig. 10)	S42
8.	Snapshoot of the **2**/HK68 MD trajectory (Supplementary Fig. 11)	S43
9.	Snapshoot of the **2**/NL91 MD trajectory (Supplementary Fig. 12)	S43
10.	Snapshoot of the **2**/NL03 MD trajectory (Supplementary Fig. 13)	S44
11.	Glycan microarray (Supplementary Fig. 14)	S45
12.	References	S46

1. Compound preparation and characterization

Compound **8**

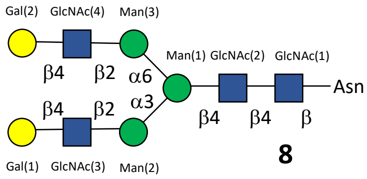

8

Compound **8** was prepared by a previously reported approach starting from Sialoglycopeptide [1].

^{1}H NMR (600 MHz, D_2O): δ = 5.03 (d, J < 2.0 Hz, 1H, H1-Man(2)), 4.99 (d, J = 9.8 Hz, 1H, H1-GlcNAc(1)), 4.83 (d, J < 2.0 Hz, 1H, H1-Man(3)), 4.69 (d, J = 2.6 Hz, 1H, H1-Man(1)), 4,53 (d, J = 7.6 Hz, 1H, H1-GlcNAc(2)), 4.50 (d, J = 7.8 Hz, 1H, H1-GlcNAc(3)), 4.47 (d, J = 7.8 Hz, 1H, H1-GlcNAc(4)), 4.38 (d, J = 7.7 Hz, 2H, H1-Gal(1), H1-Gal(2)), 4.17 (dd, J = 2.6 Hz, J = 6.5 Hz, 1H, H2-Man(1)), 4.11 (dd, J < 2.0 Hz, J = 2.6 Hz, H2-Man(2)), 4.00 (dd, J < 2.0 Hz, J = 2.6 Hz, H2-Man(3)), 3.97-3.30 (m, 52H, H2-H6-GlcNAc(1), H2-H6-GlcNAc(2), H3-H6-Man(1), H3-H6-Man(2), H3-H6-Man(3), H2-H6-GlcNAc(4), H2-H6-GlcNAc(3), H2-H6-Gal(1), H2-H6-Gal(2), αCH-Asn), 2.84-2.78 (m, 2H, βCH2-Asn), 1.99-1.95 (m, 12H, NHAc-GlcNAc(1), NHAc-GlcNAc(2), NHAc-GlcNAc(3), NHAc-GlcNAc(4) ppm.

^{13}C NMR (151 MHz, D_2O): d = 102.9, 101.3, 100.5, 99.5, 97.0, 80.4, 79.7, 78.7, 78.4, 78.1, 76.4, 76.2, 75.3, 75.2, 74.8, 74.4, 74.2, 73.5, 72.9, 72.3, 72.2, 72.0, 71.0, 70.1, 69.2, 68.6, 67.3, 65.9, 61.8, 61.5, 61.0, 60.0, 59.9, 55.0, 53.6, 50.9, 35.0, 22.4, 22.0 ppm.

ESI TOF-MS: for $C_{66}H_{110}N_6O_{48}$: m/z $[M-2H]^{2-}$; calcd: 876.3102; found: 876.3111.

Compound **9**

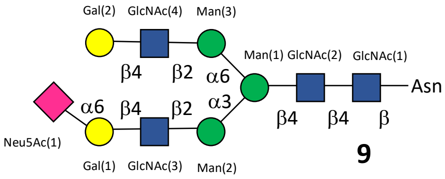

9

Compound **9** was prepared by a previously reported approach starting from compound **8** [2].

^{1}H NMR (600 MHz, D_2O): d = 5.03 (d, J < 2.0 Hz, 1H, H1-Man(2)), 4.99 (d, J = 9.8 Hz, 1H, H1-GlcNAc(1)), 4.83 (d, J < 2.0 Hz, 1H, H1-Man(3)), 4.69 (d, J = 2.6 Hz, 1H, H1-Man(1)), 4,53 (d, J = 7.6 Hz, 1H, H1-GlcNAc(2)), 4.50 (d, J = 7.8 Hz, 1H, H1-GlcNAc(3)), 4.47 (d, J = 7.8 Hz, 1H, H1-GlcNAc(4)), 4.38 (d, J = 7.7 Hz, 2H, H1-Gal(1), H1-Gal(2)), 4.17 (dd, J = 2.6 Hz, J = 6.5 Hz, 1H, H2-Man(1)), 4.11 (dd, J < 2.0 Hz, J = 2.6 Hz, H2-Man(2)), 4.00 (dd, J < 2.0 Hz, J = 2.6 Hz, H2-Man(3)), 3.97-3.30 (m, 59H, H2-H6-GlcNAc(1), H2-H6-GlcNAc(2), H3-H6-Man(1), H3-H6-Man(2), H3-H6-Man(3), H2-H6-GlcNAc(4), H2-H6-GlcNAc(3), H2-H6-Gal(1), H2-H6-Gal(2), H4-H9-Neu5Ac(1), αCH-Asn), 2.59 (dd, J = 12.2, J = 4.2, 1H, H3eq-Neu5Ac(1), 2.84-2.78 (m, 2H, βCH2-Asn), 1.99-1.95 (m, 15H, NHAc-GlcNAc(1), NHAc-GlcNAc(2), NHAc-GlcNAc(3), NHAc-GlcNAc(4), NHAc-Neu5Ac, 1.63 (t, J = 12.2, 1H, H3ax-Neu5Ac(1), ppm.

^{13}C NMR (151 MHz, D_2O): d = 102.9, 101.3, 100.5, 99.5, 97.0, 80.4, 79.7, 78.7, 78.4, 78.1, 76.4, 76.2, 75.3, 75.2, 74.8, 74.4, 74.2, 73.5, 72.9, 72.5, 72.3, 72.2, 72.0, 71.7, 71.0, 70.1, 69.2, 68.6, 68.3, 68.2, 67.3, 65.9, 63.3, 62.6, 61.8, 61.5, 61.0, 60.0, 59.9, 55.0, 53.6, 51.8, 50.9, 40.0, 35.0, 22.4, 22.0 ppm.

ESI TOF-MS: for $C_{77}H_{124}N_7O_{56}$: m/z $[M-2H]^{2-}$; calcd: 1021.8580; found: 1021.8575.

Compound **10**

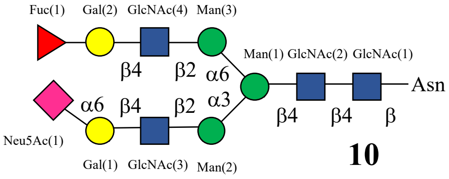

10

Compound **10** was prepared from **8** (12 mg, 6.8 μmol) using the general procedure for the installation of α2,6-Neu5Ac with ST6Gal1 and with Fut1 for the installation of α1,2 Fuc. After P2 column purification, **10** was obtained as a white solid (12.0 mg, 81%).

^{1}H NMR (600 MHz, D_2O): d = 5.22 (d, J = 2.0 Hz, 1H, H1-Fuc(1)), 5.03 (d, J < 2.0 Hz, 1H, H1-Man(2)), 4.99 (d, J = 9.8 Hz, 1H, H1-GlcNAc(1)), 4.83 (d, J < 2.0 Hz, 1H, H1-Man(3)), 4.69 (d, J = 2.6 Hz, 1H, H1-Man(1)), 4,53 (d, J = 7.6 Hz, 1H, H1-GlcNAc(2)), 4.50 (d, J = 7.8 Hz, 1H, H1-GlcNAc(3)), 4.47 (d, J = 7.8 Hz, 1H, H1-GlcNAc(4)), 4.38 (d, J = 7.7 Hz, 2H, H1-Gal(1), H1-Gal(2)), 4.17 (dd, J = 2.6 Hz, J = 6.5 Hz, 1H, H2-Man(1)), 4.13 (dd, J < 2.0 Hz, J = 6.7 Hz, 1H, H5-Fuc(1)), 4.11 (dd, J < 2.0 Hz, J = 2.6 Hz, H2-Man(2)), 4.00 (dd, J < 2.0 Hz, J = 2.6 Hz, H2-Man(3)), 3.97-3.30 (m, 62H, H2-H6-GlcNAc(1), H2-H6-GlcNAc(2), H3-H6-Man(1), H3-H6-Man(2), H3-H6-Man(3), H2-H6-GlcNAc(4), H2-H6-GlcNAc(3), H2-H6-Gal(1), H2-H6-Gal(2), H4-H9-Neu5Ac(1), H2-H4-Fuc(1), αCH-Asn), 2.59 (dd, J = 12.2, J = 4.2, 1H, H3eq-Neu5Ac(1), 2.84-2.78 (m, 2H, βCH2-Asn), 1.99-1.95 (m, 15H, NHAc-GlcNAc(1), NHAc-GlcNAc(2), NHAc-GlcNAc(3), NHAc-GlcNAc(4), NHAc-Neu5Ac, 1.63 (t, J = 12.2, 1H, H3ax-Neu5Ac(1), 1.15 (d, J = 6.7 Hz, 3H, H6-Fuc(1), ppm.

^{13}C NMR (151 MHz, D_2O): d = 102.9, 101.3, 100.5, 99.5, 99.4, 97.0, 80.4, 79.7, 78.7, 78.4, 78.1, 76.5, 76.4, 76.2, 75.3, 75.2, 74.8, 74.4, 74.2, 73.5, 72.9, 72.5, 72.3, 72.2, 72.0, 71.7, 71.0, 70.1, 69.5, 69.3, 69.2, 68.6, 68.3, 68.2, 67.3, 66.9, 65.9, 63.3, 62.6, 61.8, 61.5, 61.0, 60.0, 59.9, 55.0, 53.6, 51.8, 50.9, 40.0, 35.0, 22.4, 22.0, 15.5, ppm.

ESI TOF-MS: for $C_{83}H_{135}N_7O_{60}$: m/z $[M-2H]^{2-}$; calcd: 1095.3908; found: 1095.3925.

Preparation of compound 11

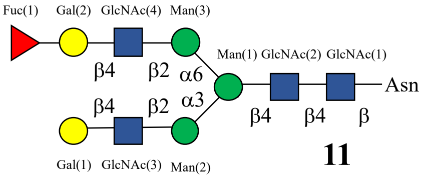

11

Compound **11** was prepared from **10** (12 mg, 5.4 μmol) by acid mediated hydrolysis of the α2,6-Neu5Ac according to the general procedure. Compound **10** was dissolved in 4 ml aqueous solution of acetic acid (2 M) and kept at 65 °C for 24 h. The solvent was removed by an N_2 flow. After purification by HPLC over an HILIC column, **11** was obtained as a white solid (6.5 mg, 45%).

^{1}H NMR (600 MHz, D_2O): d = 5.22 (d, J = 2.0 Hz, 1H, H1-Fuc(1)), 5.03 (d, J < 2.0 Hz, 1H, H1-Man(2)), 4.99 (d, J = 9.8 Hz, 1H, H1-GlcNAc(1)), 4.83 (d, J < 2.0 Hz, 1H, H1-Man(3)), 4.69 (d, J = 2.6 Hz, 1H, H1-Man(1)), 4,53 (d, J = 7.6 Hz, 1H, H1-GlcNAc(2)), 4.50 (d, J = 7.8 Hz, 1H, H1-GlcNAc(3)), 4.47 (d, J = 7.8 Hz, 1H, H1-GlcNAc(4)), 4.46 (d, J = 7.7 Hz, 1H, H1-Gal(2)), 4.38 (d, J = 7.6 Hz, 1H, H1-Gal(1)), 4.17 (dd, J = 2.6 Hz, J = 6.5 Hz, 1H, H2-Man(1)), 4.13 (dd, J < 2.0 Hz, J = 6.7 Hz, 1H, H5-Fuc(1)), 4.11 (dd, J < 2.0 Hz, J = 2.6 Hz, H2-Man(2)), 4.00 (dd, J < 2.0 Hz, J = 2.6 Hz, H2-Man(3)), 3.97-3.30 (m, 55H, H2-H6-GlcNAc(1), H2-H6-GlcNAc(2), H3-H6-Man(1), H3-H6-Man(2), H3-H6-Man(3), H2-H6-GlcNAc(4), H2-H6-GlcNAc(3), H2-H6-Gal(1), H2-H6-Gal(2), H2-H4-Fuc(1), αCH-Asn), 2.84-2.78 (m, 2H, βCH2-

Asn), 1.99-1.95 (m, 12H, NHAc-GlcNAc(1), NHAc-GlcNAc(2), NHAc-GlcNAc(3), NHAc-GlcNAc(4), 1.15 (d, J = 6.7 Hz, 3H, H6-Fuc(1), ppm.

^{13}C NMR (151 MHz, D_2O): d = 102.9, 101.3, 100.5, 99.5, 99.4, 97.0, 80.4, 79.7, 78.7, 78.4, 78.1, 76.5, 76.4, 76.2, 75.3, 75.2, 74.8, 74.4, 74.2, 73.5, 72.9, 72.3, 72.2, 72.0, 71.0, 70.1, 69.5, 69.3, 69.2, 68.6, 68.2, 67.3, 66.9, 65.9, 62.6, 61.8, 61.5, 61.0, 60.0, 59.9, 55.0, 53.6, 50.9, 35.0, 22.4, 22.0, 15.5, ppm.

ESI TOF-MS: for $C_{72}H_{118}N_6O_{52}$: m/z $[M-2H]^{2-}$; calcd: 949.3392; found: 949.3401.

Preparation of compounds 12 and 17

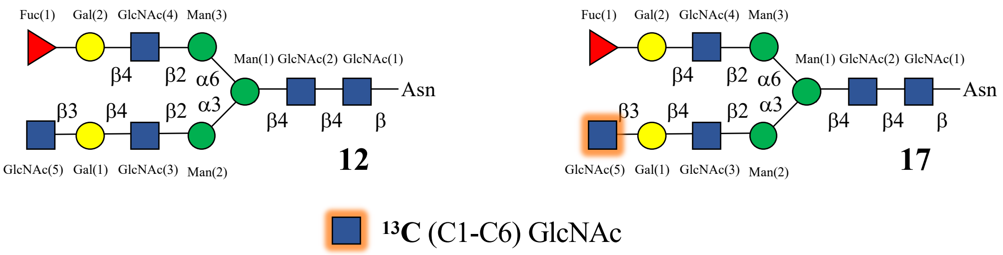

Compounds **12** and **17** were prepared by a previously reported approach starting from compound **11**.[2]

^1H NMR (600 MHz, D_2O): d = 5.22 (d, J = 2.0 Hz, 1H, H1-Fuc(1)), 5.03 (d, J < 2.0 Hz, 1H, H1-Man(2)), 4.99 (d, J = 9.8 Hz, 1H, H1-GlcNAc(1)), 4.83 (d, J < 2.0 Hz, 1H, H1-Man(3)), 4.69 (d, J = 2.6 Hz, 1H, H1-Man(1)), 4.59 (d, J = 9.6 Hz, 1H, H1-GlcNAc(5)), 4,53 (d, J = 7.6 Hz, 1H, H1-GlcNAc(2)), 4.50 (d, J = 7.8 Hz, 1H, H1-GlcNAc(3)), 4.47 (d, J = 7.8 Hz, 1H, H1-GlcNAc(4)), 4.46 (d, J = 7.7 Hz, 1H, H1-Gal(2)), 4.38 (d, J = 7.6 Hz, 1H, H1-Gal(1)), 4.17 (dd, J = 2.6 Hz, J = 6.5 Hz, 1H, H2-Man(1)), 4.13 (dd, J < 2.0 Hz, J = 6.7 Hz, 1H, H5-Fuc(1)), 4.11 (dd, J < 2.0 Hz, J = 2.6 Hz, H2-Man(2)), 4.00 (dd, J < 2.0 Hz, J = 2.6 Hz, H2-Man(3)), 3.97-3.30 (m, 61H, H2-H6-GlcNAc(1), H2-H6-GlcNAc(2), H3-H6-Man(1), H3-H6-Man(2), H3-H6-Man(3), H2-H6-GlcNAc(4), H2-H6-GlcNAc(3), H2-H6-Gal(1), H2-H6-Gal(2), H2-H4-Fuc(1), H2-H6-GlcNAc(5), αCH-Asn), 2.84-2.78 (m, 2H, βCH2-Asn), 1.99-1.95 (m, 15H, NHAc-GlcNAc(1), NHAc-GlcNAc(2), NHAc-GlcNAc(3), NHAc-GlcNAc(4), NHAc-GlcNAc(5) 1.15 (d, J = 6.7 Hz, 3H, H6-Fuc(1), ppm.

^{13}C NMR (151 MHz, D_2O): d = 102.9, 102.8, 101.3, 100.5, 100.2, 99.5, 99.4, 97.0, 82.0, 80.4, 79.7, 78.7, 78.4, 78.1, 76.5, 76.4, 76.2, 75.3, 75.2, 74.8, 74.4, 74.2, 73.5, 72.9, 72.3, 72.2, 72.0, 71.0, 70.1, 70.0, 69.5, 69.4, 69.3, 69.2, 68.6, 68.3, 68.2, 67.3, 66.9, 65.9, 62.6, 61.8, 61.5, 61.0, 60.0, 59.9, 55.7, 55.0, 53.6, 50.9, 35.0, 22.4, 22.0, 15.5, ppm.

ESI TOF-MS: for $C_{80}H_{133}N_7O_{57}$: m/z $[M-2H]^{2-}$; (**12**) calcd: 1050.8789; found: 1050.8767. (**17**) calcd: 1053.8890; found: 1053.8868.

Preparation of compounds 13 and 18

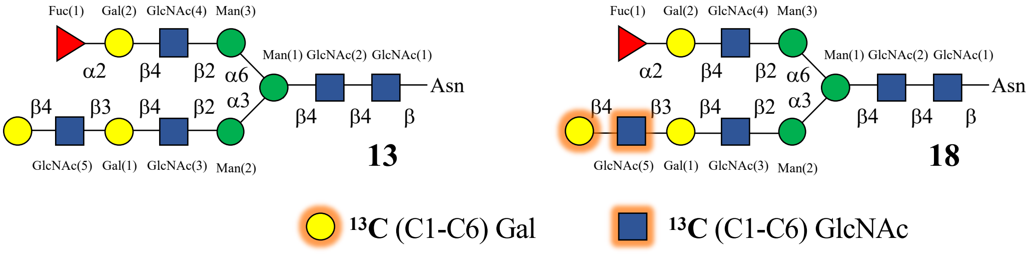

Compound **13** was prepared from **11** (5.0 mg, 2.6 µmol) using the general procedure for the installation of β1,3-GlcNAc with B3GnT2 and with B4GalT1 for the installation of β1,4-Gal. After P2 column purification, **13** was obtained as a white solid (1.9 mg, 82%).

Compound **18** was prepared from **11** (1.5 mg, 0.8 µmol) using the general procedure for the installation of β1,3-GlcNAc with B3GnT2 and B4GalT1 for the installation of β1,4-Gal. After P2 column purification, **18** was obtained as a white solid (1.5 mg, 83%).

^{1}H NMR (600 MHz, D_2O): d = 5.22 (d, J = 2.0 Hz, 1H, H1-Fuc(1)), 5.03 (d, J < 2.0 Hz, 1H, H1-Man(2)), 4.99 (d, J = 9.8 Hz, 1H, H1-GlcNAc(1)), 4.83 (d, J < 2.0 Hz, 1H, H1-Man(3)), 4.69 (d, J = 2.6 Hz, 1H, H1-Man(1)), 4.59 (d, J = 9.6 Hz, 1H, H1-GlcNAc(5)), 4,53 (d, J = 7.6 Hz, 1H, H1-GlcNAc(2)), 4.50 (d, J = 7.8 Hz, 1H, H1-GlcNAc(3)), 4.47 (d, J = 7.8 Hz, 1H, H1-GlcNAc(4)), 4.46 (d, J = 7.7 Hz, 1H, H1-Gal(2)), 4.38 (d, J = 7.6 Hz, 2H, H1-Gal(1), H1-Gal(3)), 4.17 (dd, J = 2.6 Hz, J = 6.5 Hz, 1H, H2-Man(1)), 4.13 (dd, J < 2.0 Hz, J = 6.7 Hz, 1H, H5-Fuc(1)), 4.11 (dd, J < 2.0 Hz, J = 2.6 Hz, H2-Man(2)), 4.00 (dd, J < 2.0 Hz, J = 2.6 Hz, H2-Man(3)), 3.97-3.30 (m, 67H, H2-H6-GlcNAc(1), H2-H6-GlcNAc(2), H3-H6-Man(1), H3-H6-Man(2), H3-H6-Man(3), H2-H6-GlcNAc(4), H2-H6-GlcNAc(3), H2-H6-Gal(1), H2-H6-Gal(2), H2-H4-Fuc(1), H2-H6-GlcNAc(5), H2-H6-Gal(3), αCH-Asn), 2.84-2.78 (m, 2H, βCH2-Asn), 1.99-1.95 (m, 15H, NHAc-GlcNAc(1), NHAc-GlcNAc(2), NHAc-GlcNAc(3), NHAc-GlcNAc(4), NHAc-GlcNAc(5) 1.15 (d, J = 6.7 Hz, 3H, H6-Fuc(1), ppm.

^{13}C NMR (151 MHz, D_2O): d = 102.9, 102.8, 101.3, 100.5, 100.2, 99.5, 99.4, 97.0, 82.0, 80.4, 79.7, 78.7, 78.4, 78.1, 77.8, 76.5, 76.4, 76.2, 75.3, 75.2, 74.8, 74.4, 74.3, 74.2, 73.5, 72.9, 72.3, 72.2, 72.0, 71.0, 70.1, 70.0, 69.5, 69.4, 69.3, 69.2, 68.6, 68.3, 68.2, 67.3, 66.9, 65.9, 62.6, 61.8, 61.5, 61.0, 60.0, 59.9, 59.7, 55.7, 55.0, 53.6, 50.9, 35.0, 22.4, 22.0, 15.5, ppm.

ESI TOF-MS: for $C_{86}H_{142}N_7O_{62}$: m/z $[M-2H]^{2-}$; (**13**) calcd: 1131.4014; found: 1131.4002. (**18**) calcd: 1137.4215; found: 1137.4208.

Preparation of compounds 14 and 19

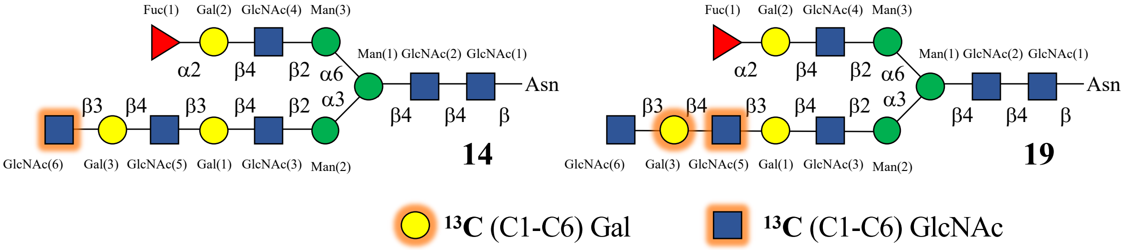

Compounds **14** and **19** were prepared by a previously reported approach starting from compound **13** and **18**, respectively.[2]

^{1}H NMR (600 MHz, D_2O): d = 5.22 (d, J = 2.0 Hz, 1H, H1-Fuc(1)), 5.03 (d, J < 2.0 Hz, 1H, H1-Man(2)), 4.99 (d, J = 9.8 Hz, 1H, H1-GlcNAc(1)), 4.83 (d, J < 2.0 Hz, 1H, H1-Man(3)), 4.69 (d, J = 2.6 Hz, 1H, H1-Man(1)), 4.59 (d, J = 9.6 Hz, 2H, H1-GlcNAc(5), H1-GlcNAc(6)), 4,53 (d, J = 7.6 Hz, 1H, H1-GlcNAc(2)), 4.50 (d, J = 7.8 Hz, 1H, H1-GlcNAc(3)), 4.47 (d, J = 7.8 Hz, 1H, H1-GlcNAc(4)), 4.46 (d, J = 7.7 Hz, 1H, H1-Gal(2)), 4.38 (d, J = 7.6 Hz, 2H, H1-Gal(1), H1-Gal(3)), 4.17 (dd, J = 2.6 Hz, J = 6.5 Hz, 1H, H2-Man(1)), 4.13 (dd, J < 2.0 Hz, J = 6.7 Hz, 1H, H5-Fuc(1)), 4.11 (dd, J < 2.0 Hz, J = 2.6 Hz, H2-Man(2)), 4.06 (dd, J < 2.0 Hz, J = 2.4 Hz, 2H, H4-Gal(1), H4-Gal(3)), 4.00 (dd, J < 2.0 Hz, J = 2.6 Hz, H2-Man(3)), 3.97-3.30 (m, 72H, H2-H6-GlcNAc(1), H2-H6-GlcNAc(2), H3-H6-Man(1), H3-H6-Man(2), H3-H6-Man(3), H2-H6-GlcNAc(4), H2-H6-GlcNAc(3), H2-H6-Gal(1), H2-H6-Gal(2), H2-H4-Fuc(1), H2-H6-GlcNAc(5), H3-H6-Gal(3), H2-H6-GlcNAc(6), αCH-Asn), 2.84-2.78 (m, 2H, βCH2-Asn), 1.99-1.95 (m, 18H, NHAc-GlcNAc(1), NHAc-GlcNAc(2), NHAc-GlcNAc(3), NHAc-GlcNAc(4), NHAc-GlcNAc(5), NHAc-GlcNAc(6), 1.15 (d, J = 6.7 Hz, 3H, H6-Fuc(1), ppm.

^{13}C NMR (151 MHz, D_2O): d = 102.9, 102.8, 101.3, 100.5, 100.2, 99.5, 99.4, 97.0, 82.0, 80.4, 79.7, 78.7, 78.4, 78.1, 77.8, 76.5, 76.4, 76.2, 75.3, 75.2, 74.8, 74.4, 74.3, 74.2, 73.5, 72.9, 72.3, 72.2, 72.0, 71.0, 70.1, 70.0, 69.5, 69.4, 69.3, 69.2, 68.6, 68.3, 68.2, 67.3, 66.9, 65.9, 62.6, 61.8, 61.5, 61.0, 60.0, 59.9, 59.7, 55.7, 55.0, 53.6, 50.9, 35.0, 22.4, 22.0, 15.5, ppm.

ESI TOF-MS: for $C_{94}H_{156}N_8O_{67}$: m/z $[M-2H]^{2-}$; (**14**) calcd: 1235.9511; found: 1235.9492. (**19**) calcd: 1238.9612; found: 1238.9589.

Preparation of compounds 15 and 20

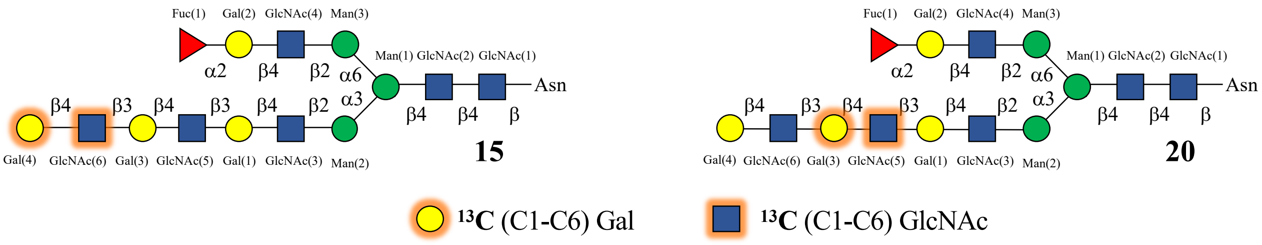

Compounds **15** and **20** were prepared by a previously reported approach starting from compound **14** and **19**, respectively.[2]

^{1}H NMR (600 MHz, D_2O): d = 5.22 (d, J = 2.0 Hz, 1H, H1-Fuc(1)), 5.03 (d, J < 2.0 Hz, 1H, H1-Man(2)), 4.99 (d, J = 9.8 Hz, 1H, H1-GlcNAc(1)), 4.83 (d, J < 2.0 Hz, 1H, H1-Man(3)), 4.69 (d, J = 2.6 Hz, 1H, H1-Man(1)), 4.59 (d, J = 9.6 Hz, 2H, H1-GlcNAc(5), H1-GlcNAc(6)), 4,53 (d, J = 7.6 Hz, 1H, H1-GlcNAc(2)), 4.50 (d, J = 7.8 Hz, 1H, H1-GlcNAc(3)), 4.47 (d, J = 7.8 Hz, 1H, H1-GlcNAc(4)), 4.46 (d, J = 7.7 Hz, 1H, H1-Gal(2)), 4.38 (d, J = 7.6 Hz, 3H, H1-Gal(1), H1-Gal(3), H1-Gal(4)), 4.17 (dd, J = 2.6 Hz, J = 6.5 Hz, 1H, H2-Man(1)), 4.13 (dd, J < 2.0 Hz, J = 6.7 Hz, 1H, H5-Fuc(1)), 4.11 (dd, J < 2.0 Hz, J = 2.6 Hz, H2-Man(2)), 4.06 (dd, J < 2.0 Hz, J = 2.4 Hz, 2H, H4-Gal(1), H4-Gal(3)), 4.00 (dd, J < 2.0 Hz, J = 2.6 Hz, H2-Man(3)), 3.97-3.30 (m, 78H, H2-H6-GlcNAc(1), H2-H6-GlcNAc(2), H3-H6-Man(1), H3-H6-Man(2), H3-H6-Man(3), H2-H6-GlcNAc(4), H2-H6-GlcNAc(3), H2-H6-Gal(1), H2-H6-Gal(2), H2-H4-Fuc(1), H2-H6-GlcNAc(5), H3-H6-Gal(3), H2-H6-GlcNAc(6), H2-H6-Gal(4), αCH-Asn), 2.84-2.78 (m, 2H, βCH2-

Asn), 1.99-1.95 (m, 18H, NHAc-GlcNAc(1), NHAc-GlcNAc(2), NHAc-GlcNAc(3), NHAc-GlcNAc(4), NHAc-GlcNAc(5), NHAc-GlcNAc(6), 1.15 (d, J = 6.7 Hz, 3H, H6-Fuc(1), ppm.

^{13}C NMR (151 MHz, D_2O): d = 102.9, 102.8, 101.3, 100.5, 100.2, 99.5, 99.4, 97.0, 82.0, 80.4, 79.7, 78.7, 78.4, 78.1, 77.8, 76.5, 76.4, 76.2, 75.3, 75.2, 74.8, 74.4, 74.3, 74.2, 73.5, 72.9, 72.3, 72.2, 72.0, 71.0, 70.1, 70.0, 69.5, 69.4, 69.3, 69.2, 68.6, 68.3, 68.2, 67.3, 66.9, 65.9, 62.6, 61.8, 61.5, 61.0, 60.0, 59.9, 59.7, 55.7, 55.0, 53.6, 50.9, 35.0, 22.4, 22.0, 15.5, ppm.

ESI TOF-MS: for $C_{100}H_{166}N_8O_{72}$: m/z $[M-2H]^{2-}$; (**15**) calcd: 1320.4915; found: 1320.4887. (**20**) calcd: 1320.4915; found: 1320.4902.

Preparation of compounds 16 and 21

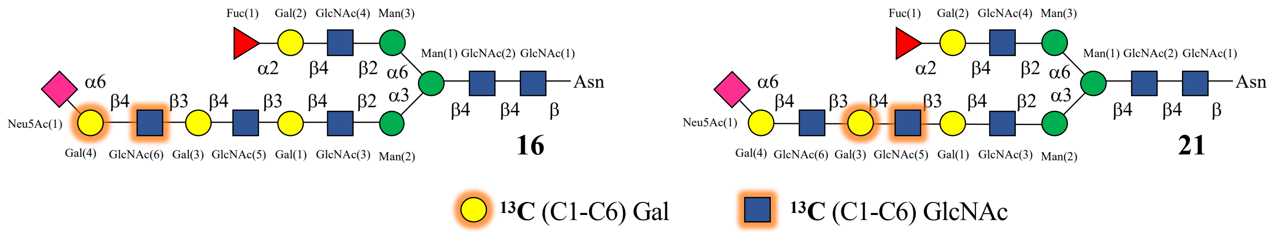

Compound **16** was prepared from **13** (1.9 mg, 0.84 µmol) using the general procedure for the installation of β1,3-GlcNAc with B3GnT2, and B4GalT1 for the installation of β1,4-Gal and ST6Gal1 for the installation of the α2,6-Neu5Ac. After P2 column purification, **16** was obtained as a white solid (0.7 mg, 29% over three steps).

Compound **21** was prepared from **18** (1.5 mg, 0.66 µmol) using the general procedure for the installation of β1,3-GlcNAc with B3GnT2 and B4GalT1 for the installation of β1,4-Gal and ST6Gal1 for the installation of the α2,6-Neu5Ac. After P2 column purification, **21** was obtained as a white solid (0.55 mg, 30% over three steps.

^{1}H NMR (600 MHz, D_2O): d = 5.22 (d, J = 2.0 Hz, 1H, H1-Fuc(1)), 5.03 (d, J < 2.0 Hz, 1H, H1-Man(2)), 4.99 (d, J = 9.8 Hz, 1H, H1-GlcNAc(1)), 4.83 (d, J < 2.0 Hz, 1H, H1-Man(3)), 4.69 (d, J = 2.6 Hz, 1H, H1-Man(1)), 4.59 (d, J = 9.6 Hz, 2H, H1-GlcNAc(5), H1-GlcNAc(6)), 4,53 (d, J = 7.6 Hz, 1H, H1-GlcNAc(2)), 4.50 (d, J = 7.8 Hz, 1H, H1-GlcNAc(3)), 4.47 (d, J = 7.8 Hz, 1H, H1-GlcNAc(4)), 4.46 (d, J = 7.7 Hz, 1H, H1-Gal(2)), 4.38 (d, J = 7.6 Hz, 3H, H1-Gal(1), H1-Gal(3)), 4.35 (d, J = 7.8 Hz, H1-Gal(4), 4.17 (dd, J = 2.6 Hz, J = 6.5 Hz, 1H, H2-Man(1)), 4.13 (dd, J < 2.0 Hz, J = 6.7 Hz, 1H, H5-Fuc(1)), 4.11 (dd, J < 2.0 Hz, J = 2.6 Hz, H2-Man(2)), 4.06 (dd, J < 2.0 Hz, J = 2.4 Hz, 3H, H4-Gal(1), H4-Gal(3), H4-Gal(4)), 4.00 (dd, J < 2.0 Hz, J = 2.6 Hz, H2-Man(3)), 3.97-3.30 (m, 85H, H2-H6-GlcNAc(1), H2-H6-GlcNAc(2), H3-H6-Man(1), H3-H6-Man(2), H3-H6-Man(3), H2-H6-GlcNAc(4), H2-H6-GlcNAc(3), H2-H6-Gal(1), H2-H6-Gal(2), H2-H4-Fuc(1), H2-H6-GlcNAc(5), H3-H6-Gal(3), H2-H6-GlcNAc(6), H2-H6-Gal(4), H4-H9-Neu5Ac(1), αCH-Asn), 2.84-2.78 (m, 2H, βCH2-Asn), 2.59 (dd, J = 12.2, J = 4.2, 1H, H3eq-Neu5Ac(1), 1.99-1.95 (m, 21H, NHAc-GlcNAc(1), NHAc-GlcNAc(2), NHAc-GlcNAc(3), NHAc-GlcNAc(4), NHAc-GlcNAc(5), NHAc-GlcNAc(6), NHAc-Neu5Ac(1), NHAc-Neu5Ac, 1.63 (t, J = 12.2, 1H, H3ax-Neu5Ac(1), 1.15 (d, J = 6.7 Hz, 3H, H6-Fuc(1), ppm.

^{13}C NMR (151 MHz, D_2O): d = 102.9, 102.8, 101.3, 100.5, 100.2, 99.5, 99.4, 97.0, 82.0, 80.4, 79.7, 78.7, 78.4, 78.1, 77.8, 76.5, 76.4, 76.2, 75.3, 75.2, 74.8, 74.4, 74.3, 74.2, 73.5, 72.9, 72.3, 72.2, 72.0, 71.0, 70.1, 70.0, 69.5, 69.4, 69.3, 69.2, 68.6, 68.3, 68.2, 67.3, 66.9, 65.9, 62.6, 61.8, 61.5, 61.0, 60.0, 59.9, 59.7, 55.7, 55.0, 53.6, 50.9, 35.0, 22.4, 22.0, 15.5, ppm.

ESI TOF-MS: for $C_{111}H_{182}N_9O_{80}$: m/z $[M-2H]^{2-}$; (**16**) calcd: 1466.0370; found: 1465.9885. (**21**) calcd: 1466.0370; found: 1466.0366.

Preparation of compound 22

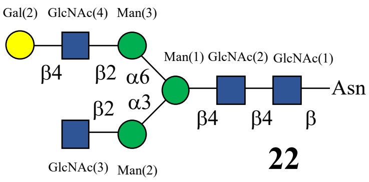

22

Compound **22** was prepared from **8** (5.0 mg, 2.8 µmol) by a previously reported procedure for the selective cleavage of galactoside with *E. coli* β-galactosidase.[2] After HPLC purification using a HILIC column, compound **22** was obtained as a white solid (3.1 mg, 71%).

^{1}H NMR (600 MHz, D_2O): d = 5.03 (d, J < 2.0 Hz, 1H, H1-Man(2)), 4.99 (d, J = 9.8 Hz, 1H, H1-GlcNAc(1)), 4.83 (d, J < 2.0 Hz, 1H, H1-Man(3)), 4.69 (d, J = 2.6 Hz, 1H, H1-Man(1)), 4,53 (d, J = 7.6 Hz, 1H, H1-GlcNAc(2)), 4.50 (d, J = 7.8 Hz, 1H, H1-GlcNAc(3)), 4.47 (d, J = 7.8 Hz, 1H, H1-GlcNAc(4)), 4.38 (d, J = 7.7 Hz, 1H, H1-Gal(2)), 4.17 (dd, J = 2.6 Hz, J = 6.5 Hz, 1H, H2-Man(1)), 4.11 (dd, J < 2.0 Hz, J = 2.6 Hz, H2-Man(2)), 4.00 (dd, J < 2.0 Hz, J = 2.6 Hz, H2-Man(3)), 3.97-3.30 (m, 46H, H2-H6-GlcNAc(1), H2-H6-GlcNAc(2), H3-H6-Man(1), H3-H6-Man(2), H3-H6-Man(3), H2-H6-GlcNAc(4), H2-H6-GlcNAc(3), H2-H6-Gal(2), αCH-Asn), 2.84-2.78 (m, 2H, βCH2-Asn), 1.99-1.95 (m, 12H, NHAc-GlcNAc(1), NHAc-GlcNAc(2), NHAc-GlcNAc(3), NHAc-GlcNAc(4) ppm.

^{13}C NMR (151 MHz, D_2O): d = 102.9, 101.3, 100.5, 99.5, 97.0, 80.4, 79.7, 78.7, 78.4, 78.1, 76.4, 76.2, 75.3, 75.2, 74.4, 74.2, 73.5, 72.9, 72.3, 72.2, 72.0, 71.0, 70.1, 69.2, 68.6, 67.3, 65.9, 61.8, 61.5, 61.0, 60.0, 55.0, 53.6, 50.9, 35.0, 22.4, 22.0 ppm.

ESI TOF-MS: for $C_{60}H_{100}N_6O_{43}$: m/z $[M-2H]^{2-}$; calcd: 795.2838; found: 795.2825.

Preparation of compound 23

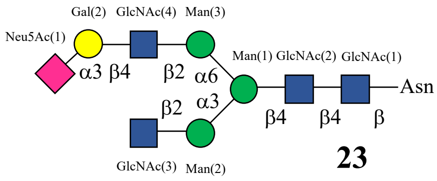

23

Compound **23** was prepared from **22** (3.0 mg, 1.9 µmol) using the general procedure for the installation of α2,3-Neu using ST3Gal4. After P2 column purification, compound **23** was obtained as a white solid (3.4 mg, 95%).

^{1}H NMR (600 MHz, D_2O): d = 5.03 (d, J < 2.0 Hz, 1H, H1-Man(2)), 4.99 (d, J = 9.8 Hz, 1H, H1-GlcNAc(1)), 4.83 (d, J < 2.0 Hz, 1H, H1-Man(3)), 4.69 (d, J = 2.6 Hz, 1H, H1-Man(1)), 4,53 (d, J = 7.6 Hz, 1H, H1-GlcNAc(2)), 4.50 (d, J = 7.8 Hz, 1H, H1-GlcNAc(3)), 4.47 (d, J = 7.8 Hz, 1H, H1-GlcNAc(4)), 4.43 (d, J = 7.7 Hz, 1H, H1-Gal(2)), 4.17 (dd, J = 2.6 Hz, J = 6.5 Hz, 1H, H2-Man(1)), 4.11 (dd, J < 2.0 Hz, J = 2.6 Hz, H2-Man(2)), 4.00 (dd, J < 2.0 Hz, J = 2.6 Hz, H2-

Man(3)), 3.99 (dd, J < 2.0 Hz, J = 2.5 Hz, H4-Gal(2)), 3.97-3.30 (m, 53H, H2-H6-GlcNAc(1), H2-H6-GlcNAc(2), H3-H6-Man(1), H3-H6-Man(2), H3-H6-Man(3), H2-H6-GlcNAc(4), H2-H6-GlcNAc(3), H2-H3, H5-H6-Gal(2), H4-H9 Neu5Ac, αCH-Asn), 2.84-2.78 (m, 2H, βCH2-Asn), 2.64 (dd, J = 12.4, J = 4.6, H3eq-Neu5Ac), 1.99-1.95 (m, 15H, NHAc-GlcNAc(1), NHAc-GlcNAc(2), NHAc-GlcNAc(3), NHAc-GlcNAc(4), NHAc-Neu5Ac, 1.68 (t, J = 12.4, H3ax-Neu5Ac) ppm.

^{13}C NMR (151 MHz, D_2O): d = 102.6, 101.3, 100.5, 99.5, 97.0, 80.4, 79.7, 78.7, 78.4, 78.1, 76.4, 76.2, 75.4, 75.2, 74.4, 74.2, 73.5, 72.8, 72.3, 72.2, 72.0, 71.7, 71.0, 70.1, 69.3, 68.6, 68.3, 67.6, 65.9, 62.3, 61.8, 61.5, 61.0, 60.0, 55.0, 53.6, 51.7, 50.9, 39.6, 35.0, 22.4, 22.0 ppm.

ESI TOF-MS: for $C_{71}H_{117}N_7O_{51}$: m/z $[M-2H]^{2-}$; calcd: 940.8315; found: 940.8298.

Preparation of compound 24

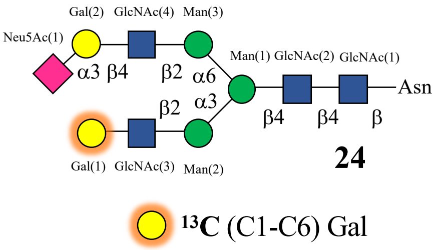

Compound **24** was prepared from **23** (3.4 mg, 1.8 µmol) using the general procedure for the installation of β1,3-Gal using B4GalT1. After P2 column purification, compound **24** was obtained as a white solid (2.9 mg, 81%).

^{1}H NMR (600 MHz, D_2O): d = 5.03 (d, J < 2.0 Hz, 1H, H1-Man(2)), 4.99 (d, J = 9.8 Hz, 1H, H1-GlcNAc(1)), 4.83 (d, J < 2.0 Hz, 1H, H1-Man(3)), 4.69 (d, J = 2.6 Hz, 1H, H1-Man(1)), 4,53 (d, J = 7.6 Hz, 1H, H1-GlcNAc(2)), 4.47 (d, J = 7.8 Hz, 2H, H1-GlcNAc(3), H1-GlcNAc(4)), 4.43 (d, J = 7.7 Hz, 1H, H1-Gal(2)), 4.38 (d, J = 7.6 Hz, 1H, H1-Gal(1)), 4.17 (dd, J = 2.6 Hz, J = 6.5 Hz, 1H, H2-Man(1)), 4.11 (dd, J < 2.0 Hz, J = 2.6 Hz, H2-Man(2)), 4.00 (dd, J < 2.0 Hz, J = 2.6 Hz, H2-Man(3)), 3.99 (dd, J < 2.0 Hz, J = 2.5 Hz, H4-Gal(2)), 3.97-3.30 (m, 59H, H2-H6-GlcNAc(1), H2-H6-GlcNAc(2), H3-H6-Man(1), H3-H6-Man(2), H3-H6-Man(3), H2-H6-GlcNAc(4), H2-H6-GlcNAc(3), H2-H3, H5-H6-Gal(2), H2-H6 -Gal(1), H4-H9 Neu5Ac, αCH-Asn), 2.84-2.78 (m, 2H, βCH2-Asn), 2.64 (dd, J = 12.4, J = 4.6, H3eq-Neu5Ac), 1.99-1.95 (m, 15H, NHAc-GlcNAc(1), NHAc-GlcNAc(2), NHAc-GlcNAc(3), NHAc-GlcNAc(4), NHAc-Neu5Ac, 1.68 (t, J = 12.4, H3ax-Neu5Ac) ppm.

^{13}C NMR (151 MHz, D_2O): d = 102.9, 102.6, 101.3, 100.5, 99.5, 97.0, 80.4, 79.7, 78.7, 78.4, 78.1, 76.4, 76.2, 75.4, 75.3, 75.2, 74.4, 74.2, 73.5, 72.8, 72.3, 72.2, 72.0, 71.7, 71.0, 70.1, 69.3, 68.6, 68.3, 67.6, 65.9, 62.3, 61.8, 61.5, 61.0, 60.0, 55.0, 53.6, 51.7, 50.9, 39.6, 35.0, 22.4, 22.0 ppm.

ESI TOF-MS: for $C_{77}H_{127}N_7O_{56}$: m/z $[M-2H]^{2-}$; calcd: 1024.8680; found: 1024.8655.

Preparation of compound 25

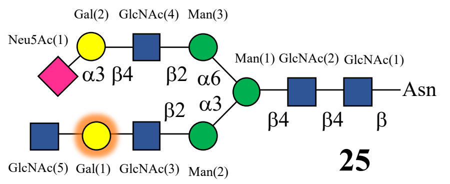

25

^{13}**C** (C1-C6) Gal

Compound **25** was prepared from **24** (2.9 mg, 1.45 µmol) using the general procedure for the installation of β1,4-GlcNAc using B3GnT2. After P2 column purification, compound **25** was obtained as a white solid (2.6 mg, 1.2 µmol, 83%).

^{1}H NMR (600 MHz, D_2O): d = 5.03 (d, J < 2.0 Hz, 1H, H1-Man(2)), 4.99 (d, J = 9.8 Hz, 1H, H1-GlcNAc(1)), 4.83 (d, J < 2.0 Hz, 1H, H1-Man(3)), 4.69 (d, J = 2.6 Hz, 1H, H1-Man(1)), 4.59 (d, J = 9.7 Hz, 1H, H1-GlcNAc(5)), 4,53 (d, J = 7.6 Hz, 1H, H1-GlcNAc(2)), 4.47 (d, J = 7.8 Hz, 2H, H1-GlcNAc(3), H1-GlcNAc(4)), 4.43 (d, J = 7.7 Hz, 1H, H1-Gal(2)), 4.38 (d, J = 7.6 Hz, 1H, H1-Gal(1)), 4.17 (dd, J = 2.6 Hz, J = 6.5 Hz, 1H, H2-Man(1)), 4.11 (dd, J < 2.0 Hz, J = 2.6 Hz, H2-Man(2)), 4.06 (dd, J < 2.0 Hz, J = 2.6 Hz, H4-Gal(1)), 4.00 (dd, J < 2.0 Hz, J = 2.6 Hz, H2-Man(3)), 3.99 (dd, J < 2.0 Hz, J = 2.5 Hz, H4-Gal(2)), 3.97-3.30 (m, 65H, H2-H6-GlcNAc(1), H2-H6-GlcNAc(2), H3-H6-Man(1), H3-H6-Man(2), H3-H6-Man(3), H2-H6-GlcNAc(4), H2-H6-GlcNAc(3), H2-H3, H5-H6-Gal(2), H2-H6 -Gal(1), H2-H6-GlcNAc(5), H4-H9 Neu5Ac, αCH-Asn), 2.84-2.78 (m, 2H, βCH2-Asn), 2.64 (dd, J = 12.4, J = 4.6, H3eq-Neu5Ac), 1.99-1.95 (m, 18H, NHAc-GlcNAc(1), NHAc-GlcNAc(2), NHAc-GlcNAc(3), NHAc-GlcNAc(4), NHAc-GlcNAc(5), NHAc-Neu5Ac, 1.68 (t, J = 12.4, H3ax-Neu5Ac) ppm.

^{13}C NMR (151 MHz, D_2O): d = 102.9, 102.8, 102.6, 101.3, 100.5, 99.5, 97.0, 82.0, 80.4, 79.7, 78.7, 78.4, 78.1, 76.4, 76.2, 75.4, 75.3, 75.2, 74.4, 74.2, 73.5, 72.8, 72.3, 72.2, 72.0, 71.7, 71.0, 70.1, 69.4, 69.3, 68.6, 68.3, 67.6, 65.9, 62.3, 61.8, 61.5, 61.0, 60.0, 55.7, 55.0, 53.6, 51.7, 50.9, 39.6, 35.0, 22.4, 22.0 ppm.

ESI TOF-MS: for $C_{85}H_{139}N_8O_{61}$: m/z $[M-2H]^{2-}$; calcd: 1126.4077; found: 1126.4074.

Preparation of compound 26

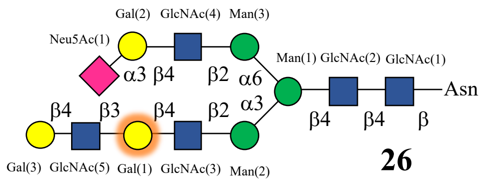

26

^{13}**C** (C1-C6) Gal

Compound **26** was prepared from **25** (2.6 mg, 1.2 µmol) using the general procedure for the installation of β1,3 Gal using B4GalT1. After P2 column purification, compound **26** was obtained as a white solid (2.6 mg, 92%).

^{1}H NMR (600 MHz, D_2O): d = 5.03 (d, J < 2.0 Hz, 1H, H1-Man(2)), 4.99 (d, J = 9.8 Hz, 1H, H1-GlcNAc(1)), 4.83 (d, J < 2.0 Hz, 1H, H1-Man(3)), 4.69 (d, J = 2.6 Hz, 1H, H1-Man(1)), 4.59 (d, J = 9.7 Hz, 1H, H1-GlcNAc(5)), 4,53 (d, J = 7.6 Hz, 1H, H1-GlcNAc(2)), 4.47 (d, J = 7.8 Hz, 2H, H1-GlcNAc(3), H1-GlcNAc(4)), 4.43 (d, J = 7.7 Hz, 1H, H1-Gal(2)), 4.38 (d, J = 7.6 Hz, 2H, H1-Gal(1), H1-Gal(3)), 4.17 (dd, J = 2.6 Hz, J = 6.5 Hz, 1H, H2-Man(1)), 4.11 (dd, J < 2.0 Hz, J = 2.6 Hz, H2-Man(2)), 4.06 (dd, J < 2.0 Hz, J = 2.6 Hz, H4-Gal(1)), 4.00 (dd, J < 2.0 Hz, J = 2.6 Hz, H2-Man(3)), 3.99 (dd, J < 2.0 Hz, J = 2.5 Hz, H4-Gal(2)), 3.97-3.30 (m, 71H, H2-H6-GlcNAc(1), H2-H6-GlcNAc(2), H3-H6-Man(1), H3-H6-Man(2), H3-H6-Man(3), H2-H6-GlcNAc(4), H2-H6-GlcNAc(3), H2-H3, H5-H6-Gal(2), H2-H6 -Gal(1), H2-H6-GlcNAc(5), H2-H6 -Gal(3), H4-H9 Neu5Ac, αCH-Asn), 2.84-2.78 (m, 2H, βCH2-Asn), 2.64 (dd, J = 12.4, J = 4.6, H3eq-Neu5Ac), 1.99-1.95 (m, 18H, NHAc-

GlcNAc(1), NHAc-GlcNAc(2), NHAc-GlcNAc(3), NHAc-GlcNAc(4), NHAc-GlcNAc(5), NHAc-Neu5Ac, 1.68 (t, J = 12.4, H3ax-Neu5Ac) ppm.

^{13}C NMR (151 MHz, D_2O): d = 102.9, 102.8, 102.6, 101.3, 100.5, 99.5, 97.0, 82.0, 80.4, 79.7, 78.7, 78.4, 78.1, 77.8, 76.4, 76.2, 75.4, 75.3, 75.2, 74.4, 74.3, 74.2, 73.5, 72.8, 72.3, 72.2, 72.0, 71.7, 71.0, 70.1, 69.4, 69.3, 68.6, 68.3, 67.6, 65.9, 62.3, 61.8, 61.5, 61.0, 60.0, 59.7, 55.7, 55.0, 53.6, 51.7, 50.9, 39.6, 35.0, 22.4, 22.0 ppm.

ESI TOF-MS: for $C_{91}H_{163}N_8O_{66}$: m/z $[M-2H]^{2-}$; calcd: 1207.4341; found: 1207.4311.

Preparation of compound 27

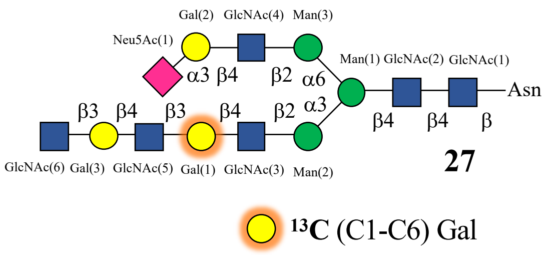

Compound **27** was prepared from **26** (2.6 mg, 1.1 µmol) using the general procedure for the installation of β1,4-GlcNAc using B3GnT2. After P2 column purification, compound **27** was obtained as a white solid (2.4 mg, 82%).

^{1}H NMR (600 MHz, D_2O): d = 5.03 (d, J < 2.0 Hz, 1H, H1-Man(2)), 4.99 (d, J = 9.8 Hz, 1H, H1-GlcNAc(1)), 4.83 (d, J < 2.0 Hz, 1H, H1-Man(3)), 4.69 (d, J = 2.6 Hz, 1H, H1-Man(1)), 4.59 (d, J = 9.7 Hz, 2H, H1-GlcNAc(5), H1-GlcNAc(6)), 4,53 (d, J = 7.6 Hz, 1H, H1-GlcNAc(2)), 4.47 (d, J = 7.8 Hz, 2H, H1-GlcNAc(3), H1-GlcNAc(4)), 4.43 (d, J = 7.7 Hz, 1H, H1-Gal(2)), 4.38 (d, J = 7.6 Hz, 2H, H1-Gal(1), H1-Gal(3)), 4.17 (dd, J = 2.6 Hz, J = 6.5 Hz, 1H, H2-Man(1)), 4.11 (dd, J < 2.0 Hz, J = 2.6 Hz, H2-Man(2)), 4.06 (dd, J < 2.0 Hz, J = 2.6 Hz, 2H, H4-Gal(1), H4-Gal(3)), 4.00 (dd, J < 2.0 Hz, J = 2.6 Hz, H2-Man(3)), 3.99 (dd, J < 2.0 Hz, J = 2.5 Hz, H4-Gal(2)), 3.97-3.30 (m, 76H, H2-H6-GlcNAc(1), H2-H6-GlcNAc(2), H3-H6-Man(1), H3-H6-Man(2), H3-H6-Man(3), H2-H6-GlcNAc(4), H2-H6-GlcNAc(3), H2-H3, H5-H6-Gal(2), H2-H6 -Gal(1), H2-H6-GlcNAc(5), H2-H6 -Gal(3), H2-H6 GlcNAc(6), H4-H9 Neu5Ac, αCH-Asn), 2.84-2.78 (m, 2H, βCH2-Asn), 2.64 (dd, J = 12.4, J = 4.6, H3eq-Neu5Ac), 1.99-1.95 (m, 21H, NHAc-GlcNAc(1), NHAc-GlcNAc(2), NHAc-GlcNAc(3), NHAc-GlcNAc(4), NHAc-GlcNAc(5), NHAc-GlcNAc(6), NHAc-Neu5Ac, 1.68 (t, J = 12.4, H3ax-Neu5Ac) ppm.

^{13}C NMR (151 MHz, D_2O): d = 102.9, 102.8, 102.6, 101.3, 100.5, 99.5, 97.0, 82.0, 80.4, 79.7, 78.7, 78.4, 78.1, 77.8, 76.4, 76.2, 75.4, 75.3, 75.2, 74.4, 74.3, 74.2, 73.5, 72.8, 72.3, 72.2, 72.0, 71.7, 71.0, 70.1, 69.4, 69.3, 68.6, 68.3, 67.6, 65.9, 62.3, 61.8, 61.5, 61.0, 60.0, 59.7, 55.7, 55.0, 53.6, 51.7, 50.9, 39.6, 35.0, 22.4, 22.0 ppm.

ESI TOF-MS: for $C_{99}H_{162}N_9O_{71}$: m/z $[M-2H]^{2-}$; calcd: 1309.4755; found: 1309.4721.

Preparation of compound 28

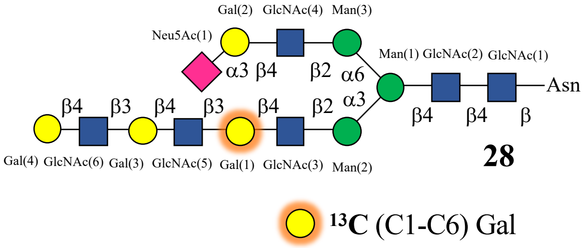

28 was prepared from **27** (2.4 mg, 0.9 μmol) using the general procedure for the installation of β1,3-Gal using B4GalT1. After P2 column purification, compound **28** was obtained as a white solid (2.2 mg, 89%).

^{1}H NMR (600 MHz, D_2O): d = 5.03 (d, J < 2.0 Hz, 1H, H1-Man(2)), 4.99 (d, J = 9.8 Hz, 1H, H1-GlcNAc(1)), 4.83 (d, J < 2.0 Hz, 1H, H1-Man(3)), 4.69 (d, J = 2.6 Hz, 1H, H1-Man(1)), 4.59 (d, J = 9.7 Hz, 2H, H1-GlcNAc(5), H1-GlcNAc(6)), 4,53 (d, J = 7.6 Hz, 1H, H1-GlcNAc(2)), 4.47 (d, J = 7.8 Hz, 2H, H1-GlcNAc(3), H1-GlcNAc(4)), 4.43 (d, J = 7.7 Hz, 1H, H1-Gal(2)), 4.38 (d, J = 7.6 Hz, 3H, H1-Gal(1), H1-Gal(3), H1-Gal(4)), 4.17 (dd, J = 2.6 Hz, J = 6.5 Hz, 1H, H2-Man(1)), 4.11 (dd, J < 2.0 Hz, J = 2.6 Hz, H2-Man(2)), 4.06 (dd, J < 2.0 Hz, J = 2.6 Hz, 2H, H4-Gal(1), H4-Gal(3)), 4.00 (dd, J < 2.0 Hz, J = 2.6 Hz, H2-Man(3)), 3.99 (dd, J < 2.0 Hz, J = 2.5 Hz, H4-Gal(2)), 3.97-3.30 (m, 82H, H2-H6-GlcNAc(1), H2-H6-GlcNAc(2), H3-H6-Man(1), H3-H6-Man(2), H3-H6-Man(3), H2-H6-GlcNAc(4), H2-H6-GlcNAc(3), H2-H3, H5-H6-Gal(2), H2-H6-Gal(1), H2-H6-GlcNAc(5), H2-H6 -Gal(3), H2-H6 GlcNAc(6), H4-H9 Neu5Ac, H2-H6-Gal(4) αCH-Asn), 2.84-2.78 (m, 2H, βCH2-Asn), 2.64 (dd, J = 12.4, J = 4.6, H3eq-Neu5Ac), 1.99-1.95 (m, 21H, NHAc-GlcNAc(1), NHAc-GlcNAc(2), NHAc-GlcNAc(3), NHAc-GlcNAc(4), NHAc-GlcNAc(5), NHAc-GlcNAc(6), NHAc-Neu5Ac, 1.68 (t, J = 12.4, H3ax-Neu5Ac) ppm.

^{13}C NMR (151 MHz, D_2O): d = 102.9, 102.8, 102.6, 101.3, 100.5, 99.5, 97.0, 82.0, 80.4, 79.7, 78.7, 78.4, 78.1, 77.8, 76.4, 76.2, 75.4, 75.3, 75.2, 74.4, 74.3, 74.2, 73.5, 72.8, 72.3, 72.2, 72.0, 71.7, 71.0, 70.1, 69.4, 69.3, 68.6, 68.3, 67.6, 65.9, 62.3, 61.8, 61.5, 61.0, 60.0, 59.7, 55.7, 55.0, 53.6, 51.7, 50.9, 39.6, 35.0, 22.4, 22.0 ppm.

ESI TOF-MS: for $C_{105}H_{173}N_9O_{76}$: m/z $[M-3H]^{3-}$; calcd: 926.6655; found: 926.6622.

Preparation of compound 29

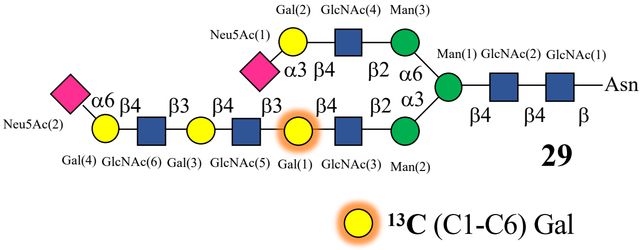

Compound **29** was prepared from **28** (2.2 mg, 0.8 μmol) using the general procedure for the installation of α2,6-Neu5Ac using ST6Gal1. After P2 column purification, compound **29** was obtained as a white solid (2.1 mg, 86%).

^{1}H NMR (600 MHz, D_2O): d = 5.03 (d, J < 2.0 Hz, 1H, H1-Man(2)), 4.99 (d, J = 9.8 Hz, 1H, H1-GlcNAc(1)), 4.83 (d, J < 2.0 Hz, 1H, H1-Man(3)), 4.69 (d, J = 2.6 Hz, 1H, H1-Man(1)), 4.59 (d, J = 9.7 Hz, 2H, H1-GlcNAc(5), H1-GlcNAc(6)), 4,53 (d, J = 7.6 Hz, 1H, H1-GlcNAc(2)), 4.47 (d, J = 7.8 Hz, 2H, H1-GlcNAc(3), H1-GlcNAc(4)), 4.43 (d, J = 7.7 Hz, 1H, H1-Gal(2)), 4.38 (d, J = 7.6 Hz, 2H, H1-Gal(1), H1-Gal(3)), 4.35 (d, J = 7.8 Hz, 1H, H1-Gal(4)), 4.17 (dd, J = 2.6 Hz, J = 6.5 Hz, 1H, H2-Man(1)), 4.11 (dd, J < 2.0 Hz, J = 2.6 Hz, H2-Man(2)), 4.06 (dd, J < 2.0 Hz, J = 2.6 Hz, 3H, H4-Gal(1), H4-Gal(3), H4-Gal(4)), 4.00 (dd, J < 2.0 Hz, J = 2.6 Hz, H2-Man(3)), 3.99 (dd, J < 2.0 Hz, J = 2.5 Hz, H4-Gal(2)), 3.97-

3.30 (m, 94H, H2-H6-GlcNAc(1), H2-H6-GlcNAc(2), H3-H6-Man(1), H3-H6-Man(2), H3-H6-Man(3), H2-H6-GlcNAc(4), H2-H6-GlcNAc(3), H2-H3, H5-H6-Gal(2), H2-H6-Gal(1), H2-H6-GlcNAc(5), H2-H6 -Gal(3), H2-H6 GlcNAc(6), H4-H9 Neu5Ac(1), H4-H9 Neu5Ac(2), H2-H3, H5-H6-Gal(4) αCH-Asn), 2.84-2.78 (m, 2H, βCH2-Asn), 2.64 (dd, J = 12.4, J = 4.6, H3eq-Neu5Ac(1), 2.54 (dd, J = 12.4, J = 4.6, H3eq-Neu5Ac(2), 1.99-1.95 (m, 24H, NHAc-GlcNAc(1), NHAc-GlcNAc(2), NHAc-GlcNAc(3), NHAc-GlcNAc(4), NHAc-GlcNAc(5), NHAc-GlcNAc(6), NHAc-Neu5Ac(1), NHAc-Neu5Ac(2), 1.68 (t, J = 12.4, H3ax-Neu5Ac(1), 1.58 (t, J = 12.4, H3ax-Neu5Ac(2) ppm.

^{13}C NMR (151 MHz, D_2O): d = 102.9, 102.8, 102.6, 101.3, 100.5, 99.5, 97.0, 82.0, 80.4, 79.7, 78.7, 78.4, 78.1, 77.8, 76.4, 76.2, 75.4, 75.3, 75.2, 74.4, 74.3, 74.2, 73.5, 72.8, 72.3, 72.2, 72.0, 71.7, 71.0, 70.1, 69.4, 69.3, 68.6, 68.3, 67.6, 67.2, 65.9, 62.3, 61.8, 61.6, 61.5, 61.0, 60.0, 59.7, 55.7, 55.0, 53.6, 51.7, 50.9, 50.8, 39.6, 39.1, 35.0, 22.4, 22.0 ppm.

ESI TOF-MS: for $C_{116}H_{190}N_{10}O_{84}$: m/z $[M-3H]^{3-}$; calcd: 1023.6973; found: 1023.6954.

Preparation and characterization of compound 2, 3, 4 and 5

Compound **3** was obtained from **16** (0.7 mg, 0.25 μmol) by using α1,2-fucosidase for the hydrolysis of the α1,2 Fuc. After P2 column purification, **3** was obtained as a white solid (0.6 mg, 96%).

Compound **4** was obtained from **21** (0.55 mg, 0.20 μmol) by using α1,2-fucosidase for the hydrolysis of the α1,2 Fuc. After P2 column purification, **4** was obtained as a white solid (0.45 mg, 90%).

Compound **5** was obtained from **29** (2.0 mg, 0.70 μmol) by selective hydrolysis of the α2,3-Neu5Ac. After P2 column purification, **5** was obtained as a white solid (1.45 mg, 74%).

NMR Nomenclature

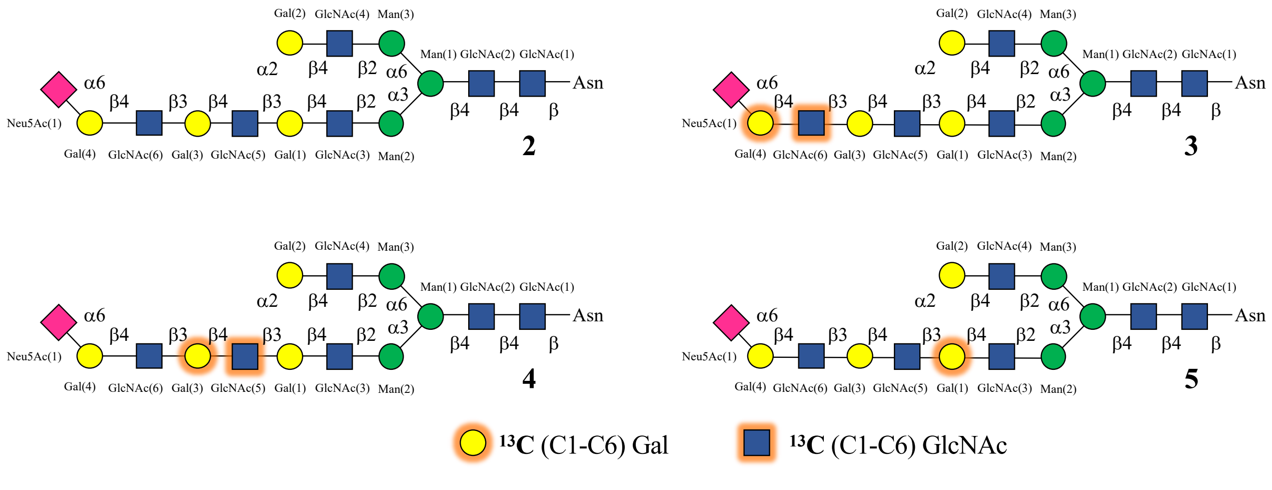

^{13}C; ^{1}H (600 MHz; D_2O). δ (ppm).

	C1-H1	C2-H2	C3;H3	C4;H4	C5-H5	C6-H6R;H6S	C7-H7	C8-H8	C9-H9	NHAc	Cα-Hα	Cβ-Hβ
**GlcNAc(1)**	78.1; 4.99	53.6; 3.78	72.2; 3.67	78.7; 3.58	76.2; 3.50	60.0; 3.56; 3.75	-[a]	-[a]	-[a]	22.4-22.0; 1.99-1.93 21H	-[a]	-[a]
**GlcNAc(2)**	101.3; 4.53	55.0; 3.71	73.5; 3.66	79.7; 3.65	74.4; 3.51	60.0; 3.56; 3.75	-[a]	-[a]	-[a]	22.4-22.0; 1.99-1.93 21H	-[a]	-[a]
**Man(1)**	100.5; 4.69	70.1; 4.17	80.4; 3.69	65.9; 3.69	74.2; 3.56	65.9; 3.86; 3.71	-[a]	-[a]	-[a]	-[a]	-[a]	-[a]
**Man(2)**	99.5; 5.03	76.4; 4.11	69.2; 3.81	67.3; 3.42	73.5; 3.66	61.5; 3.83; 3.53	-[a]	-[a]	-[a]	-[a]	-[a]	-[a]
**GlcNAc(3)**	99.5; 4.50	55.0; 3.67	75.3; 3.63	78.4; 3.65	74.8; 3.49	59.9; 3.89; 3.76	-[a]	-[a]	-[a]	22.4-22.0; 1.99-1.93 21H	-[a]	-[a]
**Gal(1)**	102.8; 4.32	69.8; 3.44	81.9; 3.59	68.2; 4.02	74.7; 3.58	60.9; 3.63; 3.59	-[a]	-[a]	-[a]	-[a]	-[a]	-[a]
**Man(3)**	97.0; 4.83	76.4; 4.00	69.2; 3.81	67.3; 3.41	72.9; 3.53	61.8; 3.83; 3.54	-[a]	-[a]	-[a]	-[a]	-[a]	-[a]
**GlcNAc(4)**	99.5; 4.50	55.0; 3.67	75.3; 3.63	78.4; 3.65	74.8; 3.49	59.9; 3.89; 3.76	-[a]	-[a]	-[a]	22.4-22.0; 1.99-1.93 21H	-[a]	-[a]
**Gal(2)**	102.9; 4.38	71.0; 3.45	72.3; 3.58	68.6; 3.84	72.0; 3.66	61.0; 3.66; 3.66	-[a]	-[a]	-[a]	-[a]	-[a]	-[a]
**GlcNAc(5)**	102.7; 4.56	55.0; 3.66	72.0; 3.59	77.9; 3.59	74.4; 3.45	59.72; 3.82; 3.71	-[a]	-[a]	-[a]	22.4-22.0; 1.99-1.93 21H	-[a]	-[a]
**Gal(3)**	102.8; 4.33	69.9; 3.44	81.9; 3.60	68.2; 4.02	74.7; 3.58	60.9; 3.63; 3.59	-[a]	-[a]	-[a]	-[a]	-[a]	-[a]
**GlcNAc(6)**	102.8; 4.55	54.8; 3.66	71.1; 3.65	79.3; 3.53	72.6; 3.69	59.0; 3.81; 3.71	-[a]	-[a]	-[a]	22.4-22.0; 1.99-1.93 21H	-[a]	-[a]
**Gal(4)**	102.4; 4.32	69.6; 3.30	71.3; 3.54	67.2; 3.79	72.6; 3.69	62.2; 3.86; 3.40	-[a]	-[a]	-[a]	-[a]	-[a]	-[a]
**Neu5Ac(1)**	-[a]	-[a]	39.1; 2.54; 1.58	67.2; 3.52	50.8; 3.68	71.4; 3.57	67.3; 3.43	70.5; 3.76	61.6; 3.74; 3.51	22.4-22.0; 1.99-1.93 21H	-[a]	-[a]
**Asn**	-[a]	-[a]	-[a]	-[a]	-[a]	-[a]	-[a]	-[a]	-[a]	-[a]	50.9; 3.90	35.0; 2.84; 2.78

[a]Not applicable

ESI TOF-MS: for $C_{105}H_{173}N_9O_{76}$: m/z $[M-3H]^{3-}$; (**2**) calcd: 924.6588; found: 924.6602.

(**3**) calcd: 928.6722; found: 928.6695. (**4**) calcd: 928.6722; found: 928.6702. (**5**) calcd: 926.6655; found: 926.6643.

1.1. NMR spectra

Proton spectrum of **11**, 600 MHz, D_2O.

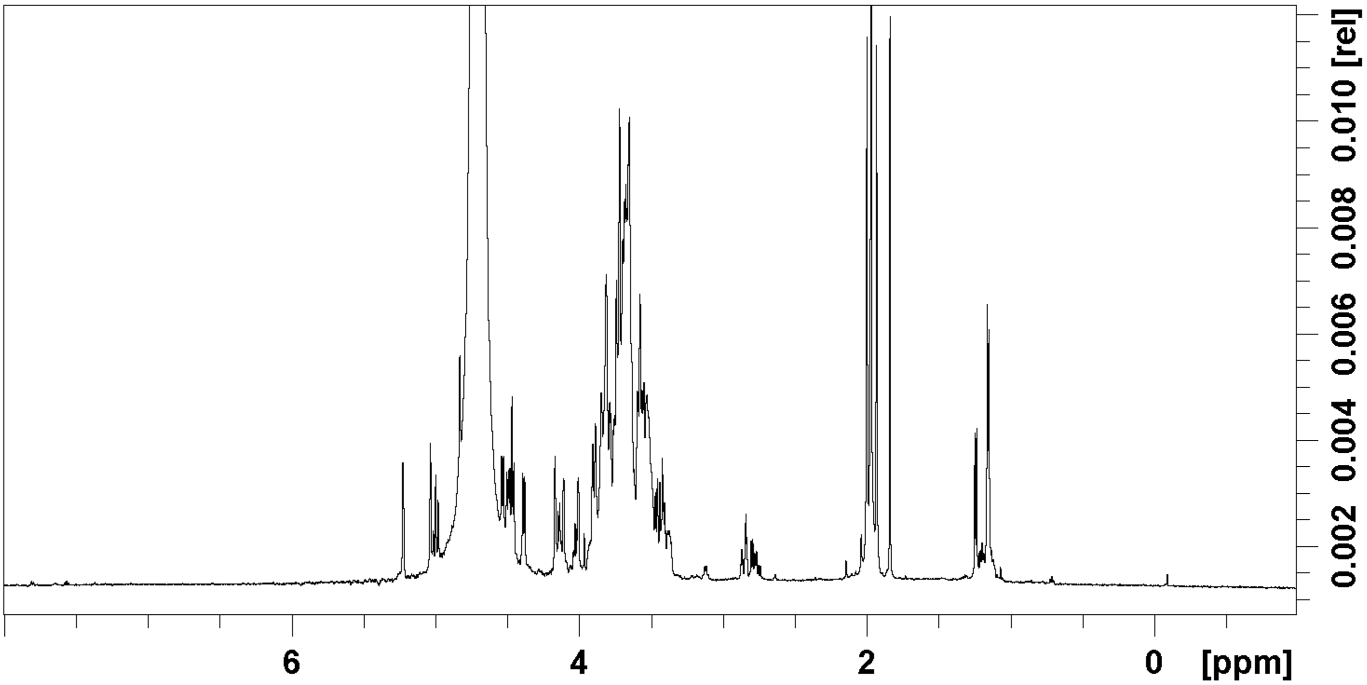

COSY spectrum of **11**, 600 MHz, D_2O.

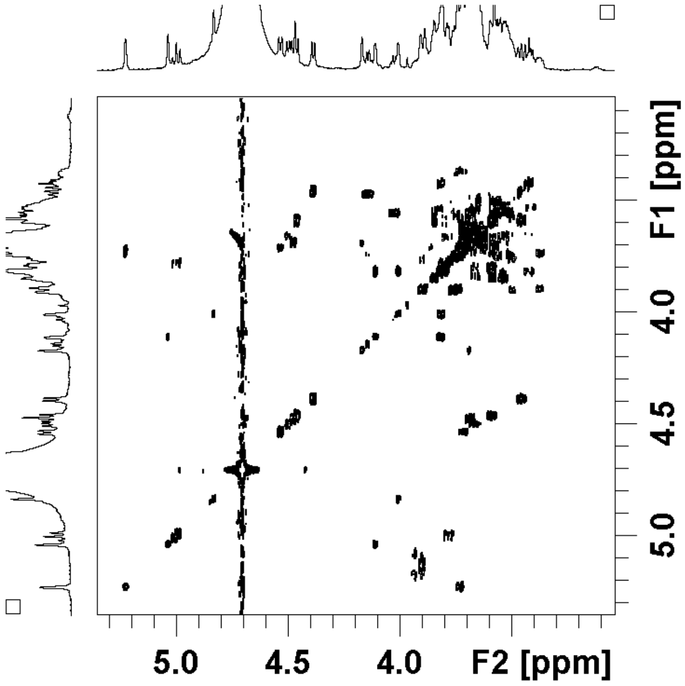

TOCSY spectrum of **11**, 600 MHz, D_2O.

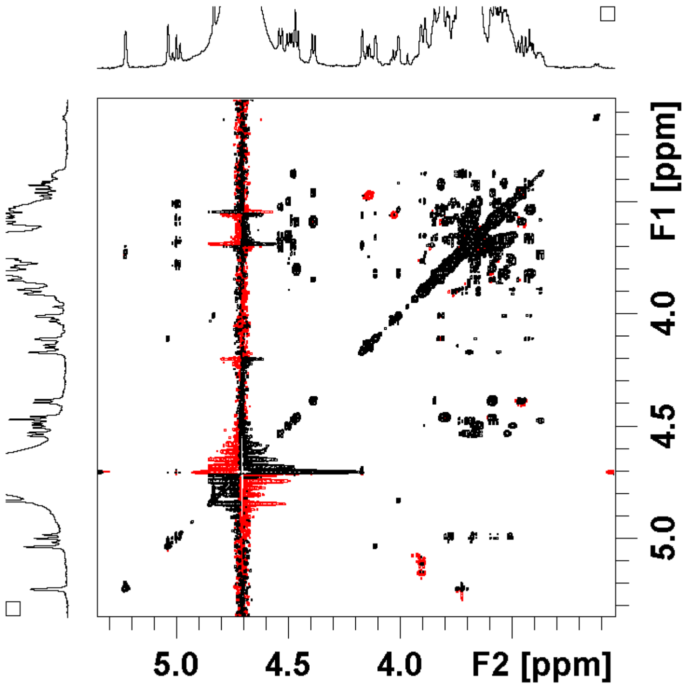

NOESY spectrum of **11**, 600 MHz, D_2O.

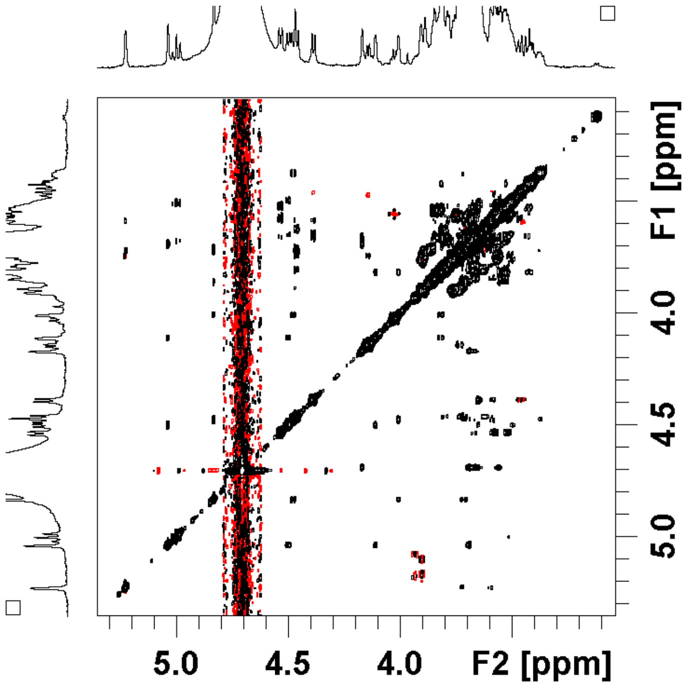

^{1}H-^{13}C-HSQC spectrum of **11**, 600 MHz, D_2O.

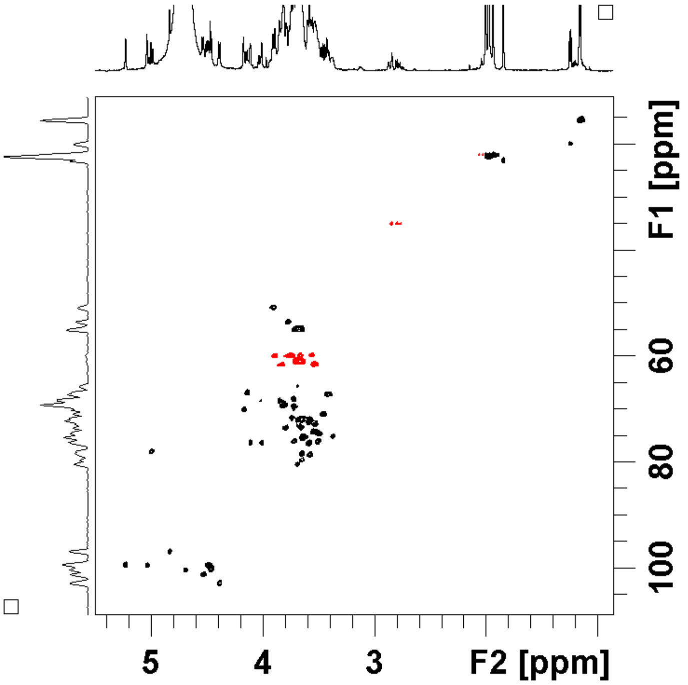

Proton spectrum of **17**, 600 MHz, D_2O.

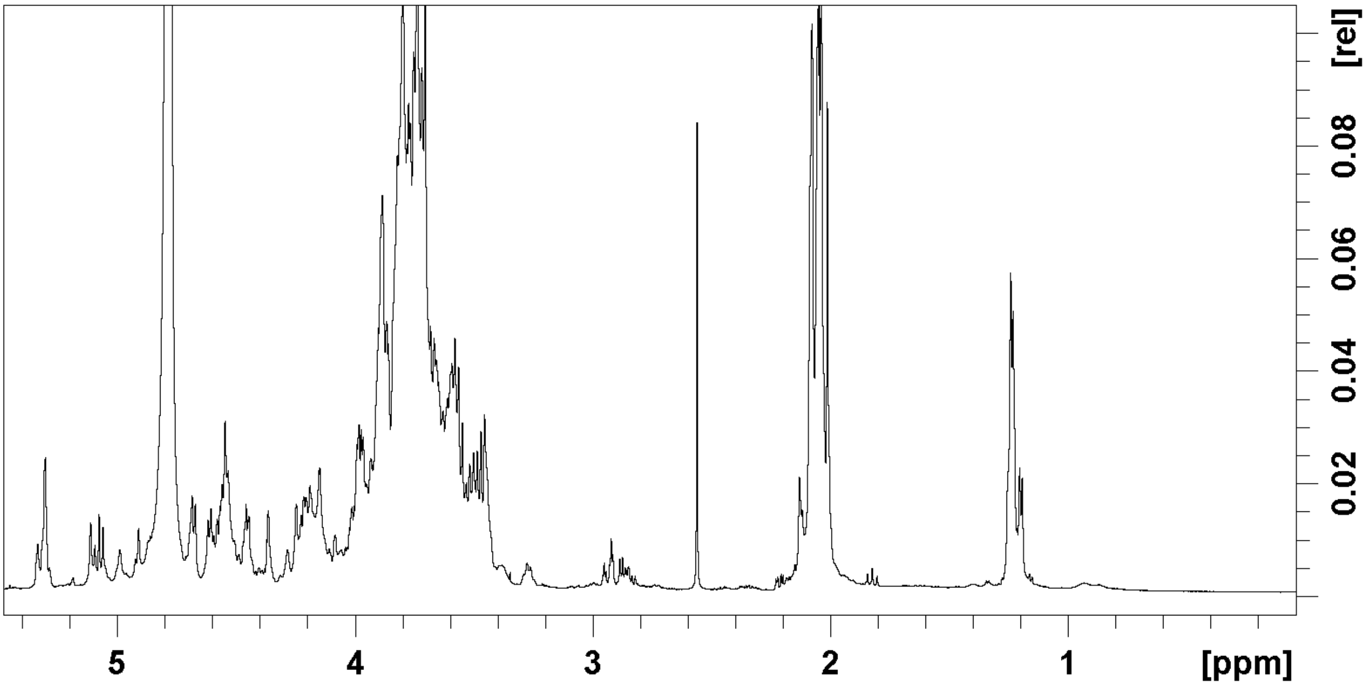

^1H-^{13}C-HSQC spectrum of **17**, 600 MHz, D_2O.

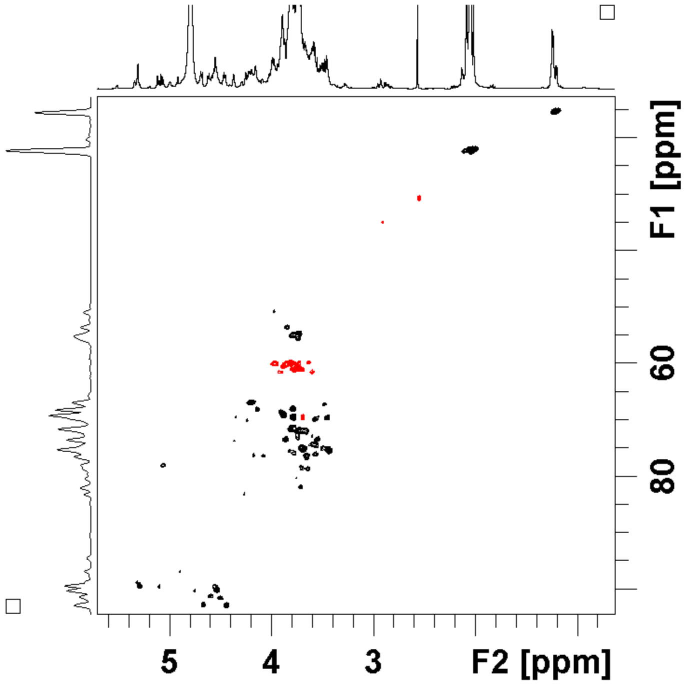

Supplementary Fig. 1. NMR spectra of compound **3**. a) full ^{1}H-NMR spectrum in presence of TSP as internal reference. b) Zoom of the ^{1}H-NMR spectrum. c) ^{1}H-^{13}C HSQC spectrum showing all NMR signals (NS=128). d) ^{1}H-^{13}C HSQC spectrum showing the NMR signals of the labelled residues, exclusively (NS=2).

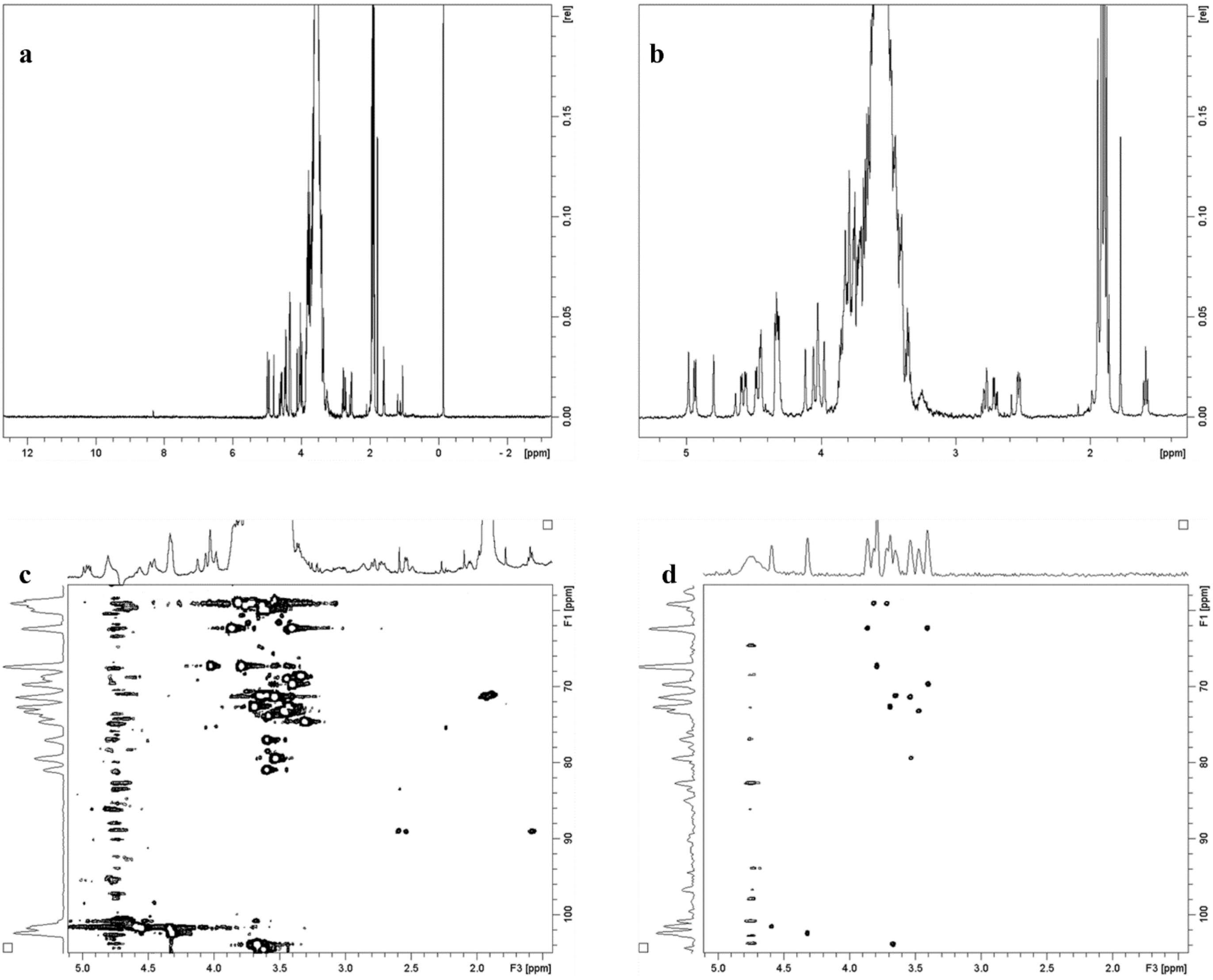

Supplementary Fig. 2. NMR spectra of compound **4**. a) full ^{1}H-NMR spectrum in presence of TSP as internal reference. b) Zoom of the ^{1}H-NMR spectrum. c) ^{1}H-^{13}C HSQC spectrum showing all NMR signals (NS=128). d) ^{1}H-^{13}C HSQC spectrum showing the NMR signals of the labelled residues, exclusively (NS=2).

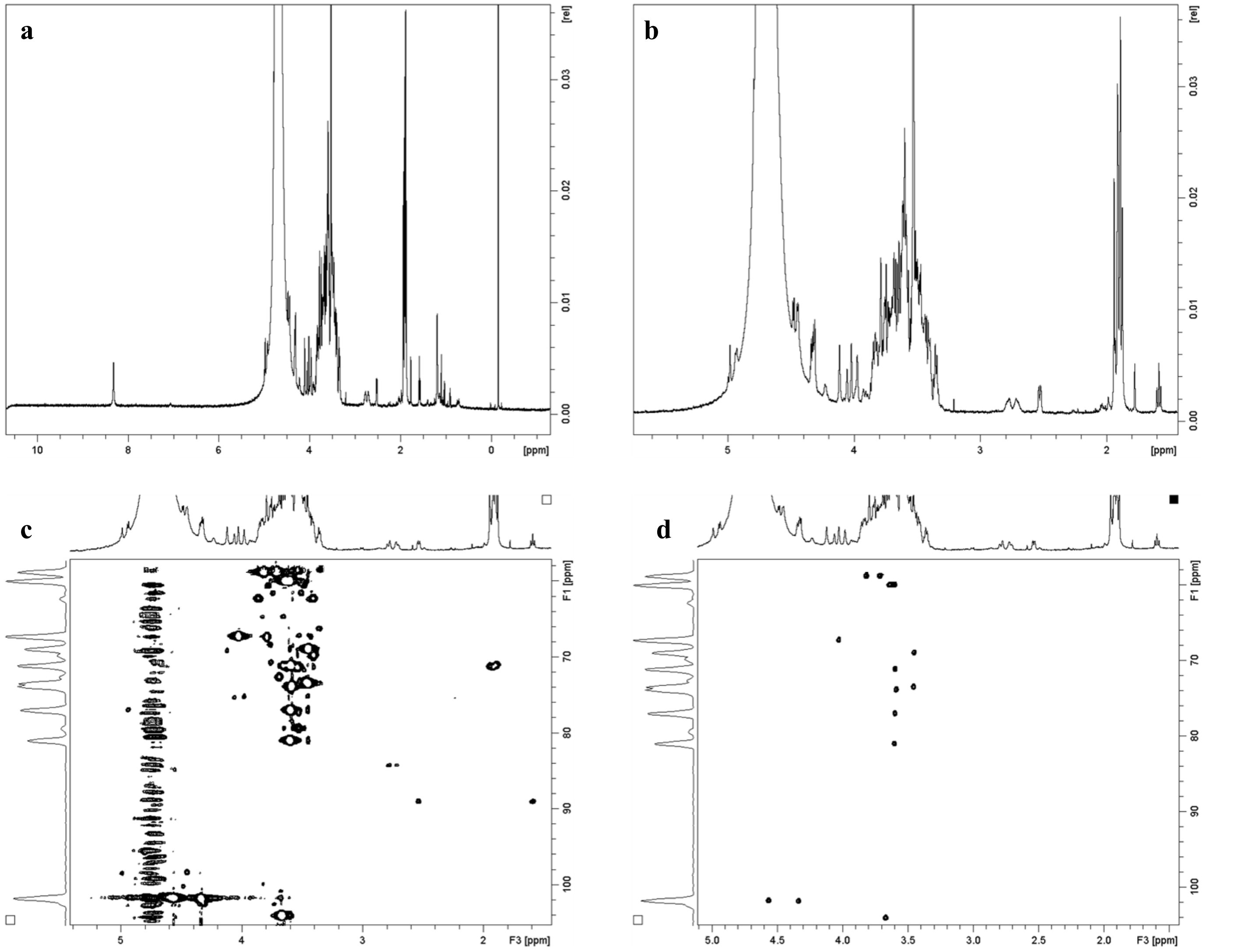

Proton spectrum of **28**, 600 MHz, D_2O.

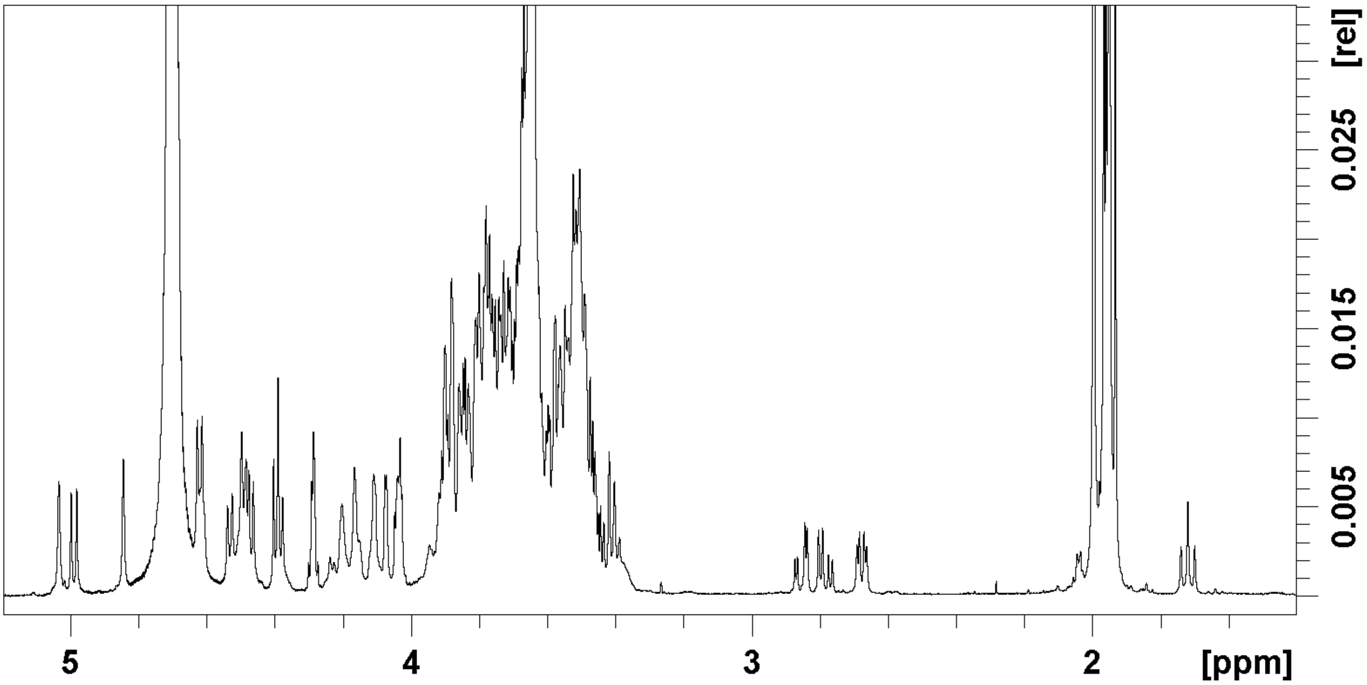

^1H-^{13}C-HSQC spectrum of **28**, 600 MHz, D_2O.

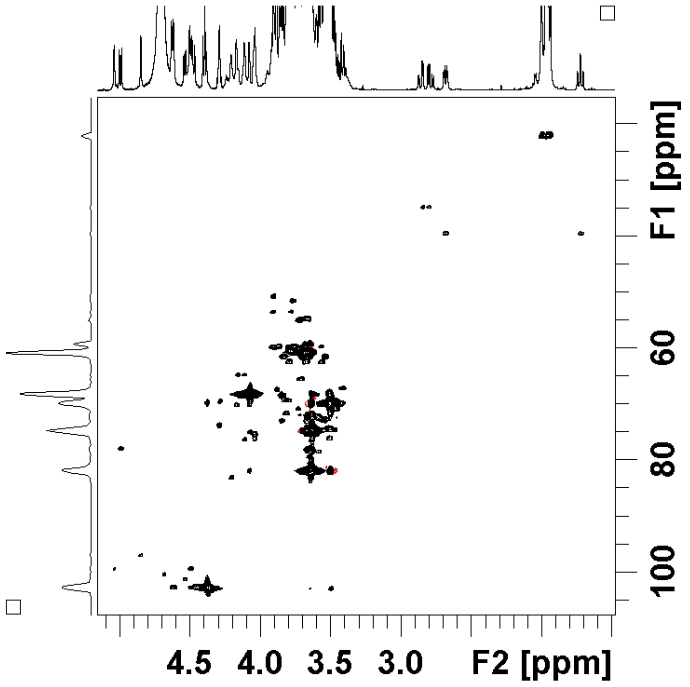

Proton spectrum of **29**, 600 MHz, D_2O.

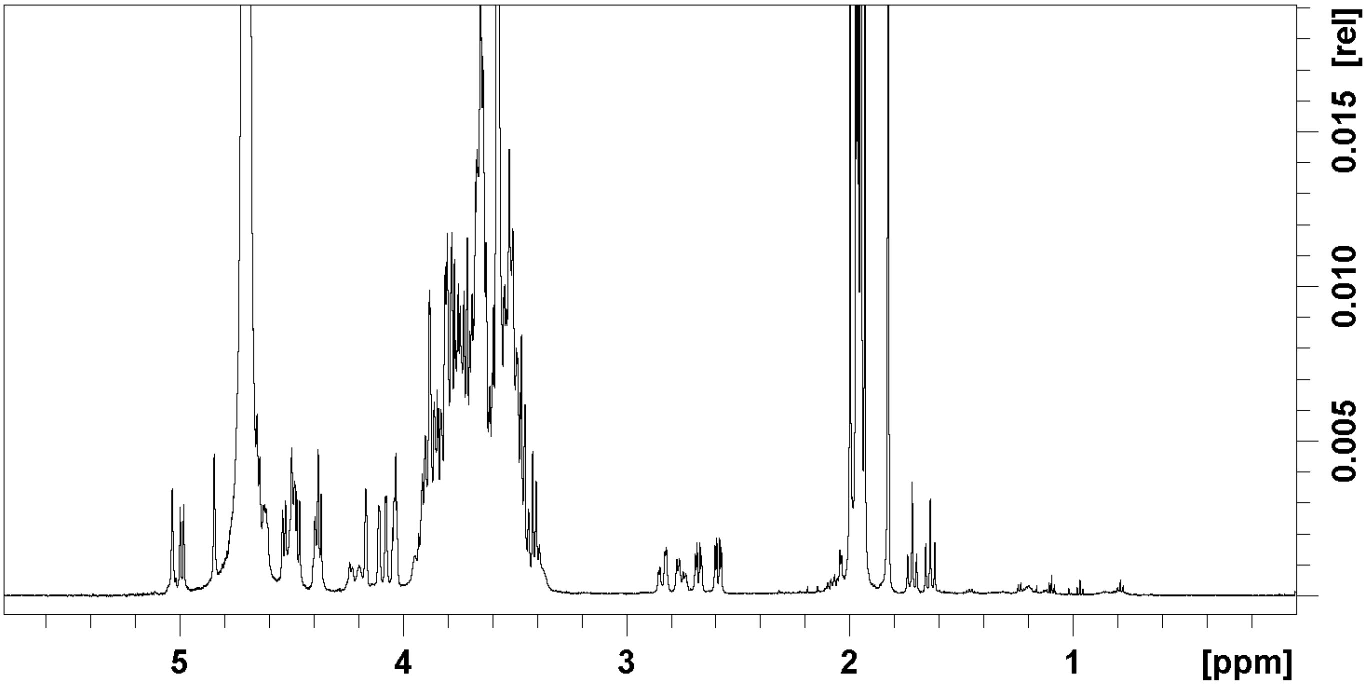

^1H-^{13}C-HSQC spectrum of **29**, 600 MHz, D_2O.

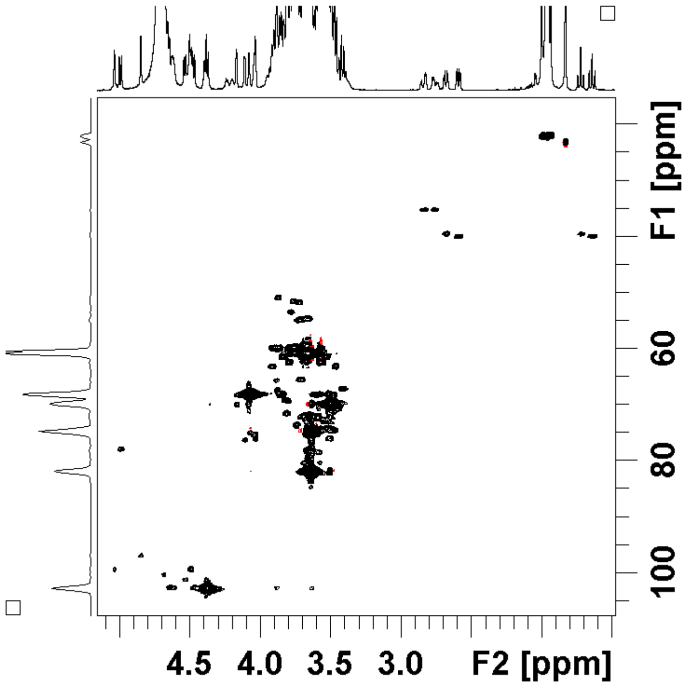

Supplementary Fig. 3. NMR spectra of compound **5**. a) full ^{1}H-NMR spectrum in presence of TSP as internal reference. b) Zoom of the ^{1}H-NMR spectrum. In a and b, 1H-NMR spectra (top) and 1H-{13C} decoupled spectra (bottom). c) ^{1}H-^{13}C HSQC spectrum showing all NMR signals (NS=128). d) ^{1}H-^{13}C HSQC spectrum showing the NMR signals of the labelled residues, exclusively (NS=2).

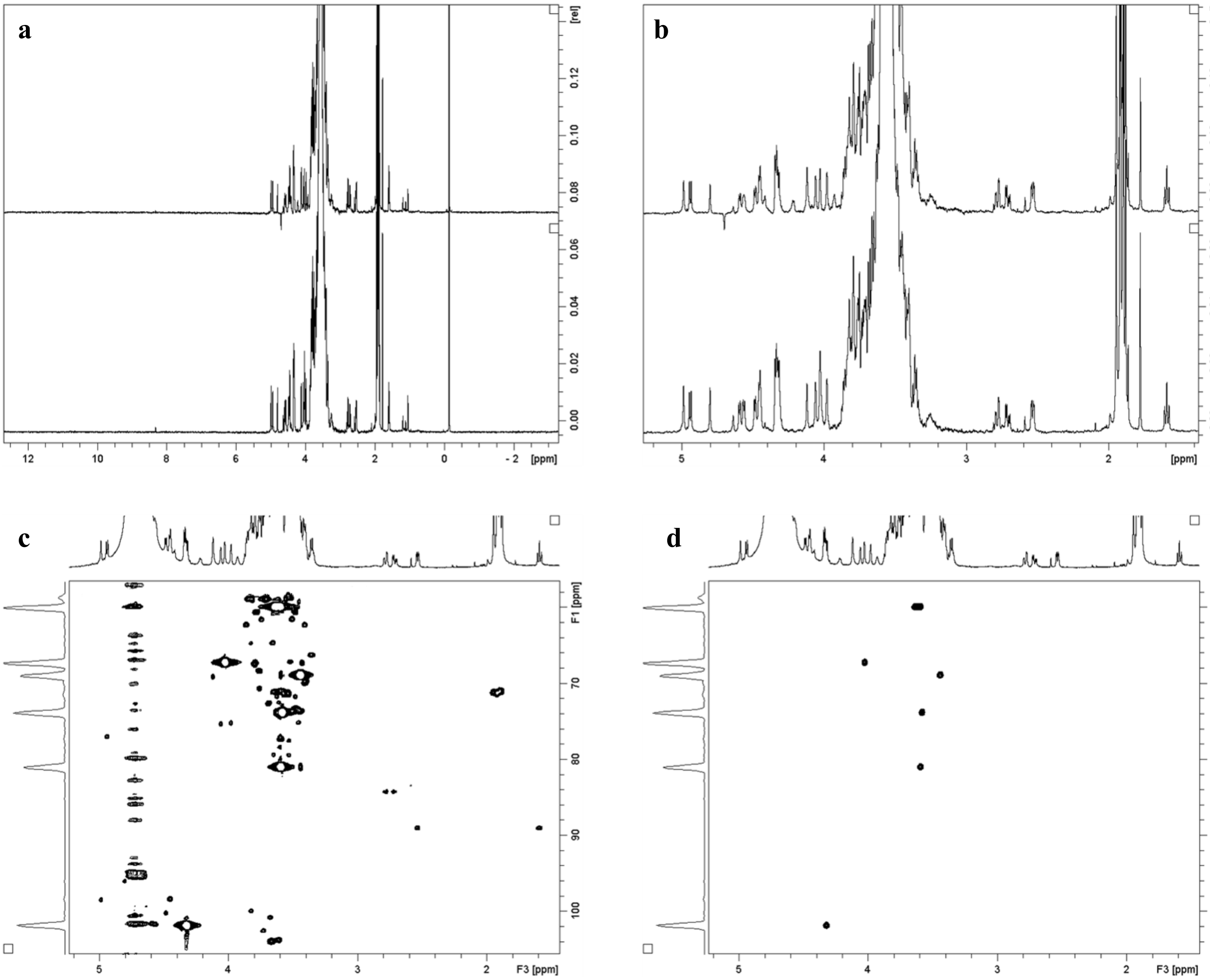

2. Synthesis of CMP-Neu5(CHF_2CONH)

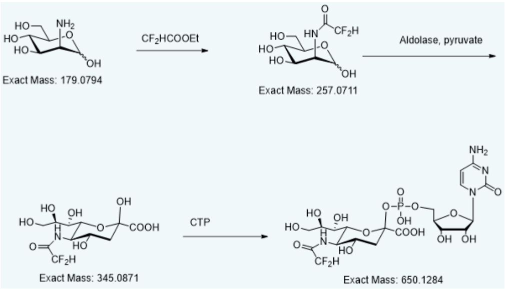

Mannosamine hydrochloride (300 mg, 1.4 mmol) was dissolved in water/MeOH (1:1, 10 mL) and filtered with HCO_3^- resin to release free mannosamine. 1 mL of triethylamine and 1 mlLof ethyl difluoroacetate (7 mmol) were added to the solution, which was then stirred at room temperature for 20 h. The reaction mixture was concentrated *in vacuo*. The residue was purified by silica gel chromatography to give the Man(CHF_2CONH-). The Man(CHF_2CONH-) (1.0 eq, 0.12 mmol, 18 mM) and sodium pyruvate (5.0 eq, 0.6 mmol, 90 mM) were dissolved in MiliQ water in a 50 mL centrifuge tube containing Tris-HCl buffer (100 mM, pH 7.5) and $MgCl_2$ (20 mM). After the addition of sialic acid aldolase, the reaction was incubated at 37 °C for 2 days with shaking. The progress of the reaction was monitored by TLC and LC-MS. Then to this mixture was added CTP (2.0 eq) and the pH was adjusted to 8.5 with 1M NaOH, after which NmCSS and PPA were added. The reaction was incubated at 37 °C for 6 h with shaking. Product formation was monitored by LC-MS. After the generation of CMP-Neu5(CHF_2CONH) was completed, ST6Gal1 and LacNAc acceptor were added to the reaction mixture, which was incubated for 1 day at 37 °C with shaking and product formation was monitored by LC-MS. The reaction mixture was lyophilized and purified by size-exclusion column chromatography using P-2 BioGel eluting with a 50 mM ammonium bicarbonate solution to give **6** as white solid (289 mg, 30%).

General method for the installation of β1,4-galactose using B4GalT1:

To a solution of 10 mM acceptor oligosaccharide in MOPS buffer (MOPS 50 mM, $MnCl_2$ 10 mM, BSA 0.1 wt%, pH 7.3) was added UDP-galactose (1.3 equiv.), CIAP (1 U/μL) and B4GalT1 (10 μg/μmol). The reaction was incubated overnight at 37°C with gentle shaking. Reaction progress was monitored by LC-ESI-QTOF-MS. The reaction mixture was lyophilized if no more starting material was observed. The residue was redissolved in minimal MilliQ water and loaded on a P2-Biogel size exclusion column. Carbohydrate containing fractions were identified by LC-ESI-QTOF-MS, pooled and lyophilized.

General method for the installation of β1,3-N-acetylglucosamine using HP-39:

To a solution of 10 mM acceptor oligosaccharide in MOPS buffer (MOPS 50 mM, $MgCl_2$ 2 mM, BSA 0.1 wt%, pH 7.3) was added UDP-N-acetylglucosamine (1.3 equiv.), CIAP (1 U/μL) and HP-39 (10 μg/μmol). The reaction was incubated overnight at 37°C with gentle shaking. Reaction progress was monitored by LC-ESI-QTOF-MS. The reaction mixture was lyophilized if no more starting material was observed. The residue was redissolved in minimal MilliQ water and loaded on a P2- Biogel size exclusion column. Carbohydrate containing fractions were identified by LC-ESI-QTOF-MS, pooled and lyophilized.

General method for the installation of α2,6-Neu5Ac using ST6Gal1:

To a solution of 10 mM acceptor oligosaccharide in MOPS buffer (MOPS 50 mM, $MgCl_2$ 2 mM, BSA 0.1 wt%, pH 7.3) was added CMP-Neu5Ac or CMP(Neu5(CHF_2CONH-) (1.3 equiv.), CIAP (1 U/μL) and ST6GAL1 (10 μg/μmol). The reaction was incubated overnight at 37°C with gentle shaking. Reaction progress was monitored by LC-ESI-QTOF-MS. The reaction mixture was lyophilized if no more starting

material was observed. The residue was redissolved in minimal MilliQ water and loaded on a P2- Biogel size exclusion column. Carbohydrate containing fractions were identified by LC-ESI-QTOF-MS, pooled and lyophilized.

General method for the installation of α2,3-Neu5Ac using PMST1-M144D:

To a solution of 10 mM acceptor oligosaccharide in MOPS buffer (MOPS 50 mM, $MgCl_2$ 2 mM, BSA 0.1 wt%, pH 7.3) was added CMP-Neu5Ac (1.3 equiv.), CIAP (1 U/μL) and PMST1-M144D (10 μg/μmol). The reaction was incubated overnight at 37°C with gentle shaking. Reaction progress was monitored by LC-ESI-QTOF-MS. The reaction mixture was lyophilized if no more starting material was observed. The residue was redissolved in minimal MilliQ water and loaded on a P2- Biogel size exclusion column. Carbohydrate containing fractions were identified by LC-ESI-QTOF-MS, pooled and lyophilized.

Compound LacNAc-biotin

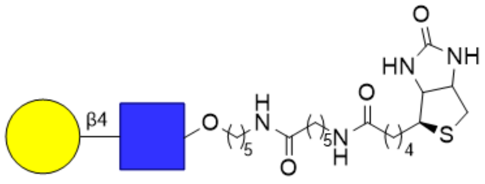

LacNAc-biotin

Compound **LacNAc-azide** (40 mg, 0.081 mmol) was dissolved in a solution of H_2O (4 mL) and 1 M sodium hydroxide (0.8 mL). The reaction was cooled in an ice bath and a 1.0 M solution of trimethylphosphine in tetrahydrofuran (0.40 mL, 0.40 mmol) was added. Reaction was stirred at 0° C for 2 hours, then allowed to warm to room temperature and stir for 2.5 hours. The reaction was followed with LC-ESI-QTOF-MS. The completed reaction mixture was concentrated *in vacuo* and the residue was directly dissolved in DMF/H_2O (2 mL) to which N-hydroxysuccinimde activated Lc-biotin (40.5 mg, 0.090 mmol) was added. Upon completion of the reaction as indicated by LC-ESI-QTOF-MS, the solution was freeze dried and was passed over a Biogel P-2 column for purification to give **LacNAc-biotin** (62.1 mg, 95%) as fluffy, white powder. ^{1}H NMR (600 MHz, D_2O) δ 4.61 (dd, J = 7.8, 4.9 Hz, 1H, H8-Biotin), 4.52 (d, J = 7.4 Hz, 1H, H1-GlcNAc), 4.47 (d, J = 7.7 Hz, 1H, H1- Gal), 4.43 (dd, J = 8.1, 4.6 Hz, 1H, H6-Biotin), 3.99 (1H, d, J = 12.8 Hz, 1H, H6a-GlcNAc), 3.93 (d, J = 3.2 Hz, 1H, H4-Gal), 3.92 – 3.74 (m, 4H, CH_2-linker, H6b-GlcNAc, H6-Gal), 3.74 – 3.69 (m, 4H, H2-GlcNAc, H-3GlcNAc, H-5Gal, H-4GlcNAc), 3.67 (dd, J = 9.8, 3.3 Hz, 1H, H3-Gal), 3.62 – 3.52 (m, 3H, CH_2-linker, H2-Gal, H5-GlcNAc), 3.34 (m, 1H, H5-Biotin), 3.17 (m, 4H, CH_2-linker, CH_2-*Biotin*), 3.00, 2.69 (m, 2H, H9-Biotin), 2.24 (m, 4H, CH_2-Biotin), 2.03 (s, 3H, Ac), 1.77 – 1.28 (m, 18H, CH_2-linker, CH_2-Biotin). ^{13}C NMR (151 MHz, D_2O) δ 179.3, 176.9, 174.8, 167.7 (C=O), 102.7 (C1-Gal), 100.9 (C1-GlcNac), 78.5 (C4-GlcNAc), 75.4 (C5-Gal), 74.8 (H5-GlcNac), 72.6 (C3-Gal, C3-GlcNAc), 71.0 (H2-Gal), 70.3 (OCH_2-linker), 68.6 (C4-Gal), 62.2 (C6-Biotin), 61.0 (C6-Gal), 60.2 (C8-Biotin), 60.1 (C6-GlcNAc), 55.4 (C5-Biotin), 55.1 (C2-GlcNAc), 39.7 (C9-Biotin), 39.2, 39.1 (CH_2-Biotin, CH_2-linker), 35.7, 35.6 (CH_2-Biotin), 28.2, 28.0, 27.9, 27.8, 27.7, 25.5, 25.2, 25.0, 22.5 (CH_2-Biotin, CH_2-linker), 22.2 (CH_3-Ac). HRMS: for $C_{35}H_{61}N_5O_{14}S$: m/z $[M+H]^+$; calcd: 808,4009 ; found: 808,4026.

Compound α2,3-LacNAc-biotin

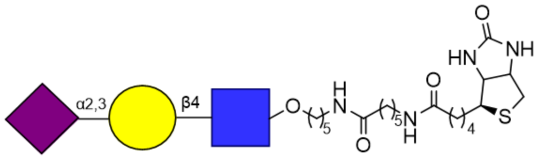

α2,3-LacNAc-biotin

Compound **α2,3-LacNAc-biotin** was prepared from the compound **LacNAc-biotin** (1.0 mg, 1.2 µmol) using the general procedure for the installation of α2,3-Neu5Ac using PMST1-M144D. After P2 purification, **α2,3-LacNAc-biotin** was obtained as a white powder. (1.3 mg, 80%). ^{1}H NMR (600 MHz, D_2O) δ 4.62 (dd, J = 8.2, 5.2 Hz, 1H, H8-Biotin), 4.56 (d, J = 7.6 Hz, 1H, H1-Gal), 4.52 (d, J = 8.1 Hz, 1H, H1-GlcNAc), 4.44 (dd, J = 7.8, 4.4 Hz, 1H, H6-Biotin), 4.13 (1H, dd, J = 9.6, 2.9 Hz, 1H, H3-Gal), 4.00 (dd, J = 12.6, 2.3 Hz, 1H, H6a-GlcNAc), 3.97 (d, J = 3.0 Hz, 1H, H4-Gal), 3.92 – 3.83 (m, 5H, H6b-GlcNAc, H9a-Sia, CH_2-linker, H-8Sia, H5-Sia, 3.76 – 3.67 (m, 7H, H2-GlcNAc, H6-Gal, H4-Sia, H5-GlcNAc, H5-Gal, H4-GlcNAc), 3.76 – 3.63 (m ,2H, H9b-Sia, H6-Sia), 3.62 – 3.56 (m, 4H, H7-Sia, H2-Gal, CH_2-linker, H3-GlcNAc, 3.35 (m, 1H, H5-Biotin), 3.18 (m, 4H, CH_2-linker, CH_2-Biotin),3.01 (dd, J = 12.9, 4.9 Hz, 1H, H9a-Biotin), 3.78 (m, 2H, H9b-Biotin, H3eq-Sia), 2.25 (m, 4H, CH_2-Biotin), 2.04 (s, 6H, 2 x Ac), 1.81 (t, J = 12.1 Hz, 1H, H3ax-Sia), 1.75 – 1.28 (m, 18H, CH_2-linker, CH_2-Biotin). ^{13}C NMR (151 MHz, D_2O) δ 176.7 (2x), 175.1, 174.3, 173.9, 165.0 (C=O), 102.5 (C1-Gal), 101.0 (C1-GlcNac), 99.7, 78.3 (C4-GlcNAc), 75.4 (C3-Gal), 75.2 (H5-Gal), 74.7 (C3-GlcNAc), 72.9 (H6-Sia), 72.4 (C5-GlcNAc), 71.7 (H8-Sia), 70.3 (OCH_2-linker), 69.4 (C2-Gal), 68.3 (C4-Sia), 68.0 (C7-Sia), 67.4 (C4-Gal), 62.5 (C9-Sia), 62.0 (C6-Biotin), 60.9 (C6-Gal), 60.2 (C8-Biotin), 60.1 (C6-GlcNAc), 55.4 (C5-Biotin), 55.1 (C2-GlcNAc), 51.6 (C5-Sia), 39.7, 39.6 (C9-Biotin, H3-Sia), 39.2, 39.1 (CH_2-Biotin, CH_2-linker), 35.6, 35.5 (CH_2-Biotin), 28.2, 28.0, 27.9, 27.8, 27.7, 25.5, 25.2, 25.0, 22.5 (CH_2-

Biotin, CH_2-linker), 22.2, 22.0 (CH_3-Ac). HRMS: for $C_{46}H_{78}N_6O_{22}S$: m/z $[M-H]^-$; calcd: 1097,4817; found: 1097,4825.

Compound α2,6(Neu5(CHF_2CONH-)-LacNAc-biotin (6)

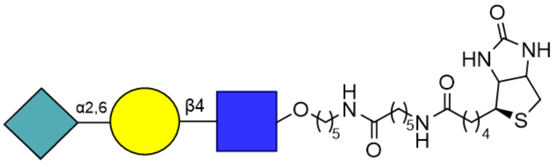

α2,6(Neu5(CHF_2CONH-)-LacNAc-biotin

Compound **α2,6(Neu5(CHF_2CONH-)-LacNAc-biotin** was prepared from the compound **LacNAc-biotin** (1.0 mg, 1.2 µmol) using the general procedure for the installation of α2,6-Neu5Ac using CMP-(Neu5(CHF_2CONH-) and ST6GAL1. After P2 purification, **α2,6(Neu5(CHF_2CONH-)-LacNAc-biotin** was obtained as a white powder (1.2 mg, 88%). ^1H NMR (600 MHz, D_2O) δ 6.16 (t, J_{H-F} = 53.9 Hz, 1H, CHF_2), 4.62 (m, 1H, H8-Biotin), 4.56 (d, J = 7.4 Hz, 1H, H1-GlcNAc), 4.46 – 4.41 (m, 2H, H1-Gal, H6-Biotin), 4.03 – 3.97 (m, 2H, H6a-GlcNAc, H6a-Gal), 3.96 – 3.86 (m, 6H, H5-Sia, H6b-GlcNAc, H4-Gal, H9a-Sia, CH_2-linker, H8-Sia), 3.85 – 3.81 (m, 2H,H6b-GlcNAc, H5-Gal), 3.76 – 3.70 (m, 3H, H2-GlcNAc, H4-Sia, H3-Gal), 3.69 – 3.59 (m, 6H, H9b-Sia, CH_2-linker, H5-GlcNAc, H4-GlcNAc, H3-GlcNAc, H6-Sia), 3.57 – 3.52 (m, 3H, H6b-Gal, H7-Sia, H2-Gal), 3.34 (m, 1H, H5-Biotin), 3.18 (m, 4H, CH_2-linker, CH_2-Biotin), 3.00 (dd, J = 12.9, 4.9 Hz, 1H, H9a-Biotin), 3.79 (d, J = 12.9 Hz, 1H, H9b-Biotin), 2.69 (dd, J = 13.0, 5.2 Hz, 1H, H3eq-Sia), 2.25 (m, 4H, CH_2-Biotin), 2.07 (s, 3H, Ac), 1.77 – 1.28 (m, 19H, H3ax-Sia, CH_2-linker, CH_2-Biotin). ^{13}C NMR (151 MHz, D_2O) δ 176.7, 174.6, 174.5, 173.3, 165.2 (C=O), 108.2 (CHF_2), 103.4 (C1-Gal), 100.9 (C1-GlcNac), 80.9 (C4-GlcNAc), 74.5 (C5-GlcNAc), 73.8 (C5-Gal), 72.5, 72.4 (H6-Sia, C3-GlcNAc, C3-Gal), 71.9 (C8-Sia), 70.7 (C2-Gal), 70.3 (OCH_2-linker), 68.4 (C7-Sia, C4-Gal), 68.0 (C4-Sia), 63.5 (C6-Gal), 62.6 (C9-Sia), 62.1 (C6-Biotin), 60.3 (C6-GlcNAc), 60.2 (C8-Biotin), 55.4 (C5-Biotin), 55.0 (C2-GlcNAc), 51.7 (C5-Sia), 40.1 (C3-Sia), 39.7 (C9-Biotin), 39.2, 39.1 (CH_2-Biotin, CH_2-linker), 35.6 (CH_2-Biotin), 28.2, 28.0, 27.9, 27.8, 27.7, 25.5, 25.2, 25.0, 22.5 (CH_2-Biotin, CH_2-linker), 22.2 (CH_3-Ac). HRMS: for $C_{46}H_{76}F_2N_6O_{22}S$: m/z $[M+Na]^+$; calcd: 1157,4593; found: 1157,4605.

Compound α2,6-LacNAc-biotin (7)

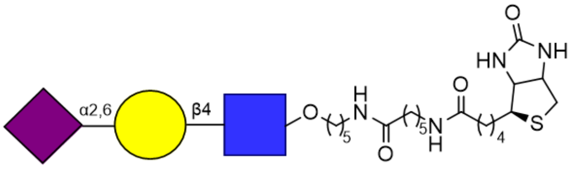

α2,6-LacNAc-biotin

Compound **α2,6-LacNAc-biotin** was prepared from the compound **LacNAc-biotin** (1.0 mg, 1.2 µmol) using the general procedure for the installation of α2,6-Neu5Ac using ST6GAL1. After P2 purification, **α2,6-LacNAc-biotin** was obtained as a white powder (1.4 mg, 86%). ^1H NMR (600 MHz, D_2O) δ 4.62 (dd, J = 8.3, 5.5 Hz, 1H, H8-Biotin), 4.56 (d, J = 7.6 Hz, 1H, H1-GlcNAc), 4.47 – 4.42 (m, 2H, H1-Gal, H6-Biotin), 4.02 – 3.97 (m, 2H, H6a-GlcNAc, H6a-Gal), 3.93 (d, J = 3.7 Hz, 1H, H4-Gal), 3.92 – 3.87 (m, 3H, H9a-Sia, CH_2-linker, H8-Sia), 3.85 – 3.78 (m, 3H, H5-Sia, H6b-GlcNAc, H5-Gal), 3.76 – 3.70 (m, 2H, H2-GlcNAc, H3-Gal), 3.70 – 3.59 (m, 7H, H9b-Sia, CH_2-linker, H5-GlcNAc, H4-GlcNAc, H3-GlcNAc, H4-Sia, H6-Sia), 3.59 – 3.52 (m, 3H, H6b-Gal, H7-Sia, H2-Gal), 3.35 (m, 1H, H5-Biotin), 3.19 (m, 4H, CH_2-linker, CH_2-Biotin), 3.00 (dd, J = 13.2, 5.5 Hz, 1H, H9a-Biotin), 3.78 (d, J = 13.2 Hz, 1H, H9b-Biotin), 2.67 (dd, J = 12.5, 5.0 Hz, 1H, H3eq-Sia), 2.16 (m, 4H, CH_2-Biotin), 1.97, 1.95 (s, 6H, 2 x Ac), 1.76 – 1.28 (m, 19H, H3ax-Sia, CH_2-linker, CH_2-Biotin). ^{13}C NMR (151 MHz, D_2O) δ 176.7, 174.6, 174.3, 173.9, 160.4 (C=O), 103.5 (C1-Gal), 100.9 (C1-GlcNac), 80.8 (C4-GlcNAc), 74.5 (C5-GlcNAc), 73.7 (C5-Gal), 72.4 (H6-Sia, C3-GlcNAc, C3-Gal), 71.7 (C8-Sia), 70.6 (C2-Gal), 70.3 (OCH_2-linker), 68.4 (C7-Sia, C4-Gal), 68.2 (C4-Sia), 63.3 (C6-Gal), 62.6 (C9-Sia), 62.2 (C6-Biotin), 60.4 (C6-GlcNAc), 60.3 (C8-Biotin), 55.4 (C5-Biotin), 55.0 (C2-GlcNAc), 51.7

(C5-Sia), 40.1 (C3-Sia), 39.7 (C9-Biotin), 39.2, 39.1 (CH_2-Biotin, CH_2-linker), 35.7, 35.6 (CH_2-Biotin), 28.2, 28.0, 27.9, 27.8, 27.7, 25.5, 25.2, 25.0, 22.5 (CH_2-Biotin, CH_2-linker), 22.3, 22.0 (CH_3-Ac).

Compound di-LacNAc-biotin

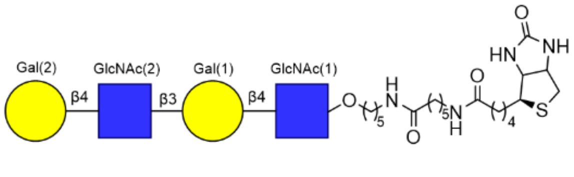

di-LacNAc-biotin

Compound **di-LacNAc-biotin** was prepared from the compound **LacNAc-biotin** (7.8 mg, 9.6 µmol) using the general procedure for the installation of **β1,3-N-acetylglucosamine using HP-39**. After full conversion the reaction mixture was centrifuged over a Nanosep Omega ultrafiltration device (10 kDa MWCO) to remove proteins. Filtrate was lyophilized, followed by the general procedure for the installation of **β1,4-galactose using B4GalT1.** After P2 purification **di-LacNAc-biotin** was obtained as a white powder (8.8 mg, 78%). ^{1}H NMR (600 MHz, D_2O) δ 4.71 (d, J = 8.5 Hz, 1H, H1-GlcNAc(2)), 4.61 (dd, J = 7.8, 4.5 Hz, 1H, H8-Biotin), 4.52 (d, J = 7.8 Hz, 1H, H1-GlcNAc(1)), 4.48 (m, 2H, H1-Gal(1,2)), 4.43 (dd, J = 8.2, 4.5 Hz, 1H, H6-Biotin), 4.17 (d, J = 3.4 Hz, 1H, H4-Gal(1)), 4.01 – 3.88 (m, 5H, H6a-GlcNAc(1,2), H4-Gal(2), CH_2-linker), 3.88 – 3.79 (m, 3H, H6b-GlcNAc(1,2), H2-GlcNAc(2)), 3.79 – 3.65 (m, 13H, H6-Gal(1,2), H2-GlcNAc(1), H3-GlcNAc(1,2), H3-Gal(1,2), H5-Gal(1,2), H4-Glc(1,2)), 3.62 – 3.53 (m, 4H, H2-Gal(1,2), H5-Glc(1,2)), 3.34 (m, 1H, H5-Biotin), 3.18 (m, 4H, CH_2-linker, CH_2-Biotin), 3.00 (dd, J = 12.9, 5.6 Hz, 1H, H9a-Biotin), 2.79 (d, J = 12.9 Hz, 1H, H9b-Biotin), 2.25 (m, 4H, CH_2-Biotin), 2.04 (2s, 6H, 2 x Ac), 1.76 – 1.28 (m, 18H, CH_2-linker, CH_2-Biotin). ^{13}C NMR (151 MHz, D_2O) δ 176.7, 176.6, 174.9, 174.3, 165.3 (C=O), 102.9, 102.7 (C1-Gal(1,2)), 102.8 (C1-GlcNAc(2)), 101.0 (C1-GlcNAc(1)), 82.0 (C3-Gal(1)), 78.4 (C4-Glc(1)), 78.1 (C4-Glc(2)), 75.3 (H5-Gal(1,2)), 74.8 (H5-Glc(1,2)), 72.5 (H3-Gal(2), H3-Glc(1)), 72.2 (H3-Glc(2)), 71.0 (H2-Gal(2)), 70.3 (OCH_2-linker), 70.0 (H2-Gal(1)), 68.6 (H4-Gal(2)), 68.3 (H4-Gal(1)), 62.0 (C6-Biotin), 61.0, 60.9 (C6-Gal(1,2)), 60.2 (C8-Biotin), 60.0, 59.9 (C6-GlcNAc(1,2)), 55.4 (C5-Biotin), 55.2, 55.1 (C2-GlcNAc(1,2)), 39.7 (C9-Biotin), 39.2, 39.1 (CH_2-Biotin, CH_2-linker), 35.6, 35.4 (CH_2-Biotin), 28.2, 28.0, 27.9, 27.8, 27.6, 25.5, 25.2, 25.0, 22.5 (CH_2-Biotin, CH_2-linker), 22.2 (2x CH_3-Ac).

Compound α2,6-di-LacNAc-biotin (8)

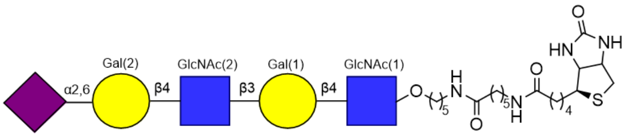

α2,6-di-LacNAc-biotin

Compound **α2,6-di-LacNAc-biotin** was prepared from the compound **di-LacNAc-biotin** (1.0 mg, 0.85 µmol) using the general procedure for the installation of α2,6-Neu5Ac using ST6GAL1. After P2 purification, **α2,6-di-LacNAc-biotin** was obtained as a white powder (1.1 mg, 88%). ^{1}H NMR (600 MHz, D_2O) δ 4.74 (m, 1H, H1-GlcNAc(2)), 4.62 (m, 1H, H8-Biotin), 4.52 (d, J = 7.8 Hz, 1H, H1-GlcNAc(1)), 4.47 (m, 2H, H1-Gal(1,2)), 4.44 (m, 1H, H6-Biotin), 4.17 (m, 1H, H4-Gal(1)), 4.02 – 3.93 (m, 4H, H6a-GlcNAc(1,2), H6a-Gal(2), H4-Gal(2)), 3.92 – 3.87 (m, 4H, H9a-Sia, CH_2-linker, H8-Sia), 3.87 – 3.63 (m, 17H, C5-Sia, H2-GlcNAc(1,2), H6b-GlcNAc(1,2), H6-Gal(1), H9-Sia, H4-Sia, H5-Gal(2), H6-Sia, H3-Gal(1,2), H3-GlcNAc(1,2), H4-GlcNAc(1,2)), 3.63 – 3.52 (m, 7H, H6b-Gal(2), H7-Sia, H2-Gal(1,2), H5-GlcNAc(1,2), H5-Gal(1)), 3.35 (m, H5-Biotin), 3.19 (m, 4H, CH_2-linker, CH_2-Biotin), 3.00 (dd, J = 12.9, 4.5 Hz, 1H, H9a-Biotin), 2.79 (d, J = 12.9 Hz, 1H, H9b-Biotin), 2.68 (dd, J = 12.3, 4.5 Hz, 1H, H3eq-Sia), 2.25 (m, 4H, CH_2-Biotin), 2.07, 2.04 (2s, 9H, 3 x Ac), 1.76 – 1.28 (m, 19H, H3ax-Sia, CH_2-linker, CH_2-Biotin). ^{13}C NMR (151 MHz, D_2O) δ 176.6, 174.9, 174.5 (C=O), 103.2, 102.7 (C1-Gal(1,2)), 102.5 (C1-GlcNAc(2)), 101.0 (C1-GlcNAc(1)), 82.1 (C3-Gal(1)), 80.5 (C4-Glc(1)), 78.5 (C4-(Glc2)), 75.0, 74.6 (C5-Gal(1), C5-GlcNAc(1,2)), 73.7 (C5-Gal(2)), 72.5, 72.3 (C3-Gal(2), C3-

GlcNAc(1,2), C6-Sia), 71.7 (C8-Sia), 70.7 (C2-Gal(2)), 70.3 (CH_2-linker), 68.3 (C4-Gal(1,2), C4-Sia, C2-Gal(1), C7-Sia), 63.4 (C6-Gal(2)), 62.7 (C9-Sia), 62.1 (C6-Biotin), 61.0 (C6-Gal(1)), 60.2 (C8-Biotin), 60.1 (C6-GlcNAc(1,2)), 55.6 (C5-Biotin), 55.0 (C2-GlcNAc(1), 54.8 C2-GlcNAc(2)), 51.8 (C5-Sia), 40.0 (C3-Sia), 39.7 (C9-Biotin), 39.1 (CH_2-Biotin, CH_2-linker), 35.7 (CH_2-Biotin), 28.2, 28.0, 27.9, 27.8, 27.6, 25.5, 25.2, 25.0, 22.5 (CH_2-Biotin, CH_2-linker), 22.0 (CH_3-Ac).

Compound α2,6(Neu5(CHF_2CONH-)-di-LacNAc-biotin (10)

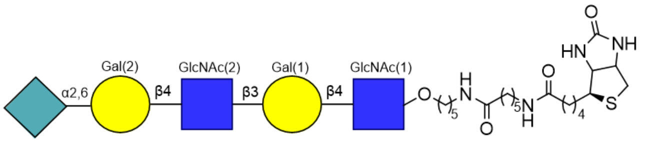

α2,6(Neu5(CHF_2CONH-)-di-LacNAc-biotin

Compound **α2,6(Neu5(CHF_2CONH-)-di-LacNAc-biotin** was prepared from compound **di-LacNAc-biotin** (1.0 mg, 0.85 μmol) using the general procedure for the installation of α2,6-Neu5Ac using CMP-(Neu5(CHF_2CONH-) and ST6GAL1. After P2 purification, **α2,6(Neu5(CHF_2CONH-)-di-LacNAc-biotin** was obtained as a white powder (1.2 mg, 92%). ^{1}H NMR (600 MHz, D_2O) δ 6.17 (t, J_{H-F} = 53.7 Hz, 1H, CHF_2), 4.75 (d, J = 7.8 Hz, 1H, H1-GlcNAc(2)), 4.62 (m, 1H, H8-Biotin), 4.52 (d, J = 7.8 Hz, 1H, H1-GlcNAc(1)), 4.47 (m, 2H, H1-Gal(1,2)), 4.44 (dd, J = 8.0, 4.7 Hz, 1H, H6-Biotin), 4.16 (d, J = 3.3 Hz, 1H, H4-Gal(1)), 4.04 - 3.94 (m, 5H, H5-Sia, H6a-GlcNAc(1,2), H6a-Gal(2), H4-Gal(2)), 3.93 - 3.67 (m, 19H, H9a-Sia, CH_2-linker, H8-Sia, H2-GlcNAc(1,2), H6b-GlcNAc(1,2), H6-Gal(1), H4-Sia, H5-Gal(2), H6-Sia, H3-Gal(1,2), H3-GlcNAc(1,2), H4-GlcNAc(1,2)), 3.66 – 3.53 (m, 8H, H9b-Sia, H6b-Gal(2), H2-Gal(1,2), H7-Sia, H5-Gal1, H5-GlcNAc(1,2)), 3.34 (m, H5-Biotin), 3.18 (m, 4H, CH_2-linker, CH_2-Biotin), 3.00 (dd, J = 13.1, 4.9 Hz, 1H, H9a-Biotin), 2.79 (d, J = 13.1 Hz, 1H, H9b-Biotin), 2.69 (dd, J = 13.6, 4.9 Hz, 1H, H3eq-Sia), 2.25 (m, 4H, CH_2-Biotin), 2.08, 2.03 (2s, 6H, 2 x Ac), 1.78 – 1.28 (m, 19H, H3ax-Sia, CH_2-linker, CH_2-Biotin). ^{13}C NMR (151 MHz, D_2O) δ 176.6, 174.8, 174.3, 173.3, 165.3 (C=O), 108.2 (CHF_2), 103.5, 102.9 (C1-Gal(1,2)), 102.6 (C1-GlcNAc(2)), 101.0 (C1-GlcNAc(1)), 82.1 (C3-Gal(1)), 80.5 (C4-Glc(1)), 78.5(C4-Glc(2)), 74.9, 74.8, 74.3 (C5-Gal(1), C5-GlcNAc(1,2)), 73.7 (C5-Gal2), 72.5, 72.4, 72.2, 71.9, 71.8 (C3-Gal(2), C3-GlcNAc(1,2), C6-Sia, C8-Sia), 70.7 (C2-Gal(2)), 70.3 (CH_2-linker), 68.4, 68.3 (2x) (C2-Gal(1), C7-Sia, C4-Gal(1,2)), 68.0 (C4-Sia), 63.4 (C6-Gal(2)), 62.6 (C9-Sia), 62.1 (C6-Biotin), 61.0 (C6-Gal(1)), 60.2 (C8-Biotin), 61.0 60.1 (C6-GlcNAc(1,2)), 55.6 (C5-Biotin), 55.0 (C2-GlcNAc(1), 54.8 C2-GlcNAc(2)), 51.9 (C5-Sia), 40.0 (C3-Sia), 39.6 (C9-Biotin), 39.0 (CH_2-Biotin, CH_2-linker), 35.4, 35.3 (CH_2-Biotin), 28.2, 28.0, 27.9, 27.8, 27.6, 25.4, 25.2, 25.0, 22.5 (CH_2-Biotin, CH_2-linker), 22.3, 22.2 (CH_3-Ac). HRMS: for $C_{60}H_{99}F_2N_7O_{32}S$: m/z $[M-2H]^{2-}$; calcd: 748,7939; found: 748,7948.

Compound **tri-LacNAc-biotin**

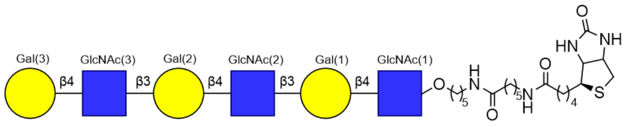

tri-LacNAc-biotin

Compound **tri-LacNAc-biotin** was prepared from compound **di-LacNAc-biotin** (5.0 mg, 4.3 μmol) using the general procedure for the installation of **β1,3-N-acetylglucosamine using HP-39**. After full conversion the reaction mixture was centrifuged over a Nanosep Omega ultrafiltration device (10 kDa MWCO) to remove proteins. Filtrate was lyophilized, followed by the general procedure for the installation of **β1,4-galactose using B4GalT1.** After P2 purification **tri-LacNAc-biotin** was obtained as a white powder (4.6 mg, 70%). ^{1}H NMR (600 MHz, D_2O) δ 4.71 (m, 1H, H1-GlcNAc(2,3)), 4.62 (m, 1H, H8-Biotin), 4.52 (d, J = 7.7 Hz, 1H, H1-GlcNAc(1)), 4.50 – 4.45 (m, 3H, H1-Gal(1,2,3)), 4.43 (dd, J = 8.0, 4.8 Hz, 1H, H6-Biotin), 4.16 (m, 2H, H4-Gal(1,2)), 4.01 – 3.88 (m, 6H, H6a-GlcNAc(1,2,3), H4-Gal(3), CH_2-linker), 3.86 – 3.80 (m, 5H, H6b-GlcNAc(1,2,3), H2-GlcNAc(2,3)), 3.80

– 3.66 (m, 19H, H6-Gal(1,2,3), H2-GlcNAc(1), H3-GlcNAc(1,2,3), H5-Gal(1,2,3), H4-Glc(1,2,3), H3-Gal(1,2,3)), 3.60 – 3.54 (m, 6H, H2-Gal(1,2,3), H5-Glc(1,2,3)), 3.35 (m, 1H, H5-Biotin), 3.18 (m, 4H, CH_2-linker, CH_2-Biotin), 3.00 (dd, J = 13.3, 5.3 Hz, 1H, H9a-Biotin), 2.79 (d, J = 13.3 Hz, 1H, H9b-Biotin), 2.25 (m, 4H, CH_2-Biotin), 2.04 (m, 9H, 3 x Ac), 1.76 – 1.28 (m, 18H, CH_2-linker, CH_2-Biotin). ^{13}C NMR (151 MHz, D_2O) δ 176.7, 176.6, 174.9, 174.3, 165.3 (C=O), 102.9, 102.8, 102.7 (C1-Gal(1,2,3)), 102.6 (C1-GlcNAc(2,3)), 101.0 (C1-GlcNAc(1)), 82.0 (C3-Gal(1,2)), 78.5 (C4-GlcNAc(1)), 78.1 (C4-GlcNAc(2,3)), 75.3, 74.9, 74.7, 74.5 (C5-Gal(1,2,3), C5-GlcNAc(1,2,3)), 72.5, 72.4 (C3-GlcNAc(1), C3-Gal(3)), 72.2 C3-GlcNAc(2,3)), 71.0 (C2-Gal(3)), 70.3 (OCH_2-linker), 69.9 (C2-Gal(1,2)), 68.5 (C4-Gal(3)), 68.3 (C4-Gal(1,2)), 62.1 (C6-Biotin), 61.0, 60.9 (C6-Gal(1,2,3)), 60.2 (C8-Biotin), 60.1, 59.9 (C6-GlcNAc(1,2,3)), 55.4 (C5-Biotin), 55.2, 55.1 (C2-GlcNAc(1,2,3)), 39.7 (C9-Biotin), 39.3, 39.2 (CH_2-Biotin, CH_2-linker), 35.6, 35.5 (CH_2-Biotin), 28.2, 28.0, 27.9, 27.8, 27.6, 25.5, 25.2, 25.0, 22.5 (CH_2-Biotin, CH_2-linker), 22.2 (3x CH_3-Ac).

α2,6-tri-LacNAc-biotin (9)

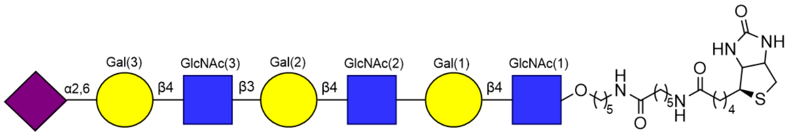

α2,6-tri-LacNAc-biotin

Compound **α2,6-di-LacNAc-biotin** was prepared from compound **tri-LacNAc-biotin** (1.0 mg, 0.65 μmol) using the general procedure for the installation of α2,6-Neu5Ac using ST6GAL1. After P2 purification, **α2,6-di-LacNAc-biotin** was obtained as a white powder (1.1 mg, 92%). ^{1}H NMR (600 MHz, D_2O) δ 4.75 – 4.70 (m, 2H, H1-GlcNAc(2,3)), 4.62 (m, 1H, H8-Biotin), 4.53 (d, J = 7.4 Hz, 1H, H1-GlcNAc(1)), 4.50 – 4.45 (m, 3H, H1-Gal(1,2,3)), 4.44 (dd, J = 8.1, 4.7 Hz, 1H, H6-Biotin), 4.17 (m, 2H, H4-Gal(1,2)), 4.03 – 3.92 (m, 5H, H6a-GlcNAc(1,2,3), H4-Gal(3), H6a-Gal(3)), 3.92 – 3.64 (m, 30H, H5-Sia, H6b-GlcNAc(1,2,3), H6-Gal(1,2), H9-Sia, H4-Sia, H2-GlcNAc(1,2,3), H3-GlcNAc(1,2,3), H5-Gal(1,2,3), H4-Glc(1,2,3), CH_2-linker, H8-Sia, H6-Sia, H3-Gal(1,2,3)), 3.61 – 3.55 (m, 8H, H6b-Gal(3), H7-Sia, H2-Gal(1,2,3), H5-Glc(1,2,3)), 3.35 (m, 1H, H5-Biotin), 3.18 (m, 4H, CH_2-linker, CH_2-Biotin), 3.00 (dd, J = 12.9, 4.9 Hz, 1H, H9a-Biotin), 2.79 (d, J = 13.0 Hz, 1H, H9b-Biotin), 2.68 (dd, J = 12.2, 4.5 Hz, 1H, H3eq-Sia), 2.25 (m, 4H, CH_2-Biotin), 2.04 (m, 12H, 4 x Ac), 1.76 – 1.30 (m, 19H, H3ax-Sia, CH_2-linker, CH_2-Biotin). ^{13}C NMR (151 MHz, D_2O) δ 176.6, 174.6, 175.0, 174.9, 173.6 (C=O), 102.9, 102.8, 102.7 (C1-Gal(1,2,3)), 102.6, 102.5 (C1-GlcNAc(2,3)), 101.0 (C1-GlcNAc(1)), 82.1, 82.0 (C3-Gal(1,2)), 80.5 (C4-GlcNAc(1)), 78.5, 78.1 (C4-GlcNAc(2,3)), 74.9, 74.8, 74.7, 74.5 (C5-Gal(1,2), C5-GlcNAc(1,2,3)), 73.7 (C5-Gal(3)), 72.5, 72,4 (2x), 72.2 (2x) (C6-Sia, C3-GlcNAc(1,2,3), C3-Gal(3)), 71.7 (C8-Sia), 70.7 (C2-Gal(3)), 70.3 (OCH_2-linker), 70.0 (C2-Gal(1,2)), 68.4, 68.3 (2x), 68.2 (C7-Sia, C4-Sia, C4-Gal(1,2,3)), 63.3 C6-Gal(3), 62.7 (C9-Sia), 62.1 (C6-Biotin), 61.0 (C6-Gal(1,2)), 60.3 (C8-Biotin), 60.2 (C6-GlcNAc(1,2,3)), 55.4 (C5-Biotin), 55.1, 55.0, 54.9 (C2-GlcNAc(1,2,3)), 51.9 (C5-Sia), 40.0 (C3-Sia), 39.7 (C9-Biotin), 39.0 (2x) (CH_2-Biotin, CH_2-linker), 35.7, 35.6 (CH_2-Biotin), 28.2, 28.0, 27.9, 27.8, 27.6, 25.5, 25.2, 25.0, 22.5 (CH_2-Biotin, CH_2-linker), 22.3, 22.2, 22.0 (4x CH_3-Ac).

Compound α2,6(Neu5(CHF$_2$CONH-)-tri-LacNAc-biotin (11)

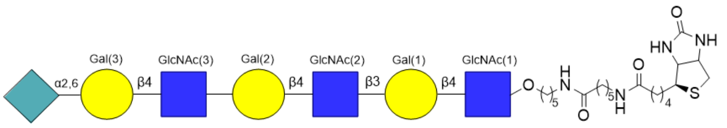

α2,6(Neu5(CHF$_2$CONH-)-tri-LacNAc-biotin

Compound **α2,6(Neu5(CHF$_2$CONH-)-tri-LacNAc-biotin** was prepared from compound **tri-LacNAc-biotin** (1.0 mg, 0.65 µmol) using the general procedure for the installation of α2,6-Neu5Ac using CMP-(Neu5(CHF$_2$CONH-) and ST6GAL1. After P2 purification, **α2,6(Neu5(CHF$_2$CONH-)-tri-LacNAc-biotin** was obtained as a white powder (1.2 mg, 99%). ^{1}H NMR (600 MHz, D$_2$O) δ 6.16 (t, $J_{H\text{-}F}$ = 53.6 Hz, 1H, CHF$_2$), 4.74 – 4.69 (m, 2H, H1-GlcNAc(2,3)), 4.61 (m, 1H, H8-Biotin), 4.52 (d, J = 7.7 Hz, 1H, H1-GlcNAc(1)), 4.48 – 4.44 (m, 3H, H1-Gal(1,2,3)), 4.42 (dd, J = 8.0, 4.3 Hz, 1H, H6-Biotin), 4.16 (m, 2H, H4-Gal(1,2)), 4.03 – 3.93 (m, 6H, H5-Sia, H6a-GlcNAc(1,2,3), H4-Gal(3), H6a-Gal(3)), 3.92 – 3.62 (m, 29H, H6b-GlcNAc(1,2,3), H6-Gal(1,2), H9-Sia, H4-Sia, H2-GlcNAc(1,2,3), H3-GlcNAc(1,2,3), H5-Gal(1,2,3), H4-Glc(1,2,3), CH$_2$-linker, H8-Sia, H6-Sia, H3-Gal(1,2,3)), 3.61 – 3.52 (m, 8H, H6b-Gal(3), H7-Sia, H2-Gal(1,2,3), H5-Glc(1,2,3)), 3.34 (m, 1H, H5-Biotin), 3.17 (m, 4H, CH$_2$-linker, CH$_2$-Biotin), 3.01 (dd, J = 13.0, 4.7 Hz, 1H, H9a-Biotin), 2.79 (d, J = 13.0 Hz, 1H, H9b-Biotin), 2.68 (dd, J = 12.6, 4.8 Hz, 1H, H3eq-Sia), 2.24 (m, 4H, CH$_2$-Biotin), 2.08 – 2.02 (m, 9H, 3 x Ac), 1.77 – 1.27 (m, 19H, H3ax-Sia, CH$_2$-linker, CH$_2$-Biotin). ^{13}C NMR (151 MHz, D$_2$O) δ 176.7, 176.0, 175.1, 174.3, 160.4 (C=O), 108.4 (CHF$_2$), 102.9, 102.8, 102.7 (C1-Gal(1,2,3)), 102.7, 102.6 (C1-GlcNAc(2,3)), 101.0 (C1-GlcNAc(1)), 82.0 (C3-Gal(1,2)), 80.4 (C4-GlcNAc(1)), 78.3, 78.2 (C4-GlcNAc(2,3)), 74.9, 74.7 (C5-Gal(1,2)), 74.5, 74.3 (C5-GlcNAc(1,2,3)), 73.8 (C5-Gal(3)), 72,4, 72.2 (2x), 71.9, 71.8 (C6-Sia, C3-GlcNAc(1,2,3), C3-Gal(3), C8-Sia), 70.7 (C2-Gal(3)), 70.3 (OCH$_2$-linker), 70.0 (C2-Gal(1,2)), 68.3 (2x), 68.0 (C7-Sia, C4-Sia, C4-Gal(1,2,3)), 63.5 (C6-Gal(3)), 62.7 (C9-Sia), 62.1 (C6-Biotin), 61.0 (C6-Gal(1,2)), 60.2 (C8-Biotin), 59.9 (C6-GlcNAc(1,2,3)), 55.3 (C5-Biotin), 55.1, 55.0, 54.9 (C2-GlcNAc(1,2,3)), 51.8 (C5-Sia), 40.0 (C3-Sia), 39.7 (C9-Biotin), 39.0 (2x) (CH$_2$-Biotin, CH$_2$-linker), 35.6, 35.5 (CH$_2$-Biotin), 28.2, 28.0, 27.9, 27.8, 27.6, 25.5, 25.2, 25.1, 22.4 (CH$_2$-Biotin, CH$_2$-linker), 22.3, 22.1 (3x CH$_3$-Ac). HRMS: for $C_{74}H_{122}F_2N_8O_{42}S$: m/z $[M-2H]^{2-}$; calcd: 931.3599; found: 931.3605.

3. Glycan microarray

Supplementary Fig. 4. Probing receptor binding specificities of A(H3N2) virus and control plant lectins. a) Collection of glycans printed on succinimide reactive microarray slides. Glycan binding data of b) HK68, c) NL91 and d) NL03. Whole viruses were exposed to glycan microarray and binding was visualized anti-stalk antibodies. The lectins, e) SNA, f) ECA and g) MALI where precomplexed at 10 µg/mL with an alexa-555 labelled streptavidin at 2 µg/mL. Bars represent the average relative fluorescence units (RFU) of four replicates ± SD.

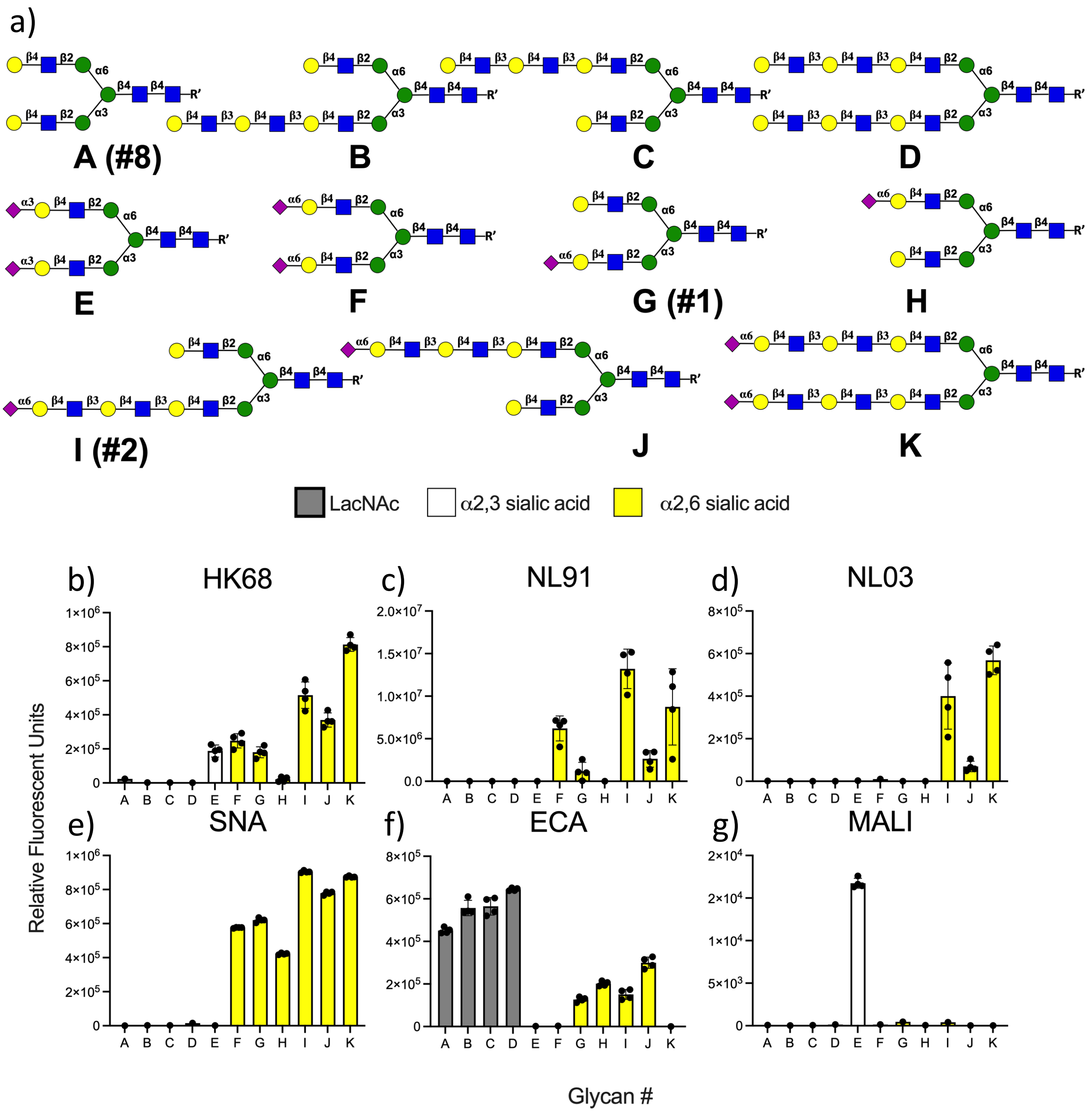

4. NMR competition experiments (Supplementary Fig. 5)

^{1}H-STD NMR and ^{19}F-T_2 relaxation competition experiments with NL91 and NL03.

4.1. Competition ^{1}H-STD NMR

Samples contained 5 µM of protein (NL03/NL91) in D_2O buffer (Tris-d11 20 mM, NaCl 150 mM, pD = 7.4) and 100 eq (0.5 mM) of probe **6**.

Supplementary Fig. 5.A. ^{1}H-STD NMR spectra of the system NL03:6 in the presence of three natural competitors, compounds **7**, **8** and **9**. Spectra show the evolution of the STD signals of the probe and the corresponding competitor in each case, for 0, 0.5, 1, 1.5 and 2 eq. of competitor. Below is detailed the evolution of the Sia-5-N-Acetyl and GlcNAc-2-N-Acetyl peaks from the tested compounds. The STD signal of the Sia-5-N-Acetyl of probe **6** was used in all cases to estimate the dissociation constants (Table below). Both the K_D of the probe (K_L) and the competitors (K_{Comp}) were directly derived from Eq. 1. This equation relates the %STD decay with the amount of competitor added ([C]), using the law of mass action and the Cheng-Prusoff equation (simple competition model) to integrate both K_L and K_{Comp} in the expression. HA cartoon representation was created with BioRender.com, and exported under a paid subscription.

$$\%STD_{decay} = \frac{STD_{probe+comp}}{STD_{probe}} = \frac{\theta_{bound,comp}}{\theta_{bound,init}} = \frac{K_L + L_0 + P_0}{K_L + L_0 + P_0 + \frac{K_L}{K_{Comp}}[C]} \qquad \textbf{Eq. 1}$$

Being P_0 = 5 µM, L_0 = 500 uM. The %STD decay and the concentration of competitor [C] (**7**, **8** or **9**) are the variables for the adjustment. Two fitting parameters are derived: K_L and K_L/K_{Comp}.

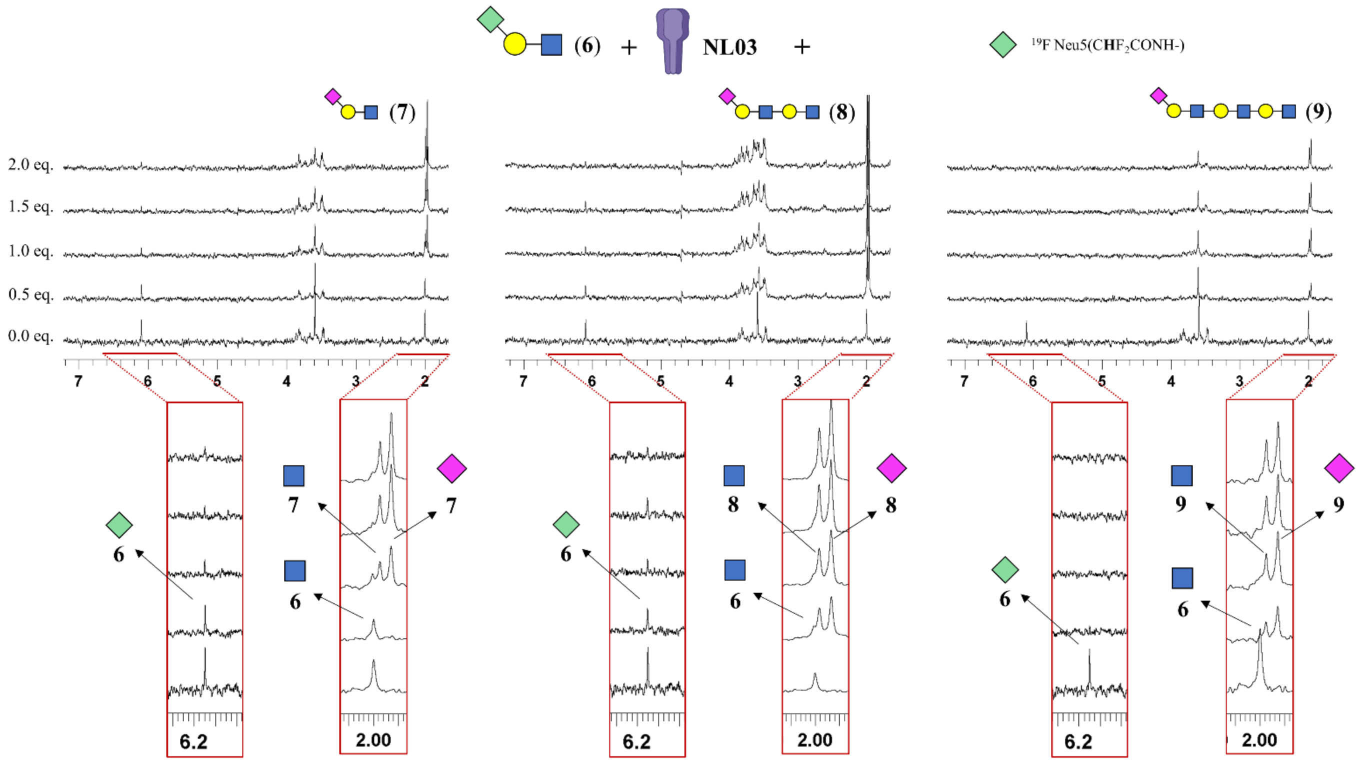

Compound	NL03<br>K_D (µM)
**6**	650-750
**7**	410-620
**8**	400-2000
**9**	25-35

Supplementary Fig. 5.B. ^{1}H-STD NMR spectra of the system NL91:**6** in the presence of three natural competitors, compounds **7**, **8** and **9.** The spectra show the evolution of the STD signals of the probe and the corresponding competitor in each case, for 0, 0.5, 1, 1.5 and 2 eq. of competitor. In this case, the K_Ds could not be estimated due to the low STDs observed for both the probe (**6**) and the competitors. HA cartoon representation was created with BioRender.com, and exported under a paid subscription.

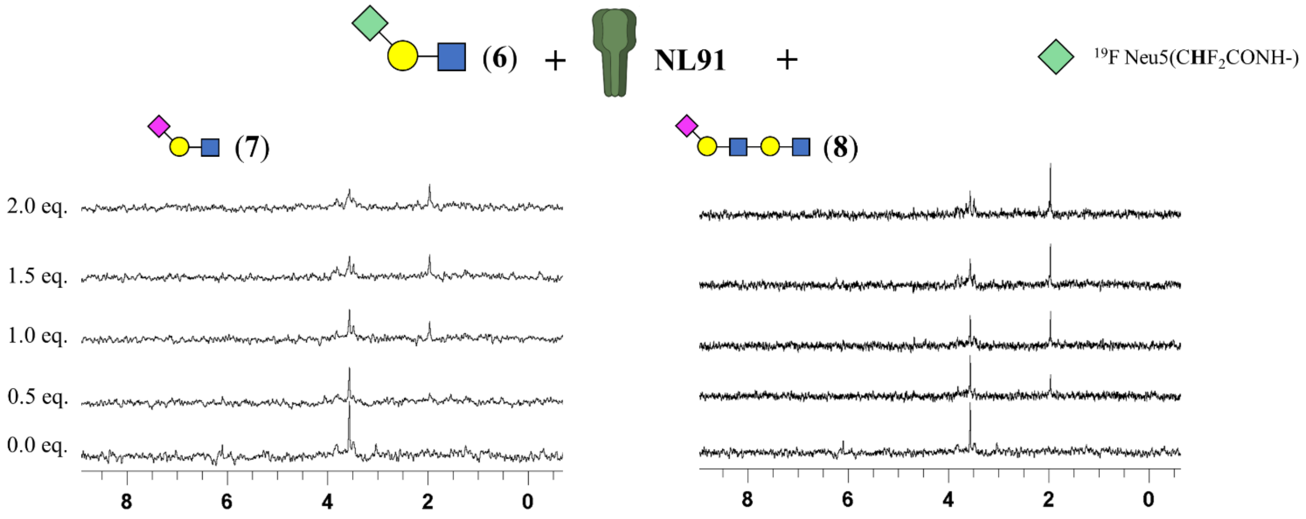

4.2. Competition ^{19}F-T_2 relaxation

Samples contained 5 µM of protein (NL03/NL91) in D_2O buffer (Tris-d11 20 mM, NaCl 150 mM, pD = 7.4) and 24 eq (0.12 mM) of probe **10** or **11**.

Supplementary Fig. 5. C) Top, graphic depiction of the R_2 decay for compound **10** in the presence of NL03**.** The sample contained NL03 5 µM and growing amounts of compound **10** starting from a ratio lectin:ligand = 1:24. In orange is depicted the fitting curve and in purple the R_{2free}. Below, table with the T_2 relaxation times against the same growing amounts of **10**. The K_L was roughly estimated to be ca. 0.77 mM by means of Eq. 3. D) Top, graphic depiction of the R_2 decay for compound **10** in competition with natural compound **9.** The sample contained NL03 5 µM with a fixed amount of compound **10** in a ratio lectin:ligand = 1:24 and growing amounts of compound **9.** In green is depicted the fitting curve and in purple the R_{2free}. Below, table with the T_2 relaxation times of **10** against the same growing amounts of **9** and the ^{19}F peaks at n2τ = 240 ms for all concentrations of 9 used**.** The K_{Comp} was approximated through Eq. 2, considering the values of K_L and R_{2bound} found for the probe (**10**) alone in the previous experiment. E) Table with the observed T_2 relaxation times for compound **11** in the presence of NL91. The sample contained NL91 5 µM

and growing amounts of compound **11** starting from a ratio lectin:ligand = 1:24. Given the low affinities of NL91 to the studied compounds, no noticeable increases were discerned in the observed T_2 and therefore, no curve fitting was possible, rendering the obtention of a K_D impossible. F) Table with the T_2 relaxation times of **11** in the presence of NL91 against growing amounts of competitor **9**, along with the ^{19}F peaks at n2τ = 240 ms for all concentrations of 9 used. The sample contained NL91 5 µM with a fixed amount of compound **11** in a ratio lectin:ligand = 1:24 and growing amounts of compound **9**. HA cartoon representation was created with BioRender.com, and exported under a paid subscription.

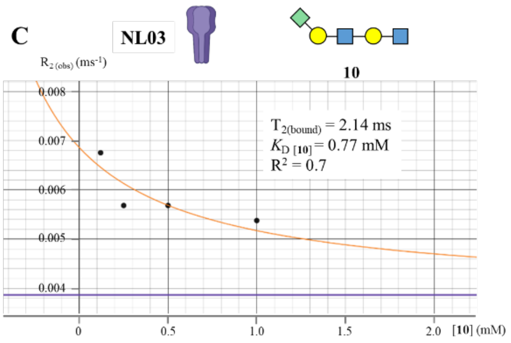

Molecule 10	T_2 (ms)
Free compound	259
1:24	148
1:50	176
1:100	176
1:200	186

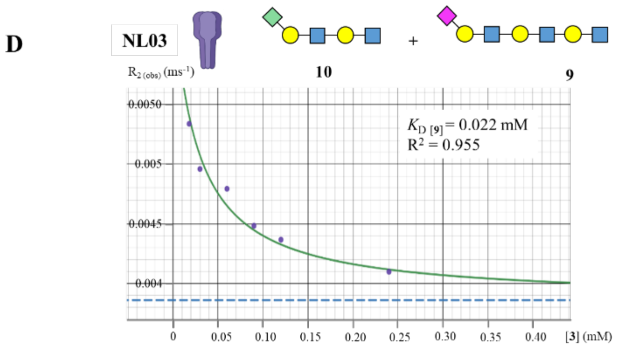

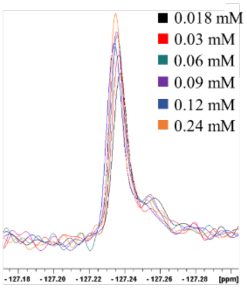

Molecule 9 (mM)	T_2 (ms)
0.018	187
0.03	201
0.06	208
0.09	223
0.12	229
0.24	244

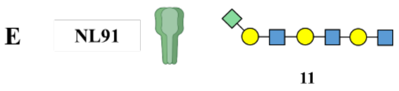

Molecule 10	T_2 (ms)
Free compound	228
1:24	164
1:50	180
1:100	181
1:200	180

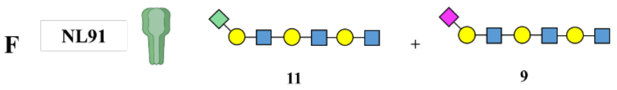

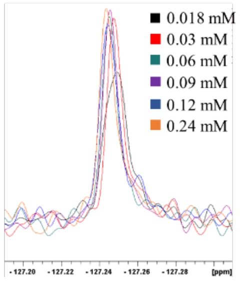

Molecule 9 (mM)	T_2 (ms)
0.018	177
0.03	179
0.06	193
0.09	189
0.12	194
0.24	194

Model used to estimate the K_D of the competitors (K_{Comp}), using compound **10** as spy molecule. This model is an approximation deduced from the law of mass action and works for weak probe binders that fulfil $K_L \geq 10P_0$ (max. ε_r = 2.3%), which is the case for compound **10**.

$$R_{2obs} = R_{2f} + (R_{2b} - R_{2f}) \frac{P_0}{K_L + L_0 + P_0 + \frac{K_L}{K_{Comp}}[C]} \qquad \textit{\textbf{Eq. 2}}$$

Where K_L and L_0 are the dissociation constant and total concentration of spy molecule **10**, respectively, and P_0 is the total concentration of protein, NL03. R_{2obs} ($1/T_{2obs}$) and [C] (concentration of competitor molecule)

are the experimental variables to fit. Data adjustment yields K_{Comp} as fitting parameter. R_{2b} and K_L are estimated a priori through an independent titration of probe **10** at a fixed amount of protein:

$$R_{2obs} = R_{2f} + (R_{2b} - R_{2f})\frac{K_L + P_0 + L_0 - \sqrt{(K_L + P_0 + L_0)^2 - 4L_0P_0}}{2L_0} \qquad \textbf{Eq. 3}$$

Being R_{2obs} and L_0 the experimental variables to evaluate.

4.3. Simulation of ^{19}F-T_2 relaxation

Supplementary Fig. 6. A) Graphical prediction of the expected R_{2obs} decay for the system NL03: **6** according to eqs. 1 and 2, considering a 1:1 ratio of **6**:competitor (dashed vertical purple line) and assuming both K_L and K_{Comp} identical. As shown, for the experimental conditions used (L_0 = 0.125 mM, P_0 = 0.010 mM), the condition K_L/K_{Comp} = 1 yields low percentages of R_2decay (< 5%) only if both ligands are weak binders (low mM to mM). In any other case, ($K_L = K_{Comp}$ < 0.5 mM or $K_L >> K_{Comp}$, Table B), the R_2decay is expected to be noticeably higher. Then, the experimental data presented in this work fits with both **7** and **6** being mM binders, whereas compound **2** must bind approximately one order of magnitude better than the probe (**6**) to produce a decay of ca. 27%, as shown in panel C.

$$\boldsymbol{R_{2obs} = R_{2f} + (R_{2b} - R_{2f})\frac{P_0}{K_L + L_0 + P_0 + \frac{K_L}{K_{Comp}}[C]}} \qquad \textbf{Eq. 1}$$

Being R_{2obs} and [C] the experimental variables to evaluate. Data adjustment yields K_{Comp} as fitting parameter, thus allowing to compare the K_D of the competing compounds. To note, R_{2b} and K_L were estimated a priori through an independent titration of the probe at a fixed amount of protein:

$$R_{2obs} = R_{2f} + (R_{2b} - R_{2f})\frac{K_L + P_0 + L_0 - \sqrt{(K_L + P_0 + L_0)^2 - 4L_0P_0}}{2L_0} \qquad \textbf{Eq. 2}$$

A

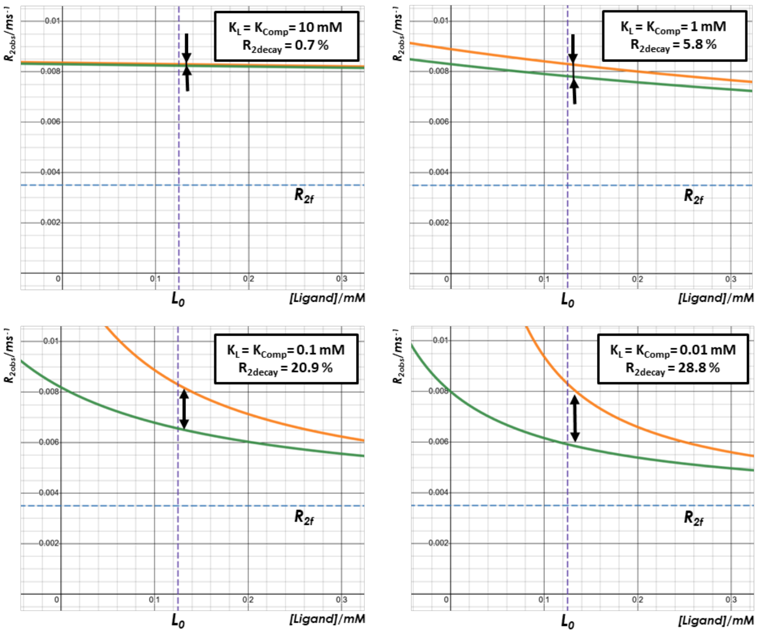

B

K_{Comp}/K_L	K_L (mM)			
	**10**	**1**	**0.1**	**0.01**
**10**	0.1	0.7	4.2	8.0
**1**	0.7	5.8	20.9	28.8
**0.1**	6.4	30.3	48.9	52.2
**0.01**	31.9	53.0	56.8	57.2
**0.001**	53.5	57.3	57.7	57.8

C

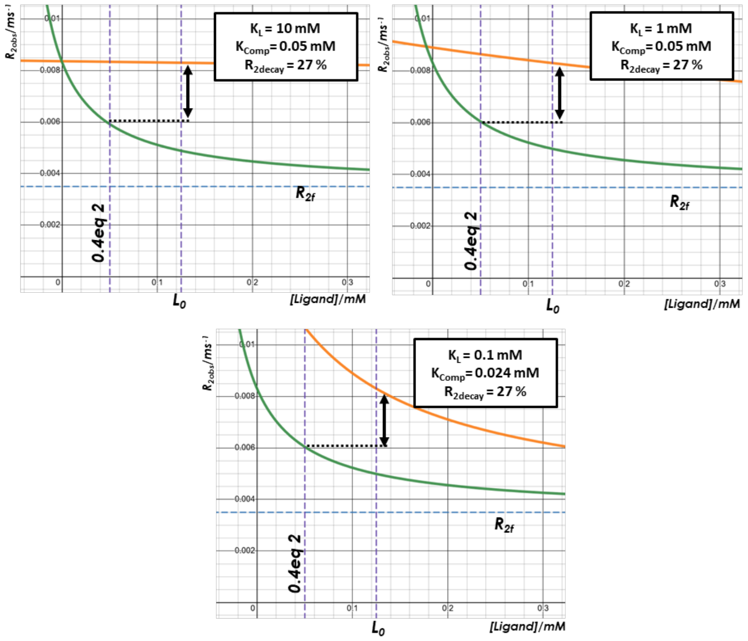

5. 2D STD-^{1}H, ^{13}C-HSQC NMR spectra

5.1. Supplementary Fig. 7. 2D STD-^{1}H,^{13}C-HSQC NMR experiments for the interaction of compound **4** with HA proteins. Top, the off-resonance spectrum and the corresponding STD-HSQC spectra for the three HA proteins in complex with compound **4**. Bottom, sum of STD effects for all signals of the Gal (orange) and the GlcNAc (cyan) of compounds **4** in complex with the three HA proteins.

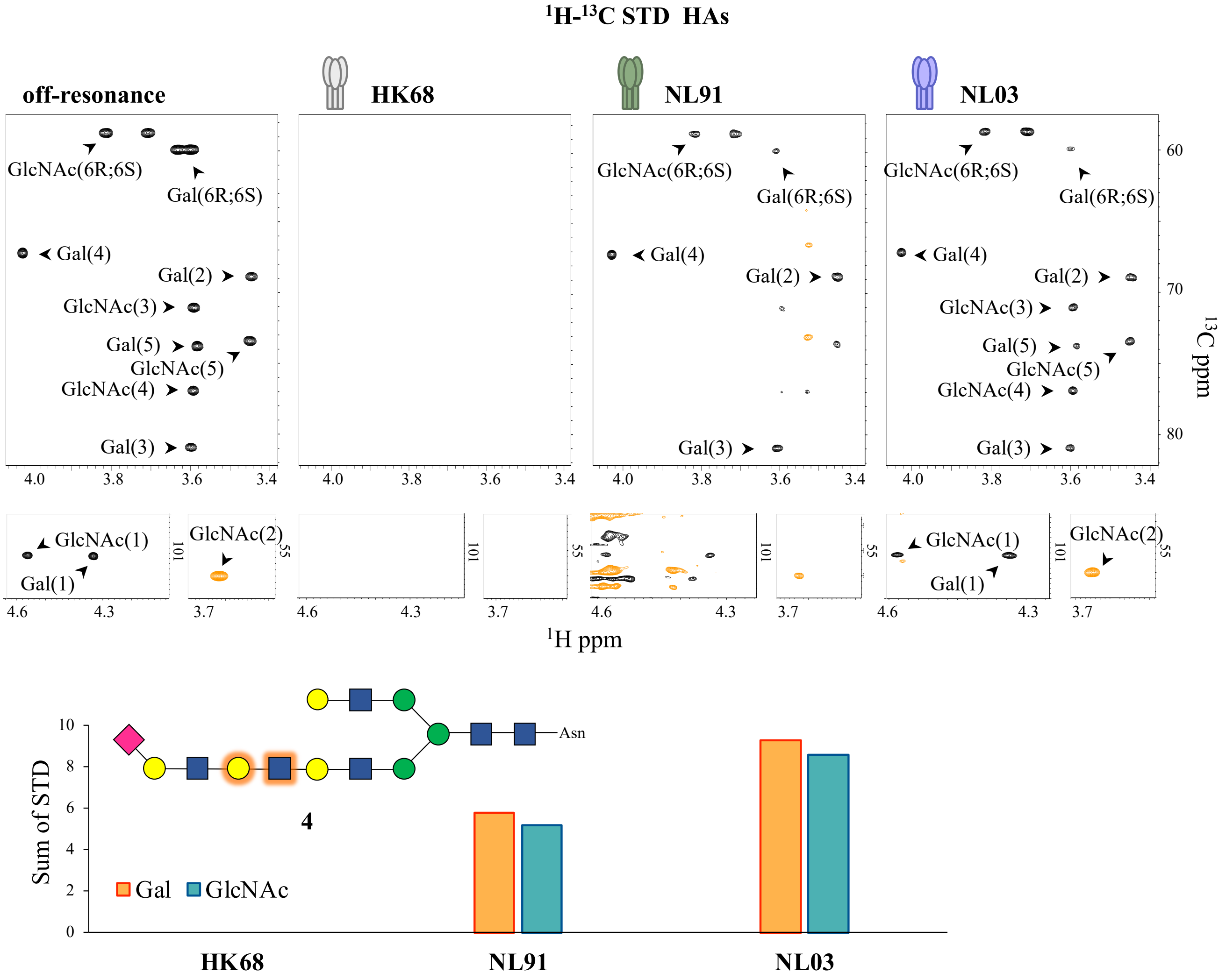

5.2. Supplementary Fig. 8. 2D STD-^{1}H,^{13}C-HSQC NMR experiments for the interaction of compound **5** with HA proteins. Top, the off-resonance spectrum and the corresponding STD-HSQC spectra for the three HA proteins in complex with compound **5**. Bottom, sum of STD effects for all signals of the Gal (orange) of compounds **5** in complex with the three HA proteins.

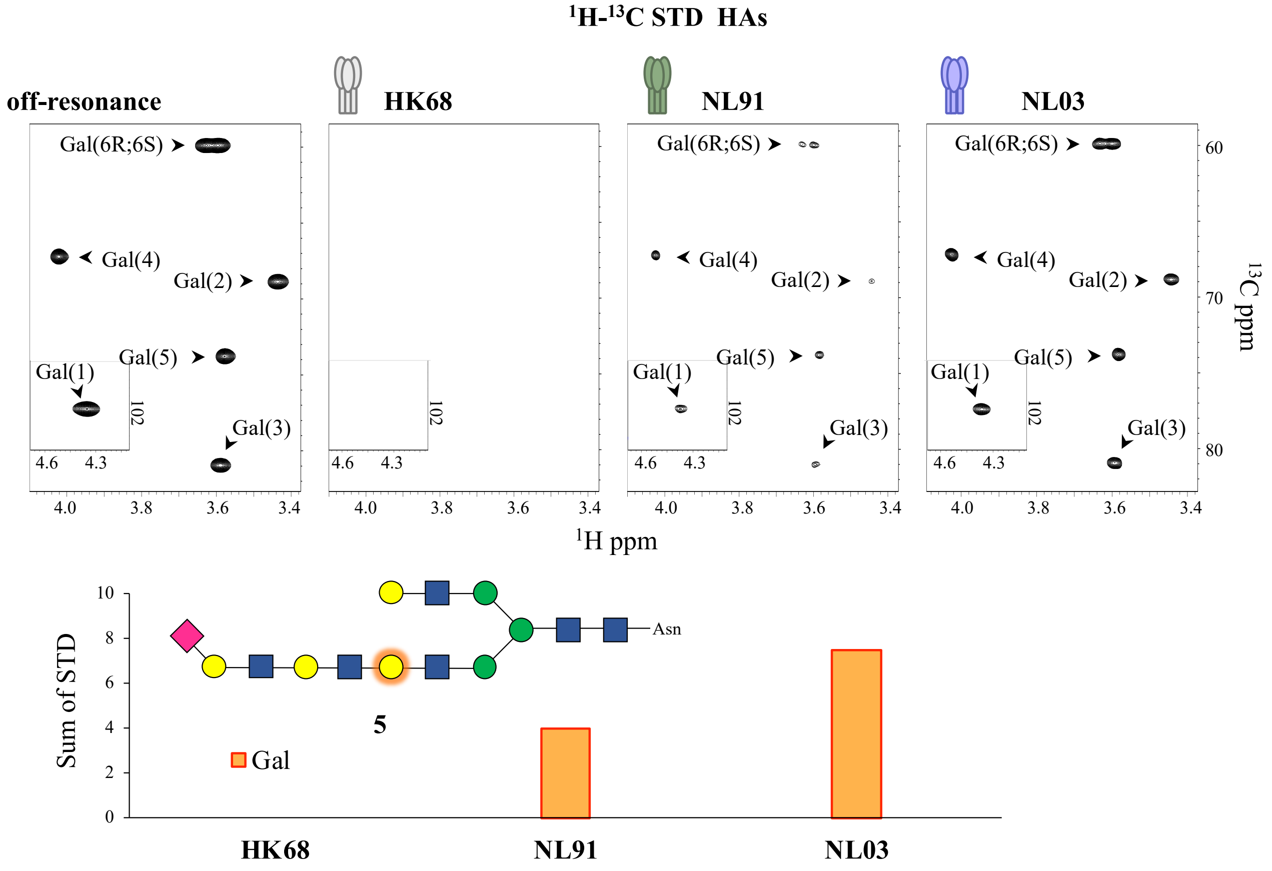

6. Supplementary Fig. 9. Details of 2D STD NMR results for the interaction of compound **5** with HA NL03 protein.

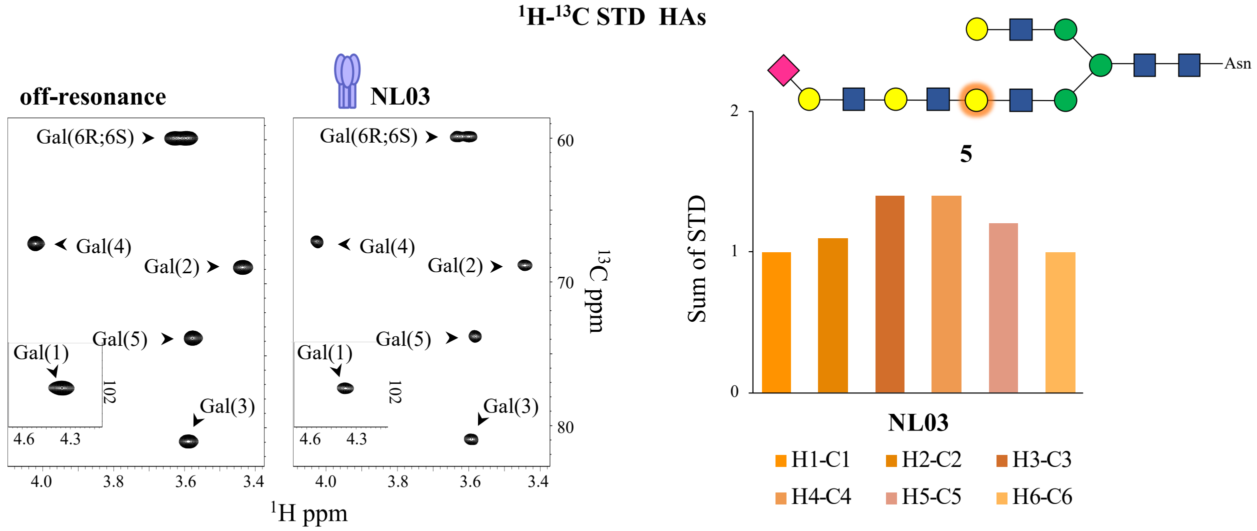

7. Supplementary Fig. 10. Details of the intermolecular interactions between **2** and the HAs proteins as derived by molecular modelling. a) Hong-Kong 1968. b) Netherlands 1991. c) Netherlands 2003. d) Netherlands 2009. e) Singapore 2016.

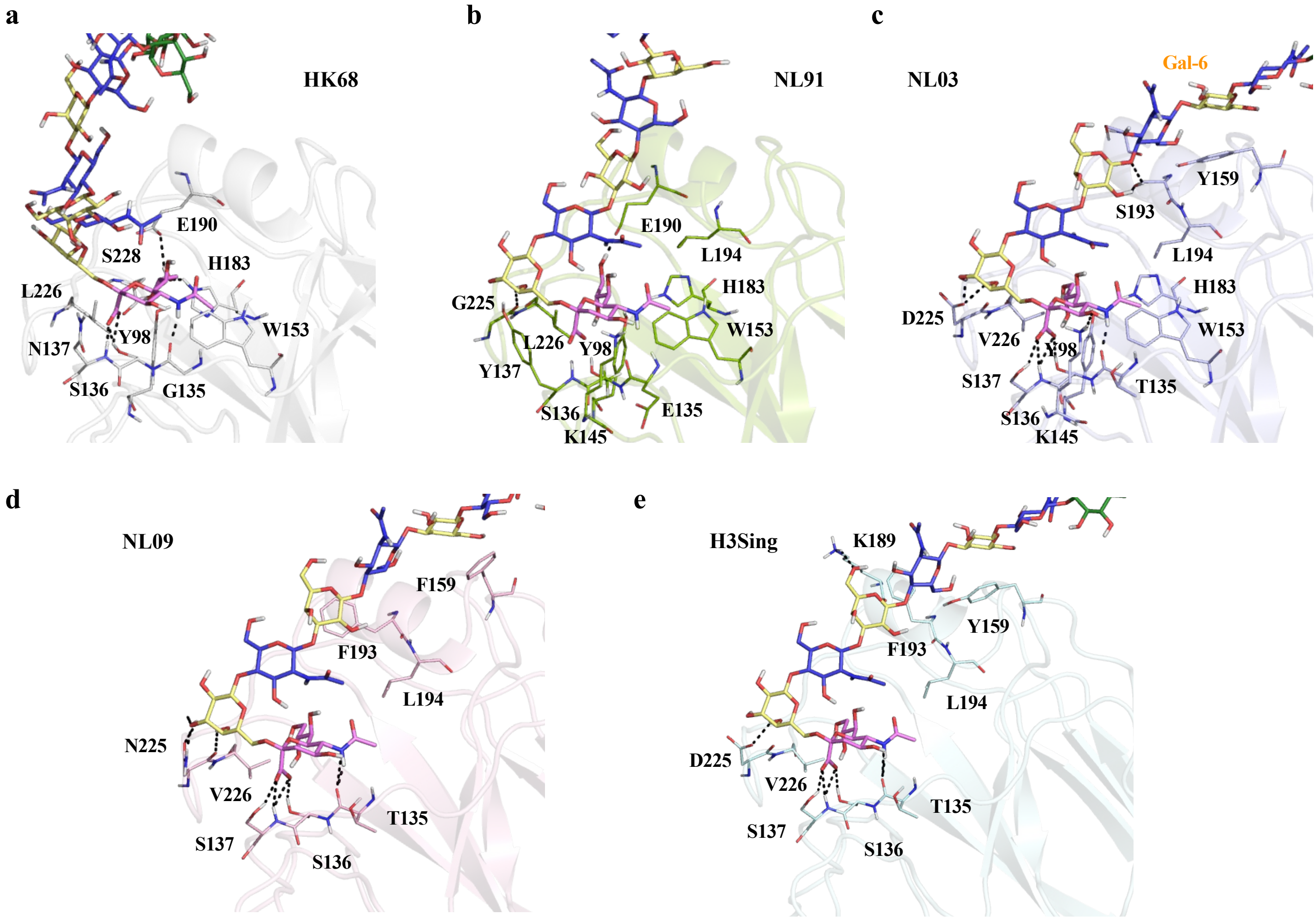

8. Supplementary Fig. 11. Snapshoot of the **2**/HK68 MD trajectory.

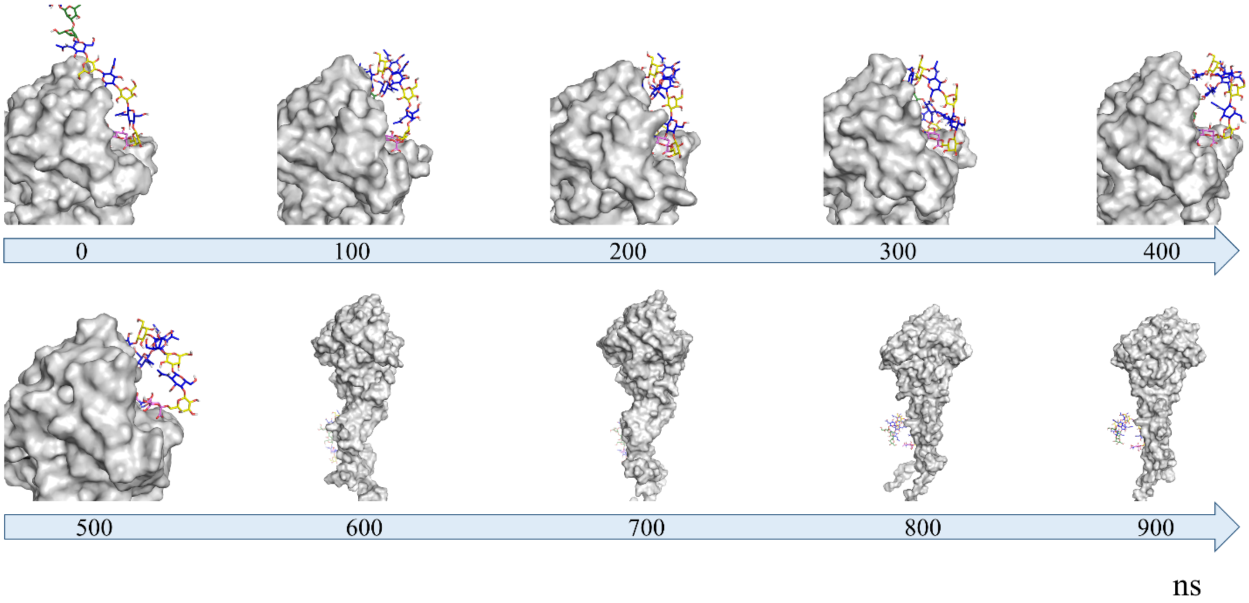

9. Supplementary Fig. 12. Snapshoot of the **2**/NL91 MD trajectory.

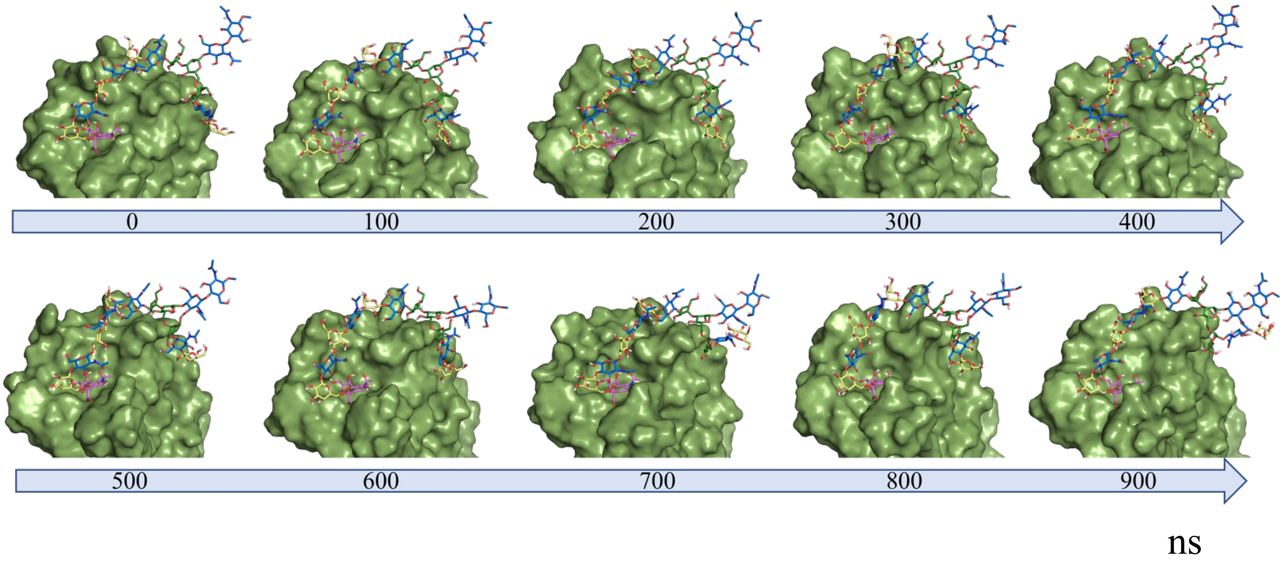

10. Supplementary Fig. 13. Snapshoot of the **2**/NL03 MD trajectory.

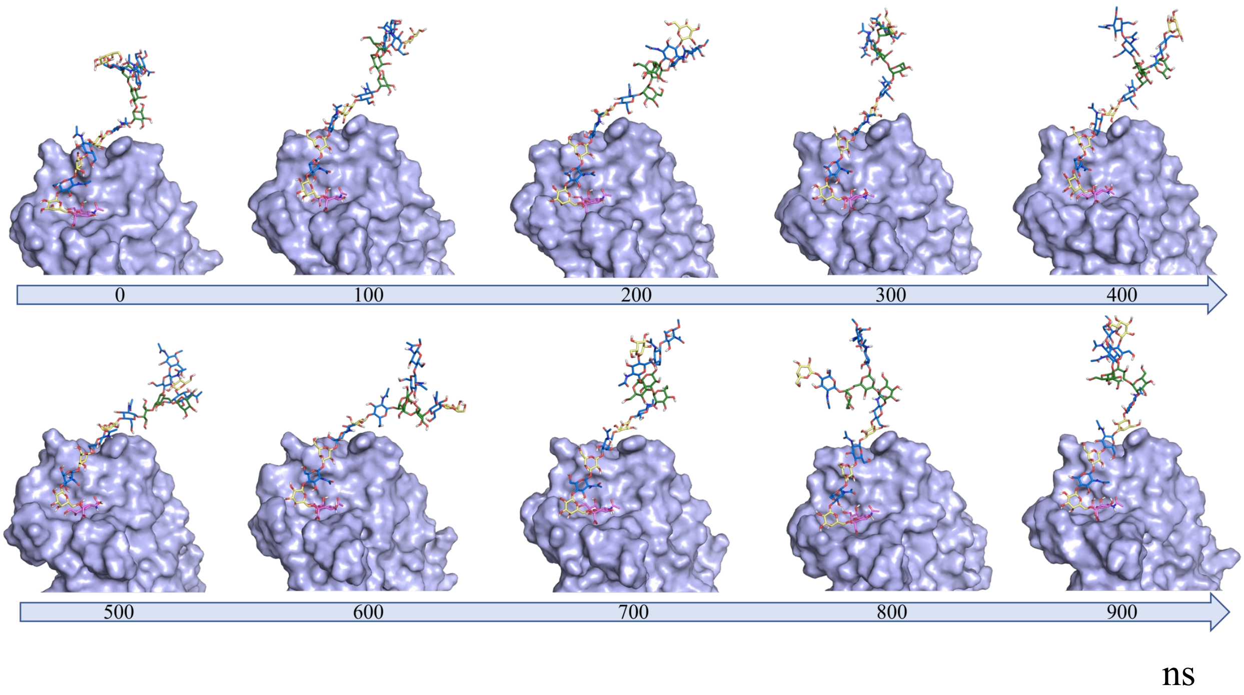

11. Glycan microarray

Supplementary Fig. 14. Probing receptor binding specificities of NL03 mutant proteins. a) A collection of glycans printed on succinimide reactive microarray slides. Glycan binding data of b) NL03 and the mutants c) N137Y, d) S145K and e) N137Y S145K. The recombinant proteins where precomplexed at 100 µg/mL with an anti-streptag antibody at 50 µg/mL and an alexa-555 labelled goat-a-mouse antibody at 25 µg/mL. Bars represent the average relative fluorescence units (RFU) of four replicates ± SD.

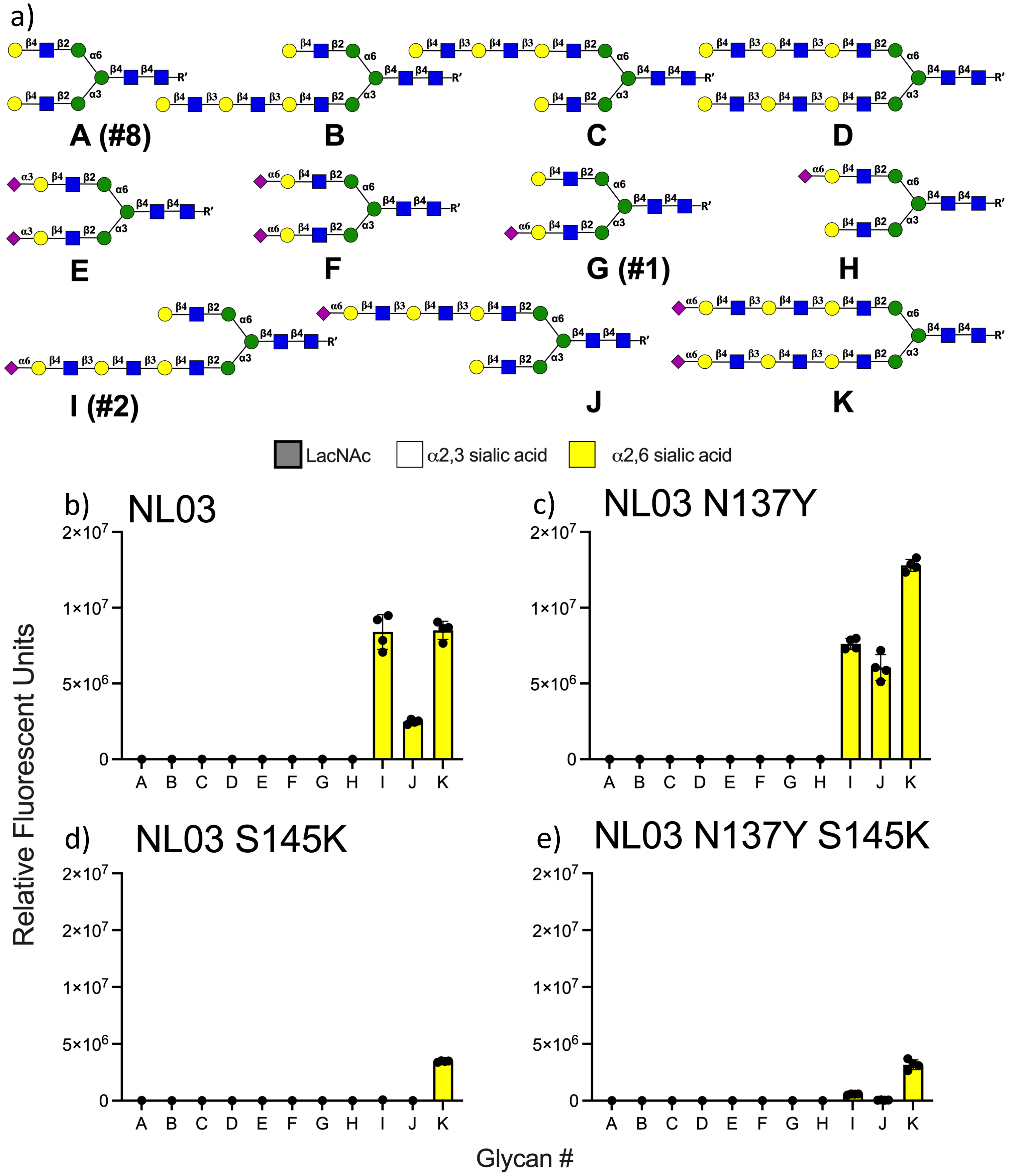

12. References

[1] Liu L, Prudden AR, Bosman GP & Boons GJ Improved isolation and characterization procedure of sialylglycopeptide from egg yolk powder. *Carbohydr. Res.* 452, 122–128 (2017). [PubMed: 29096185]

[2] Liu L, Prudden AR, Capicciotti CJ, Bosman GP, Yang J-Y, Chapla DG, Moremen KW & Boons GJ Streamlining the chemoenzymatic synthesis of complex *N*-glycans by a stop and go strategy. *Nat. Chem.* 11, 161–169 (2019). [PubMed 30532014]
