# Supplementary material for: Probing altered receptor specificities of antigenically drifting human H3N2 viruses by chemoenzymatic synthesis, NMR, and modeling
Source: Nat Commun. 2024 Apr 6;15:2979. doi: 10.1038/s41467-024-47344-y (PMC10998905; doi:10.1038/s41467-024-47344-y)
Supplement: Supplementary file 3 — Reporting Summary [file 41467_2024_47344_MOESM3_ESM.pdf]

nature portfolio

Corresponding author(s): Luca Unione, Robert P. de Vries and Geert-Jan Boons

Last updated by author(s): Mar 1, 2024

Reporting Summary

Nature Portfolio wishes to improve the reproducibility of the work that we publish. This form provides structure for consistency and transparency in reporting. For further information on Nature Portfolio policies, see our Editorial Policies and the Editorial Policy Checklist.

Statistics

For all statistical analyses, confirm that the following items are present in the figure legend, table legend, main text, or Methods section.

n/a	Confirmed	
[ ]	[x]	The exact sample size (*n*) for each experimental group/condition, given as a discrete number and unit of measurement
[ ]	[x]	A statement on whether measurements were taken from distinct samples or whether the same sample was measured repeatedly
[x]	[ ]	The statistical test(s) used AND whether they are one- or two-sided<br>*Only common tests should be described solely by name; describe more complex techniques in the Methods section.*
[x]	[ ]	A description of all covariates tested
[x]	[ ]	A description of any assumptions or corrections, such as tests of normality and adjustment for multiple comparisons
[x]	[ ]	A full description of the statistical parameters including central tendency (e.g. means) or other basic estimates (e.g. regression coefficient) AND variation (e.g. standard deviation) or associated estimates of uncertainty (e.g. confidence intervals)
[x]	[ ]	For null hypothesis testing, the test statistic (e.g. *F*, *t*, *r*) with confidence intervals, effect sizes, degrees of freedom and *P* value noted<br>*Give P values as exact values whenever suitable.*
[x]	[ ]	For Bayesian analysis, information on the choice of priors and Markov chain Monte Carlo settings
[x]	[ ]	For hierarchical and complex designs, identification of the appropriate level for tests and full reporting of outcomes
[x]	[ ]	Estimates of effect sizes (e.g. Cohen's *d*, Pearson's *r*), indicating how they were calculated

Our web collection on statistics for biologists contains articles on many of the points above.

Software and code

Policy information about availability of computer code

Data collection	LC-MS data acquisition was performed using Agilent MassHunter Workstation Data Acquisition B.09 software.<br>NMR data were collected using BRUKER TopSpin 3.2.7..<br>MD simulation data were acquired using AMBER16 program4.
Data analysis	NMR data were analyzed using BRUKER TopSpin 3.2.7 & Topspin 3.5.<br>PNNL preprocessor v2017.11, IM-MS preprocessor and Agilent MassHunter IM-MS browser 8.0.<br>Glycomod tool https://web.expasy.org/glycomod/.<br>cpptraj module in Amber-Tools 16.<br>Microsoft Excel (Office2016).

For manuscripts utilizing custom algorithms or software that are central to the research but not yet described in published literature, software must be made available to editors and reviewers. We strongly encourage code deposition in a community repository (e.g. GitHub). See the Nature Portfolio guidelines for submitting code & software for further information.

Data

Policy information about availability of data

All manuscripts must include a data availability statement. This statement should provide the following information, where applicable:

- Accession codes, unique identifiers, or web links for publicly available datasets
- A description of any restrictions on data availability
- For clinical datasets or third party data, please ensure that the statement adheres to our policy

The authors confirm that the data supporting the findings of this study are available within the article and/or its supplementary materials.
Data availability
The authors declare that the data supporting the findings of this study are available within the paper and its supplementary information files. The sequence and 3D structures of HA of HK68 and NL03 were derived from https://www.rcsb.org/ under the access code 6TZB and 6BKR, respectively.
The 3D structures of NL91, NL09, Sing16 and Sing16(Y159A) were derived from [http:// 3dflu.cent.uw.edu.pl/index.html].

Research involving human participants, their data, or biological material

Policy information about studies with human participants or human data. See also policy information about sex, gender (identity/presentation), and sexual orientation and race, ethnicity and racism.

Reporting on sex and gender	N/A
Reporting on race, ethnicity, or other socially relevant groupings	N/A
Population characteristics	N/A
Recruitment	N/A
Ethics oversight	N/A

Note that full information on the approval of the study protocol must also be provided in the manuscript.

Field-specific reporting

Please select the one below that is the best fit for your research. If you are not sure, read the appropriate sections before making your selection.

☒ Life sciences ☐ Behavioural & social sciences ☐ Ecological, evolutionary & environmental sciences

For a reference copy of the document with all sections, see nature.com/documents/nr-reporting-summary-flat.pdf

Life sciences study design

All studies must disclose on these points even when the disclosure is negative.

Sample size	Protein concentrations were calculated by reading the absorbance at 280 nm by a nanodrop (NanoDrop One thermo scientific) divided by the molar extintion coefficient (epsilon at 280).<br>Glycan concentrations were calculated by dividing the mass of pure compounds by the molecular weight and the volume. The concentrations of the glycans was determined gravimetrically and confirmed by NMR using TSP as internal standard.
Data exclusions	No data was excluded from the analyses
Replication	19F-NMR experiments and glycan micro-array were performed in replicates. 1D 1H-STD, & 2D 1H-STD-1H,13C-HSQC experiments were performed in replicates, in which parameters, such as temperature, protein irradiation frequencies, relaxation delay were changed and performed independently. All attempts at replication were successful.
Randomization	No randomization was carried out. The experimenter has no subjective influence on the outcome of NMR, glycan microarray, and MD simulation analyses.
Blinding	Experimenter were blinded while performing NMR and Glycan micro-array experiments. The samples were prepared by one person and the experiments analyzed by a second person. Blinding was not possible and not relevant for MD simulations since the experimenter has no subjective influence on the outcome.

Behavioural & social sciences study design

All studies must disclose on these points even when the disclosure is negative.

Study description	*Briefly describe the study type including whether data are quantitative, qualitative, or mixed-methods (e.g. qualitative cross-sectional, quantitative experimental, mixed-methods case study).*
Research sample	*State the research sample (e.g. Harvard university undergraduates, villagers in rural India) and provide relevant demographic information (e.g. age, sex) and indicate whether the sample is representative. Provide a rationale for the study sample chosen. For studies involving existing datasets, please describe the dataset and source.*
Sampling strategy	*Describe the sampling procedure (e.g. random, snowball, stratified, convenience). Describe the statistical methods that were used to predetermine sample size OR if no sample-size calculation was performed, describe how sample sizes were chosen and provide a rationale for why these sample sizes are sufficient. For qualitative data, please indicate whether data saturation was considered, and what criteria were used to decide that no further sampling was needed.*
Data collection	*Provide details about the data collection procedure, including the instruments or devices used to record the data (e.g. pen and paper, computer, eye tracker, video or audio equipment) whether anyone was present besides the participant(s) and the researcher, and whether the researcher was blind to experimental condition and/or the study hypothesis during data collection.*
Timing	*Indicate the start and stop dates of data collection. If there is a gap between collection periods, state the dates for each sample cohort.*
Data exclusions	*If no data were excluded from the analyses, state so OR if data were excluded, provide the exact number of exclusions and the rationale behind them, indicating whether exclusion criteria were pre-established.*
Non-participation	*State how many participants dropped out/declined participation and the reason(s) given OR provide response rate OR state that no participants dropped out/declined participation.*
Randomization	*If participants were not allocated into experimental groups, state so OR describe how participants were allocated to groups, and if allocation was not random, describe how covariates were controlled.*

Ecological, evolutionary & environmental sciences study design

All studies must disclose on these points even when the disclosure is negative.

Study description	*Briefly describe the study. For quantitative data include treatment factors and interactions, design structure (e.g. factorial, nested, hierarchical), nature and number of experimental units and replicates.*
Research sample	*Describe the research sample (e.g. a group of tagged Passer domesticus, all Stenocereus thurberi within Organ Pipe Cactus National Monument), and provide a rationale for the sample choice. When relevant, describe the organism taxa, source, sex, age range and any manipulations. State what population the sample is meant to represent when applicable. For studies involving existing datasets, describe the data and its source.*
Sampling strategy	*Note the sampling procedure. Describe the statistical methods that were used to predetermine sample size OR if no sample-size calculation was performed, describe how sample sizes were chosen and provide a rationale for why these sample sizes are sufficient.*
Data collection	*Describe the data collection procedure, including who recorded the data and how.*
Timing and spatial scale	*Indicate the start and stop dates of data collection, noting the frequency and periodicity of sampling and providing a rationale for these choices. If there is a gap between collection periods, state the dates for each sample cohort. Specify the spatial scale from which the data are taken*
Data exclusions	*If no data were excluded from the analyses, state so OR if data were excluded, describe the exclusions and the rationale behind them, indicating whether exclusion criteria were pre-established.*
Reproducibility	*Describe the measures taken to verify the reproducibility of experimental findings. For each experiment, note whether any attempts to repeat the experiment failed OR state that all attempts to repeat the experiment were successful.*
Randomization	*Describe how samples/organisms/participants were allocated into groups. If allocation was not random, describe how covariates were controlled. If this is not relevant to your study, explain why.*
Blinding	*Describe the extent of blinding used during data acquisition and analysis. If blinding was not possible, describe why OR explain why blinding was not relevant to your study.*

Did the study involve field work? ☐ Yes ☐ No

Field work, collection and transport

Field conditions	*Describe the study conditions for field work, providing relevant parameters (e.g. temperature, rainfall).*
Location	*State the location of the sampling or experiment, providing relevant parameters (e.g. latitude and longitude, elevation, water depth).*
Access & import/export	*Describe the efforts you have made to access habitats and to collect and import/export your samples in a responsible manner and in compliance with local, national and international laws, noting any permits that were obtained (give the name of the issuing authority, the date of issue, and any identifying information).*
Disturbance	*Describe any disturbance caused by the study and how it was minimized.*

Reporting for specific materials, systems and methods

We require information from authors about some types of materials, experimental systems and methods used in many studies. Here, indicate whether each material, system or method listed is relevant to your study. If you are not sure if a list item applies to your research, read the appropriate section before selecting a response.

Materials & experimental systems

n/a	Involved in the study	
☐	☒	Antibodies
☐	☒	Eukaryotic cell lines
☒	☐	Palaeontology and archaeology
☒	☐	Animals and other organisms
☒	☐	Clinical data
☒	☐	Dual use research of concern
☒	☐	Plants

Methods

n/a	Involved in the study	
☒	☐	ChIP-seq
☒	☐	Flow cytometry
☒	☐	MRI-based neuroimaging

Antibodies

Antibodies used	mouse anti-streptag and CR8020 are made in house, goat anti-human alexa 648 Thermo A-21445, goat anti-human alexa 555 Thermo A-21433
Validation	None

Eukaryotic cell lines

Policy information about cell lines and Sex and Gender in Research

Cell line source(s)	Both HEK 293T (CRL-11268) and HEK 293S GNTI(-) (ATCC CRL-3022) cells were employed for the expression of recombinant proteins.
Authentication	*Describe the authentication procedures for each cell line used OR declare that none of the cell lines used were authenticated.*
Mycoplasma contamination	None
Commonly misidentified lines (See ICLAC register)	*Name any commonly misidentified cell lines used in the study and provide a rationale for their use.*

Palaeontology and Archaeology

Specimen provenance	N/A
Specimen deposition	N/A
Dating methods	N/A

☒ Tick this box to confirm that the raw and calibrated dates are available in the paper or in Supplementary Information.

Ethics oversight	N/A

Note that full information on the approval of the study protocol must also be provided in the manuscript.

Animals and other research organisms

Policy information about studies involving animals; ARRIVE guidelines recommended for reporting animal research, and Sex and Gender in Research

Laboratory animals	N/A
Wild animals	N/A
Reporting on sex	N/A
Field-collected samples	N/A
Ethics oversight	N/A

Note that full information on the approval of the study protocol must also be provided in the manuscript.

Clinical data

Policy information about clinical studies

All manuscripts should comply with the ICMJEguidelines for publication of clinical research and a completedCONSORT checklist must be included with all submissions.

Clinical trial registration	N/A
Study protocol	N/A
Data collection	N/A
Outcomes	N/A

Dual use research of concern

Policy information about dual use research of concern

Hazards

Could the accidental, deliberate or reckless misuse of agents or technologies generated in the work, or the application of information presented in the manuscript, pose a threat to:

No	Yes	
[x]	[ ]	Public health
[x]	[ ]	National security
[x]	[ ]	Crops and/or livestock
[x]	[ ]	Ecosystems
[x]	[ ]	Any other significant area

Experiments of concern

Does the work involve any of these experiments of concern:

No	Yes	
[x]	[ ]	Demonstrate how to render a vaccine ineffective
[x]	[ ]	Confer resistance to therapeutically useful antibiotics or antiviral agents
[x]	[ ]	Enhance the virulence of a pathogen or render a nonpathogen virulent
[x]	[ ]	Increase transmissibility of a pathogen
[x]	[ ]	Alter the host range of a pathogen
[x]	[ ]	Enable evasion of diagnostic/detection modalities
[x]	[ ]	Enable the weaponization of a biological agent or toxin
[x]	[ ]	Any other potentially harmful combination of experiments and agents

Plants

Seed stocks	N/A
Novel plant genotypes	N/A
Authentication	N/A

ChIP-seq

Data deposition

- [x] Confirm that both raw and final processed data have been deposited in a public database such as GEO.
- [x] Confirm that you have deposited or provided access to graph files (e.g. BED files) for the called peaks.

Data access links<br>*May remain private before publication.*	N/A
Files in database submission	N/A
Genome browser session<br>(e.g. UCSC)	N/A

Methodology

Replicates	N/A
Sequencing depth	N/A
Antibodies	N/A
Peak calling parameters	N/A
Data quality	N/A
Software	N/A

Flow Cytometry

Plots

Confirm that:

- [x] The axis labels state the marker and fluorochrome used (e.g. CD4-FITC).
- [x] The axis scales are clearly visible. Include numbers along axes only for bottom left plot of group (a 'group' is an analysis of identical markers).
- [x] All plots are contour plots with outliers or pseudocolor plots.
- [x] A numerical value for number of cells or percentage (with statistics) is provided.

Methodology

Sample preparation	N/A
Instrument	N/A
Software	N/A
Cell population abundance	N/A

Gating strategy: N/A

☐ Tick this box to confirm that a figure exemplifying the gating strategy is provided in the Supplementary Information.

Magnetic resonance imaging

Experimental design

Design type	N/A
Design specifications	N/A
Behavioral performance measures	N/A

Acquisition

Imaging type(s)	N/A
Field strength	N/A
Sequence & imaging parameters	N/A
Area of acquisition	N/A

Diffusion MRI ☐ Used ☒ Not used

Preprocessing

Preprocessing software	N/A
Normalization	N/A
Normalization template	N/A
Noise and artifact removal	N/A
Volume censoring	N/A

Statistical modeling & inference

Model type and settings	N/A
Effect(s) tested	N/A

Specify type of analysis: ☐ Whole brain ☐ ROI-based ☐ Both

Statistic type for inference (See Eklund et al. 2016)	N/A
Correction	N/A

Models & analysis

n/a	Involved in the study
☐	☐ Functional and/or effective connectivity
☐	☐ Graph analysis
☐	☐ Multivariate modeling or predictive analysis

Functional and/or effective connectivity	N/A
Graph analysis	N/A
Multivariate modeling and predictive analysis	N/A
